# Supplementary material for: Unveiling the power of high-dimensional cytometry data with cyCONDOR
Source: Nat Commun. 2024 Dec 19;15:10702. doi: 10.1038/s41467-024-55179-w (PMC11659560; doi:10.1038/s41467-024-55179-w)
Supplement: Supplementary file 1 — Supplementary Information [file 41467_2024_55179_MOESM1_ESM.pdf]

Figure S1

a

	cyCONDOR	cytofkit	SPECTRE	Catalyst	tidyTOF
data loading and pre-processing	✓	✓	✓	✓	✓
dimensionality reduction	✓	✓	✓	✓	✓
clustering	✓	✓	✓	✓	✓
data integration	✓	✗	✓	✗	✓
differential analysis	✓	✗	✓	✓	✓
pseudotime analysis	✓	✗	✗	✗	✗
data projection	✓	✗	✗	✗	✗
label transfer	✓	✗	✓	✗	✓
ML classifier	✓	✗	✗	✗	✓
Tested on: HDFC, Cytof , SpectralFlow and CITE-seq	✓	✗	✗	✗	✗
code availability	✓	✓	✓	✓	✓
active development	✓	✗	✓	✓	✓

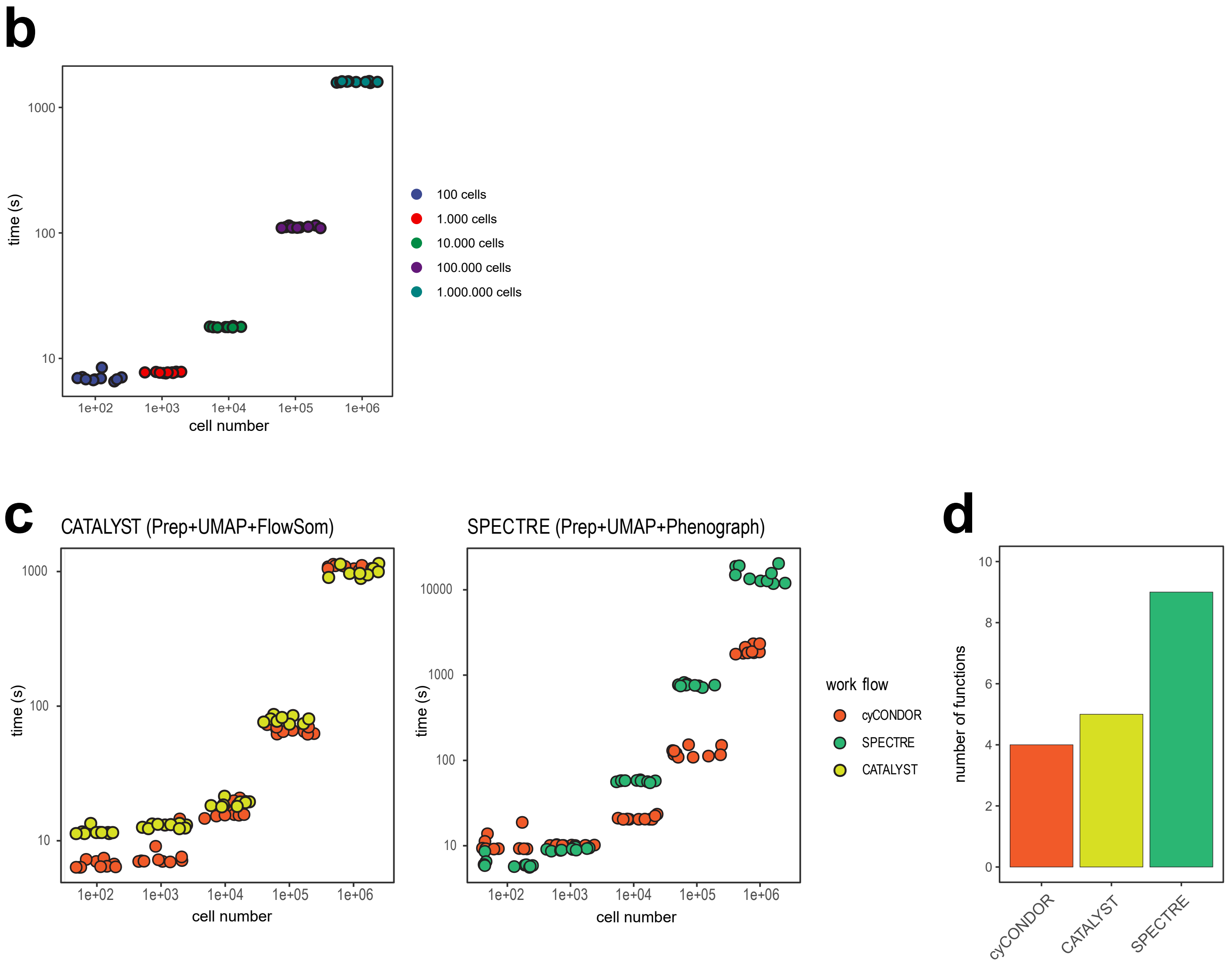

Figure S1

a, Comparative table of *cyCONDOR* with the most diffused cytometry data analysis frameworks. **b,** Performance analysis of the cyCONDOR workflow; data loading and transformation, PCA, UMAP and Phenograph clustering were performed on different numbers of cells (100, 1000, 10000, 100000, 1000000) for ten times each. The result show linear scaling of the cyCONDOR ecosystem. **c,** Performance comparison of the cyCONDOR workflow against CATALYST (v.1.24.0, left) and SPECTRE (v.1.1.0, right), data loading, downsampling and transformation, PCA, UMAP and clustering (FlowSOM for CATALYST and Phenograph for SPECTRE) were performed on different numbers of cells (100, 1000, 10000, 100000, 1000000) for ten times each. All measurement were taken using the cyCONDOR Docker image (v0.1.5) on a Windows 10 workstation equipped with Intel Core i7-8700K CPU and 32 Gb or system memory. **d,** Comparison of the number of functions needed for data loading downsampling and transformation, PCA, UMAP and clustering in cyCONDOR, CATALYST and SPECTRE. Source data are provided as a Source Data file.

Figure S2

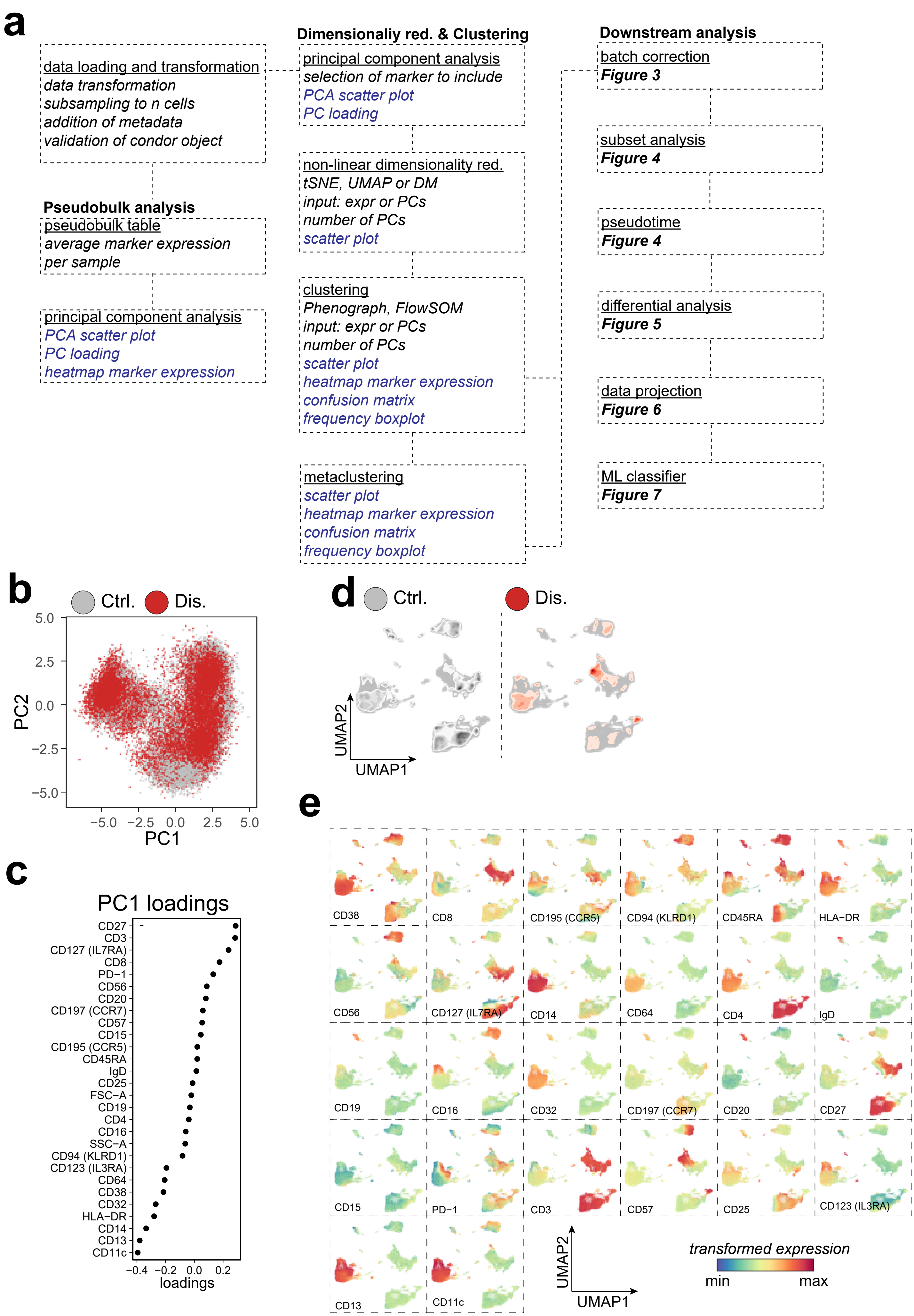

a
Dimensionaliy red. & Clustering
Downstream analysis
data loading and transformation
data transformation
subsampling to n cells
addition of metadata
validation of condor object
Pseudobulk analysis
pseudobulk table
average marker expression
per sample
principal component analysis
PCA scatter plot
PC loading
heatmap marker expression
principal component analysis
selection of marker to include
PCA scatter plot
PC loading
non-linear dimensionality red.
tSNE, UMAP or DM
input: expr or PCs
number of PCs
scatter plot
clustering
Phenograph, FlowSOM
input: expr or PCs
number of PCs
scatter plot
heatmap marker expression
confusion matrix
frequency boxplot
metaclustering
scatter plot
heatmap marker expression
confusion matrix
frequency boxplot
batch correction
Figure 3
subset analysis
Figure 4
pseudotime
Figure 4
differential analysis
Figure 5
data projection
Figure 6
ML classifier
Figure 7
b
Ctrl.
Dis.
PC2
PC1
5.0
2.5
0.0
−2.5
−5.0
−5.0
−2.5
0.0
2.5
5.0
c
PC1 loadings
CD27
CD3
CD127 (IL7RA)
CD8
PD−1
CD56
CD20
CD197 (CCR7)
CD57
CD15
CD195 (CCR5)
CD45RA
IgD
CD25
FSC−A
CD19
CD4
CD16
SSC−A
CD94 (KLRD1)
CD123 (IL3RA)
CD64
CD38
CD32
HLA−DR
CD14
CD13
CD11c
−0.4
−0.2
0.0
0.2
loadings
d
Ctrl.
Dis.
UMAP2
UMAP1
e
CD38
CD8
CD195 (CCR5)
CD94 (KLRD1)
CD45RA
HLA-DR
CD56
CD127 (IL7RA)
CD14
CD64
CD4
IgD
CD19
CD16
CD32
CD197 (CCR7)
CD20
CD27
CD15
PD−1
CD3
CD57
CD25
CD123 (IL3RA)
CD13
CD11c
UMAP2
UMAP1
transformed expression
min
max

Figure S2

a, Detailed schematic of *cyCONDOR* preprocessing and downstream analysis. **b,** Scatterplot of single-cell level PC coordinates colored by experimental group. **c,** Loading of the first PC. **d,** UMAP colored according to the density of cells of each experimental group. **e,** UMAP colored according to the transformed expression of each marker in the dataset.

Figure S3

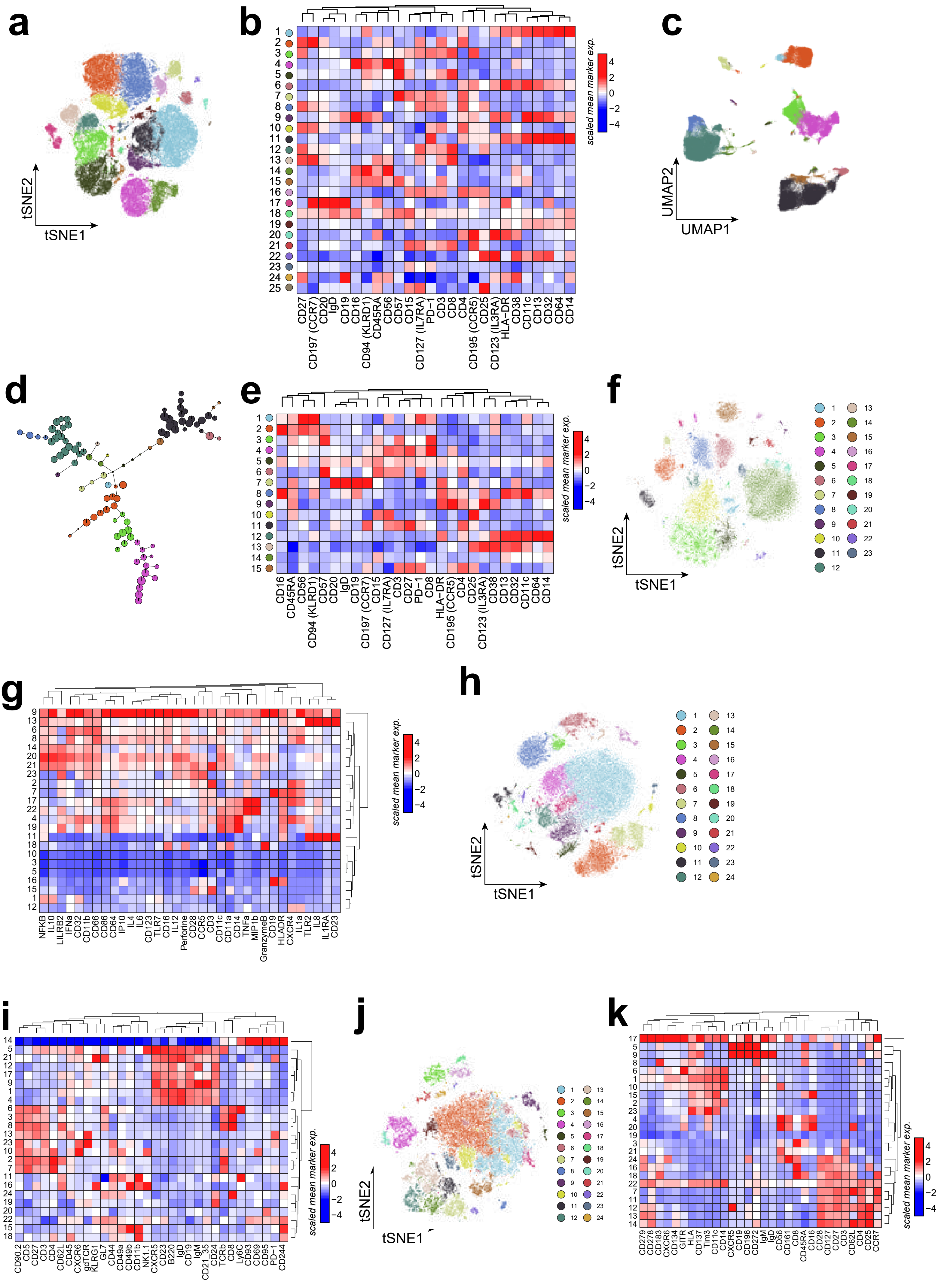
a
tSNE2
tSNE1
b
CD27
CD197 (CCR7)
CD20
IgD
CD19
CD16
CD94 (KLRD1)
CD45RA
CD56
CD57
CD15
CD127 (IL7RA)
PD-1
CD3
CD8
CD4
CD195 (CCR5)
CD25
CD123 (IL3RA)
HLA-DR
CD38
CD11c
CD13
CD32
CD64
CD14
scaled mean marker exp.
c
UMAP2
UMAP1
d
e
CD16
CD45RA
CD56
CD94 (KLRD1)
CD57
CD20
IgD
CD19
CD197 (CCR7)
CD15
CD127 (IL7RA)
CD3
CD27
PD-1
CD8
HLA-DR
CD195 (CCR5)
CD4
CD25
CD123 (IL3RA)
CD38
CD13
CD32
CD11c
CD64
CD14
scaled mean marker exp.
f
tSNE2
tSNE1
g
NFKB
IL10
LILRB2
IFNa
CD32
CD11b
CD66
CD86
CD64
IP10
IL4
IL6
CD123
TLR7
CD16
IL12
Perforine
CD28
CCR5
CD3
CD11c
CD11a
CD14
TNFa
MIP1b
GranzymeB
CD19
HLADR
CXCR4
IL1a
TLR2
IL8
IL1RA
CD23
scaled mean marker exp.
h
tSNE2
tSNE1
i
CD90.2
CD5
CD27
CD3
CD4
CD62L
CD45
CXCR6
gdTCR
KLRG1
GL7
CD44
CD49a
CD49b
CD11b
NK1.1
CXCR5
CD23
B220
IgD
CD19
IgM
CD21_35
CD24
TCRb
CD8
Ly6C
CD93
CD69
CD95
PD-1
CD244
scaled mean marker exp.
j
tSNE2
tSNE1
k
CD279
CD278
CD183
CXCR6
CD134
GITR
HLA
CD137
Tim3
CD11c
CD14
CXCR5
CD19
CD196
CD272
IgM
IgD
CD56
CD161
CD8
CD45RA
CD16
CD28
CD127
CD27
CD3
CD62L
CD4
CD25
CCR7
scaled mean marker exp.

Figure S3

a-e, visualization of HDFC data (chronic HIV dataset). **a,** tSNE visualization of HDFC data colored by Phenograph clustering, color coding according to S3b. **b,** Heatmap of the average expression of each marker split by Phenograph cluster. **c,** UMAP colored according to FlowSOM clustering, color coding according to S3e. **d,** SOM visualization colored by FlowSOM clustering, color coding according to S3e. **e,** Heatmap of the average expression of each marker split by FlowSOM cluster. **f,** tSNE visualization of CyTOF data colored by Phenograph clustering. **g,** Heatmap of the average expression of each marker split by Phenograph cluster, CyTOF data. **h,** tSNE visualization of SpectralFlow data colored by Phenograph clustering. **i,** Heatmap of the average expression of each marker split by Phenograph cluster, SpectralFlow data. **j,** tSNE visualization of CITE-seq data colored by Phenograph clustering. **k,** Heatmap of the average expression of each marker split by Phenograph cluster, CITE-seq data.

Figure S4

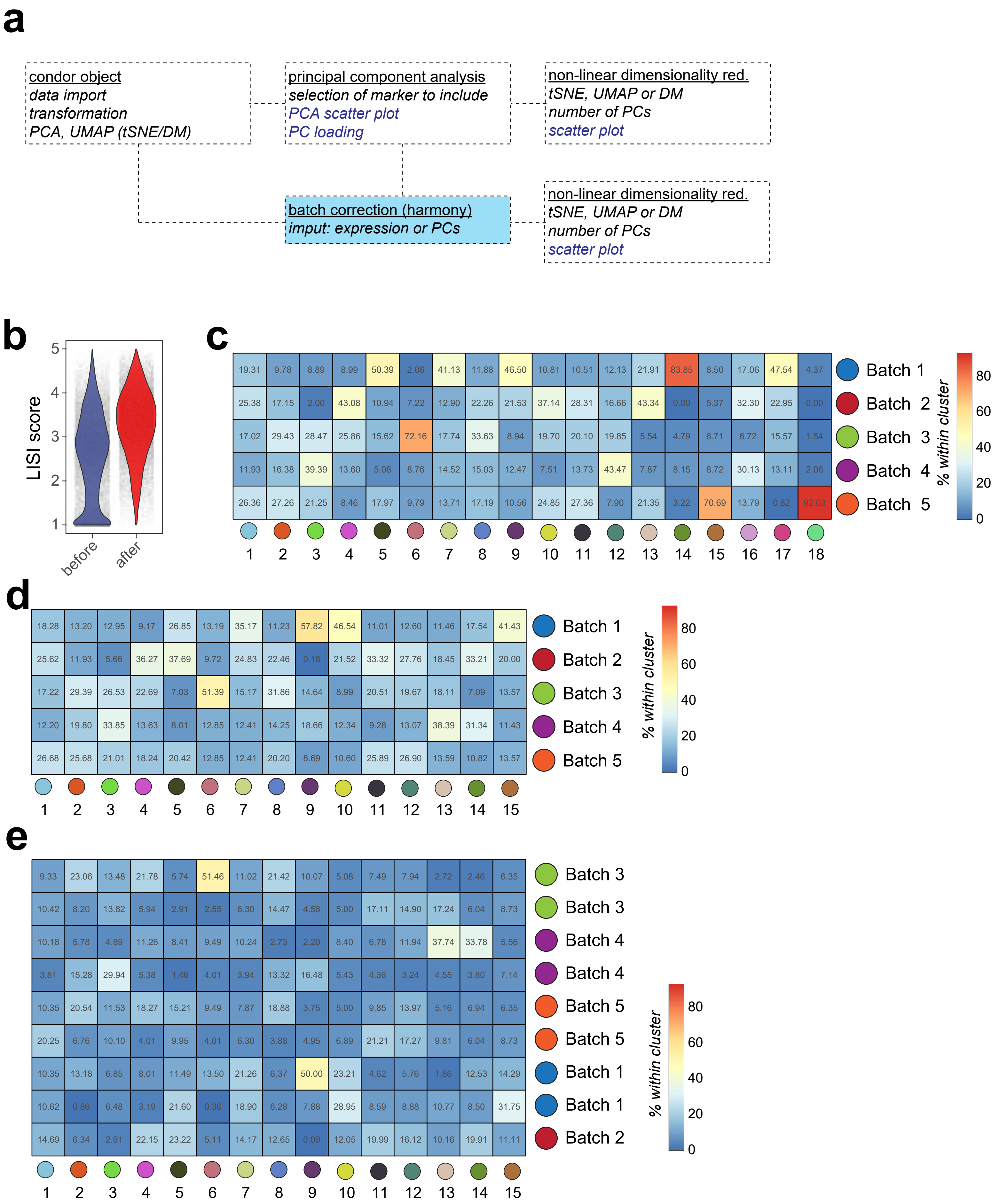
a
condor object
data import
transformation
PCA, UMAP (tSNE/DM)
principal component analysis
selection of marker to include
PCA scatter plot
PC loading
non-linear dimensionality red.
tSNE, UMAP or DM
number of PCs
scatter plot
batch correction (harmony)
imput: expression or PCs
non-linear dimensionality red.
tSNE, UMAP or DM
number of PCs
scatter plot
b
LISI score
before
after
c
Batch 1
Batch 2
Batch 3
Batch 4
Batch 5
% within cluster
d
Batch 1
Batch 2
Batch 3
Batch 4
Batch 5
% within cluster
e
Batch 3
Batch 3
Batch 4
Batch 4
Batch 5
Batch 5
Batch 1
Batch 1
Batch 2
% within cluster

Figure S4

a, Detailed schematic of the batch correction workflow implemented in *cyCONDOR*. **b,** LISI score between batches before and after batch correction**. c,** Confusion matrix of the Phenograph clusters (not corrected data) split by experimental batch. **d,** Confusion matrix of the Phenograph clusters (corrected data) split by experimental batch. **e,** Confusion matrix of the Phenograph clusters (corrected data) split by sample.

Figure S5

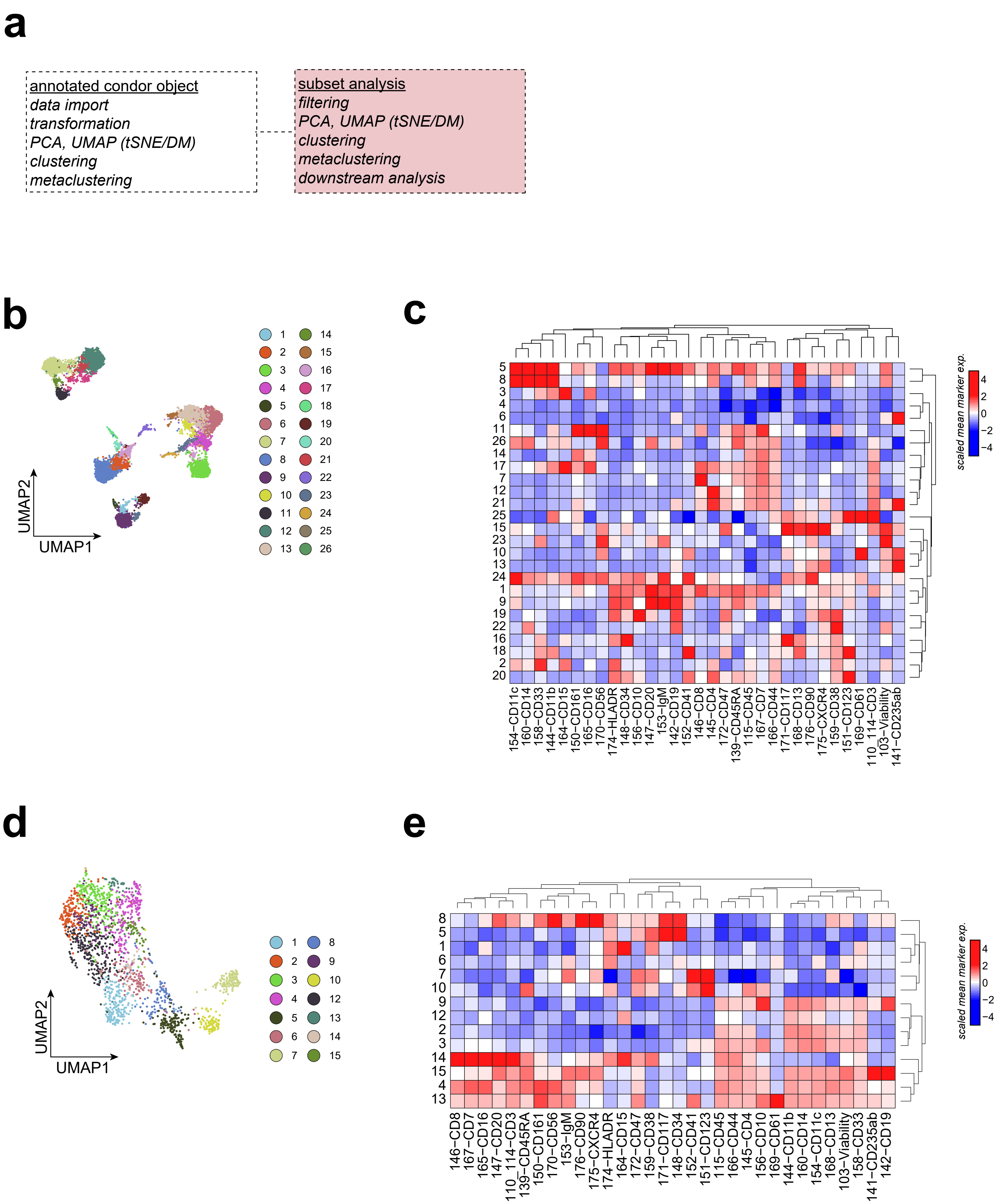
a
annotated condor object
data import
transformation
PCA, UMAP (tSNE/DM)
clustering
metaclustering
subset analysis
filtering
PCA, UMAP (tSNE/DM)
clustering
metaclustering
downstream analysis
b
UMAP2
UMAP1
1 2 3 4 5 6 7 8 9 10 11 12 13 14 15 16 17 18 19 20 21 22 23 24 25 26
c
5 8 3 4 6 11 26 14 17 7 12 21 25 15 23 10 13 24 1 9 19 22 16 18 2 20
154−CD11c 160−CD14 158−CD33 144−CD11b 164−CD15 150−CD161 165−CD16 170−CD56 174−HLADR 148−CD34 156−CD10 147−CD20 153−IgM 142−CD19 152−CD41 146−CD8 145−CD4 172−CD47 139−CD45RA 115−CD45 167−CD7 166−CD44 171−CD117 168−CD13 176−CD90 175−CXCR4 159−CD38 151−CD123 169−CD61 110_114−CD3 103−Viability 141−CD235ab
scaled mean marker exp.
4 2 0 −2 −4
d
UMAP2
UMAP1
1 2 3 4 5 6 7 8 9 10 12 13 14 15
e
8 5 1 6 7 10 9 12 2 3 14 15 4 13
146−CD8 167−CD7 165−CD16 147−CD20 110_114−CD3 139−CD45RA 150−CD161 170−CD56 153−IgM 176−CD90 175−CXCR4 174−HLADR 164−CD15 172−CD47 159−CD38 171−CD117 148−CD34 152−CD41 151−CD123 115−CD45 166−CD44 145−CD4 156−CD10 169−CD61 144−CD11b 160−CD14 154−CD11c 168−CD13 103−Viability 158−CD33 141−CD235ab 142−CD19
scaled mean marker exp.
4 2 0 −2 −4

Figure S5

a, Detailed schematic of the subsetting workflow implemented in *cyCONDOR*. **b,** UMAP of all bone marrow cells colored according to the assigned Phenograph cluster. **c,** Heatmap of the average expression of each marker split by Phenograph cluster. **d,** UMAP of the subsetted dataset colored according to the newly assigned Phenograph cluster. **e,** Heatmap of the average expression of each marker split by Phenograph cluster, subsetted dataset.

Figure S6

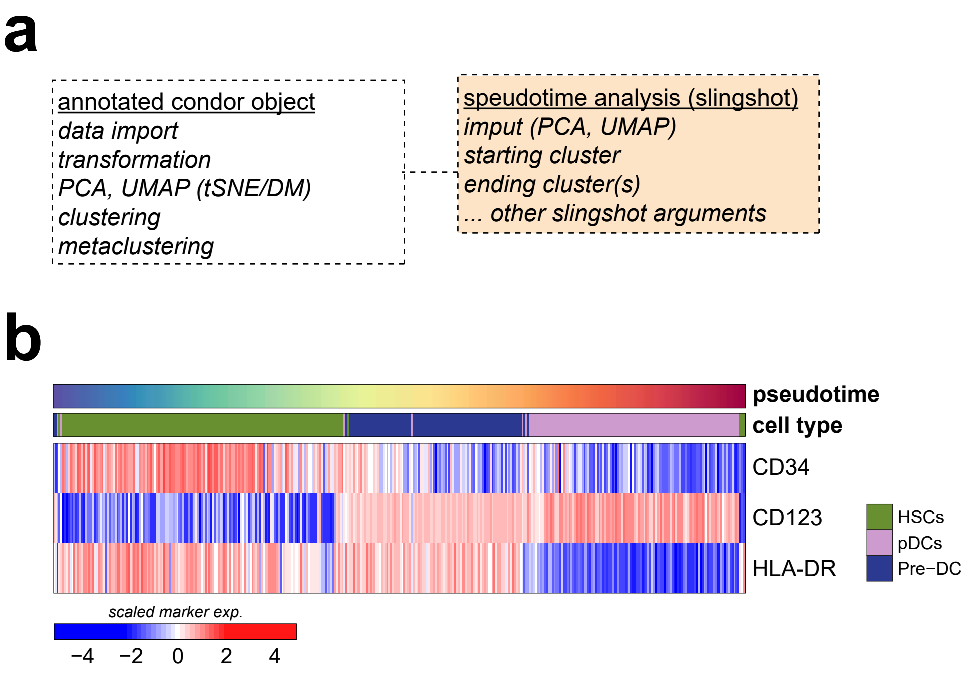
a
annotated condor object
data import
transformation
PCA, UMAP (tSNE/DM)
clustering
metaclustering
speudotime analysis (slingshot)
imput (PCA, UMAP)
starting cluster
ending cluster(s)
... other slingshot arguments
b
pseudotime
cell type
CD34
CD123
HLA-DR
HSCs
pDCs
Pre−DC
scaled marker exp.
−4
−2
0
2
4

Figure S6

a, Detailed schematic of the pseudotime inference workflow implemented in *cyCONDOR*. **b,** Heatmap marker expression in cells belonging to the pDC trajectory ordered according to the inferred pseudotime.

Figure S7

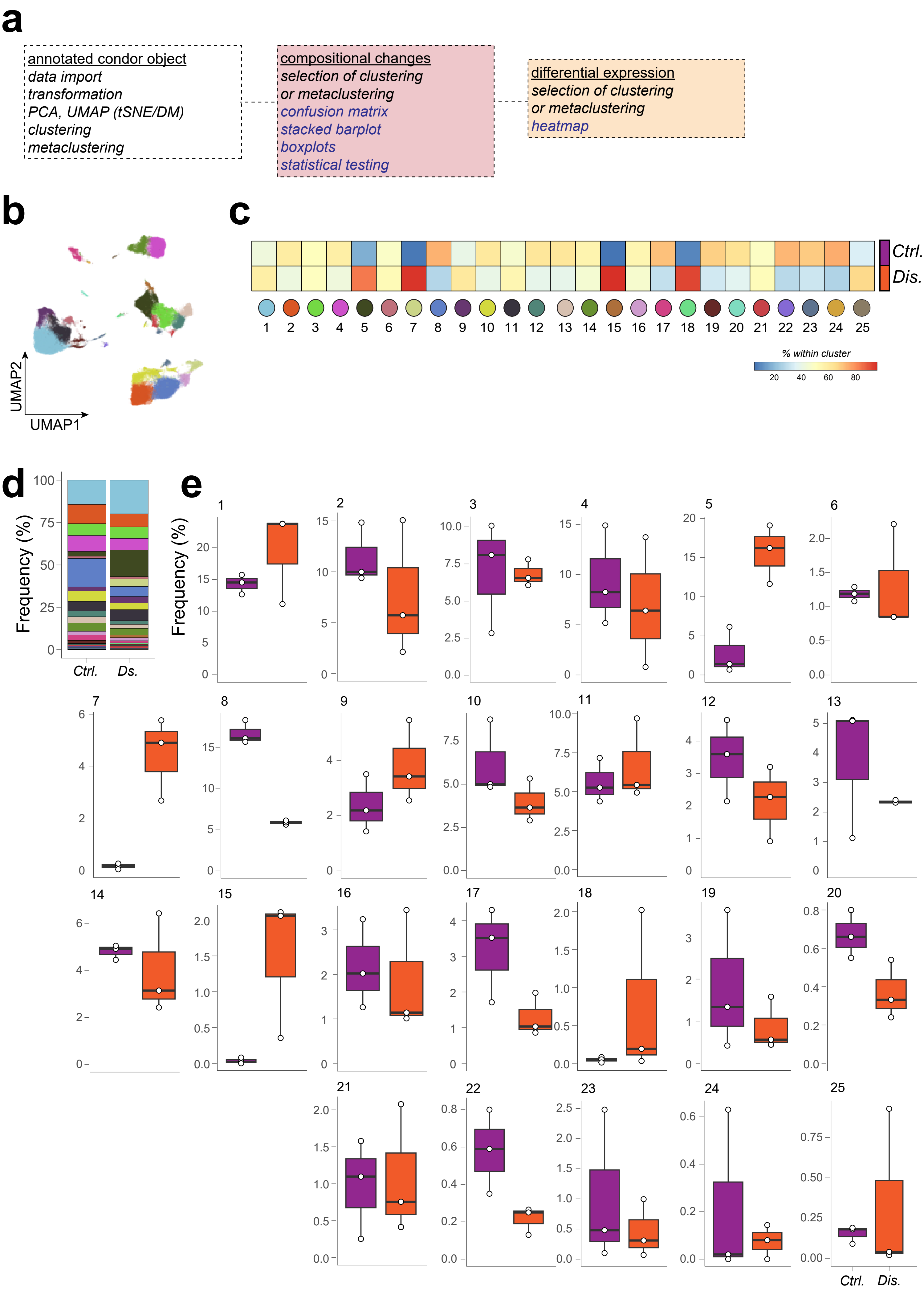
a
annotated condor object
data import
transformation
PCA, UMAP (tSNE/DM)
clustering
metaclustering
compositional changes
selection of clustering
or metaclustering
confusion matrix
stacked barplot
boxplots
statistical testing
differential expression
selection of clustering
or metaclustering
heatmap
b
UMAP2
UMAP1
c
Ctrl.
Dis.
1 2 3 4 5 6 7 8 9 10 11 12 13 14 15 16 17 18 19 20 21 22 23 24 25
% within cluster
20 40 60 80
d
Frequency (%)
Ctrl.
Ds.
e
Frequency (%)
Ctrl.
Dis.

Figure S7

a, Detailed schematic of the differential analysis implemented in the *cyCONDOR* ecosystem. **b,** UMAP colored by Phenograph clustering, color coding shared with S7c. **c,** Confusion matrix of the Phenograph clusters split by experimental group. **d,** Stacked barplot of the Phenograph clusters frequencies split by experimental groups. **e,** Boxplot of the frequency of each Phenograph cluster split by experimental group (Ctrl, n=3; Dis. n=3, n number defines individual donors, Tukey-style boxplot). Statistical significance was calculated with a tow-sided t-test with default settings and bonferroni multiple test correction, * $p < 0.05$, ** $p < 0.01$, *** $p < 0.001$. Source data are provided as a Source Data file.

Figure S8

a

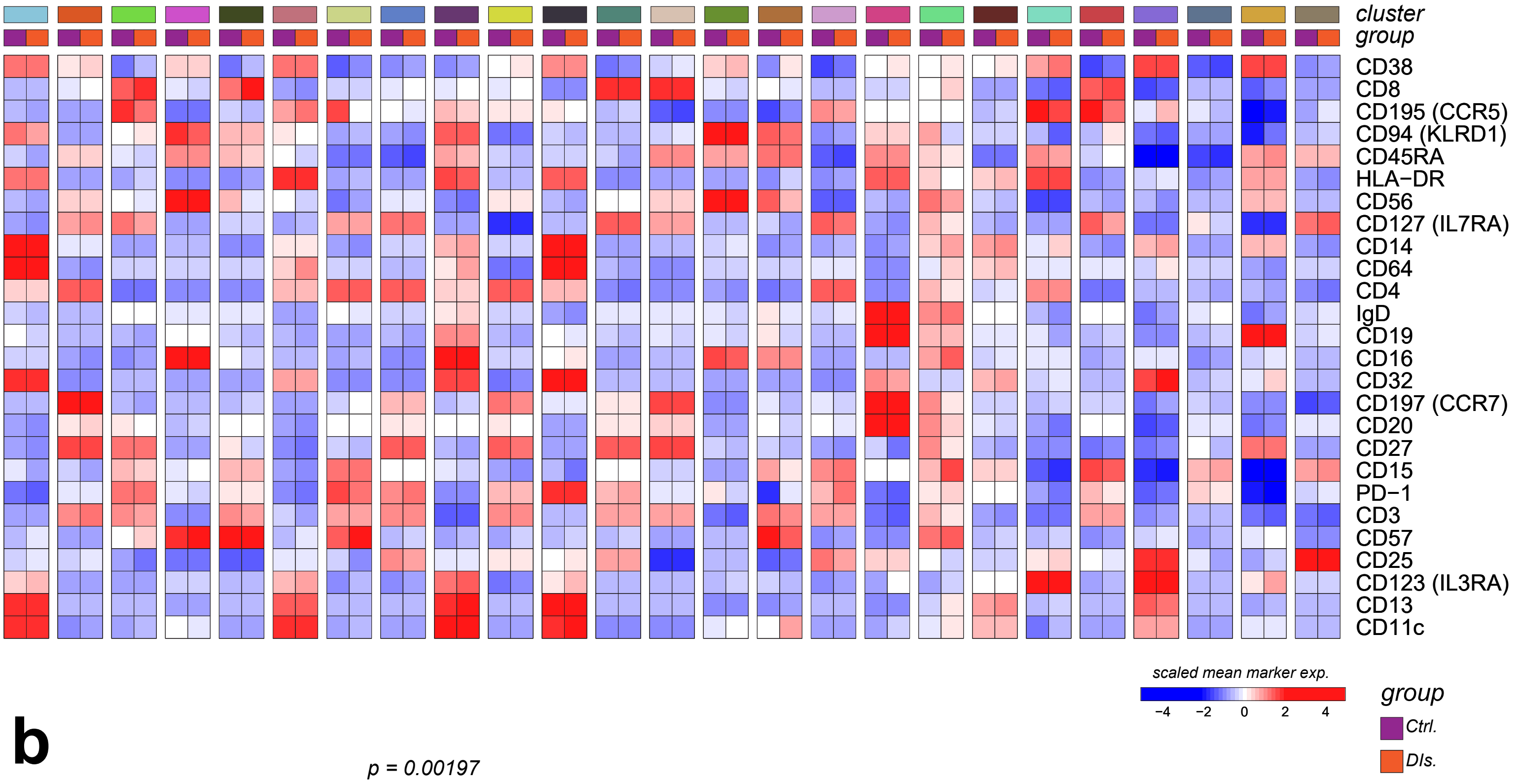
cluster
group
CD38
CD8
CD195 (CCR5)
CD94 (KLRD1)
CD45RA
HLA−DR
CD56
CD127 (IL7RA)
CD14
CD64
CD4
IgD
CD19
CD16
CD32
CD197 (CCR7)
CD20
CD27
CD15
PD−1
CD3
CD57
CD25
CD123 (IL3RA)
CD13
CD11c
scaled mean marker exp.
−4 −2 0 2 4
group
Ctrl.
Dis.

b

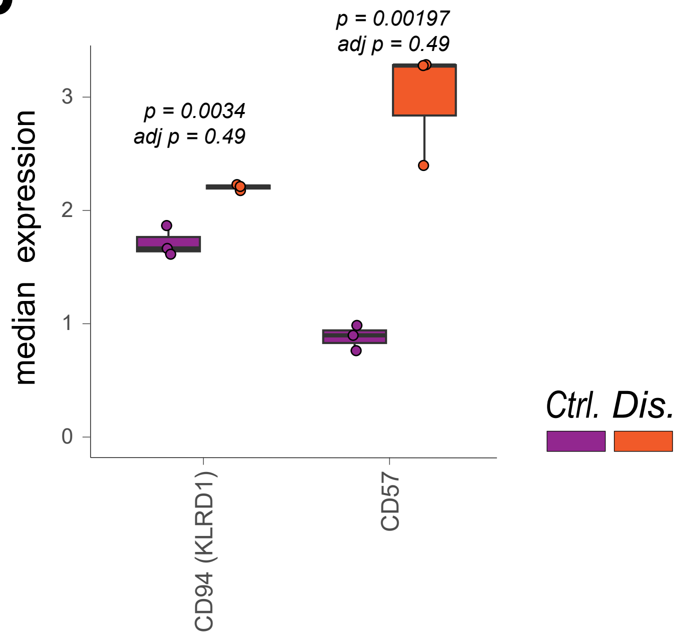
p = 0.00197
adj p = 0.49
p = 0.0034
adj p = 0.49
median expression
0
1
2
3
CD94 (KLRD1)
CD57
Ctrl. Dis.

Figure S8

a, Heatmap of the average expression of each marker split by Phenograph cluster and experimental group. **b,** Boxplot of median marker expression for the two most changed markers in CD8 T cells, p values and FDR corrected p-values are shown. Source data are provided as a Source Data file.

Figure S9

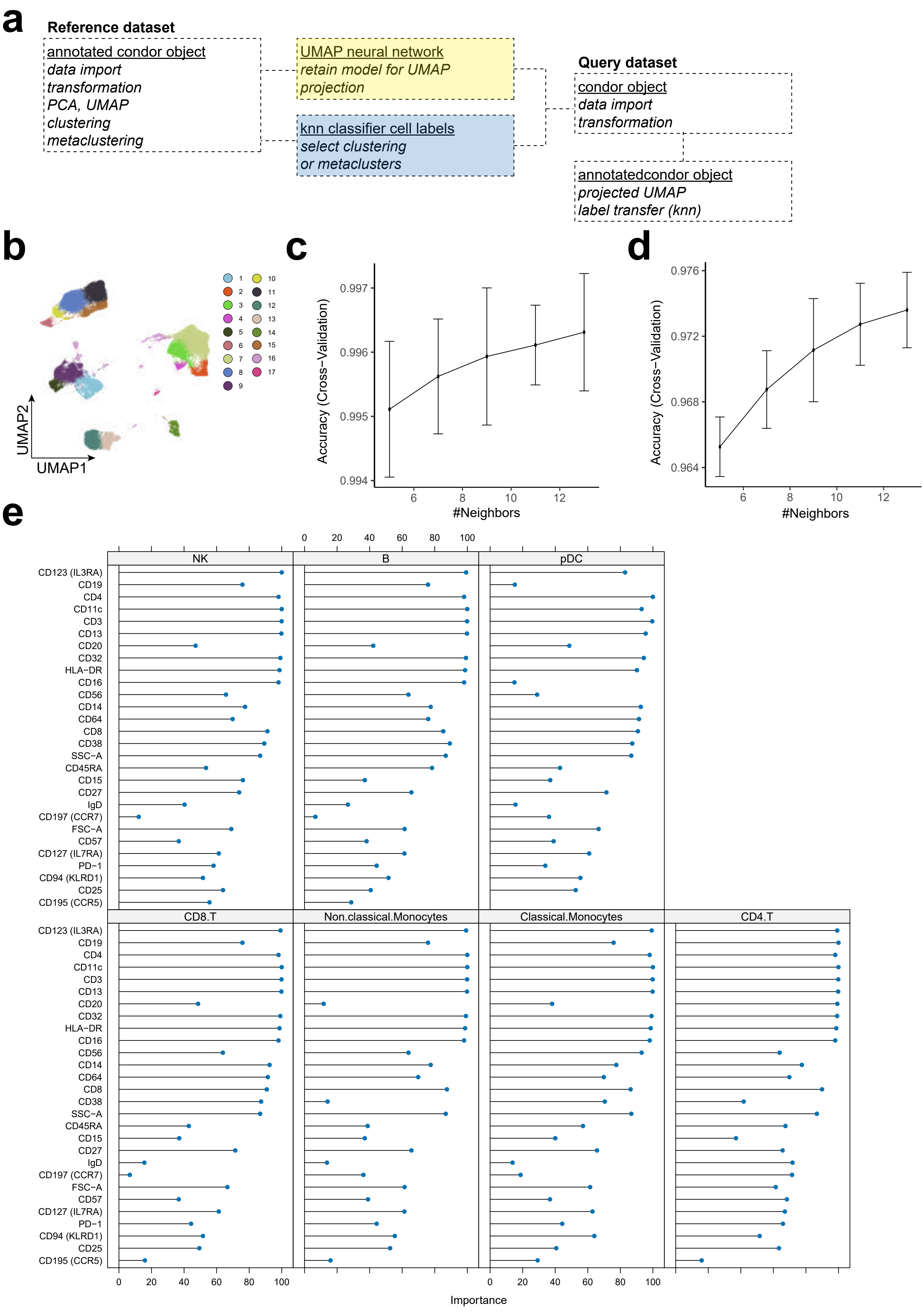
a
Reference dataset
annotated condor object
data import
transformation
PCA, UMAP
clustering
metaclustering
UMAP neural network
retain model for UMAP
projection
knn classifier cell labels
select clustering
or metaclusters
Query dataset
condor object
data import
transformation
annotatedcondor object
projected UMAP
label transfer (knn)
b
UMAP1
UMAP2
1 2 3 4 5 6 7 8 9 10 11 12 13 14 15 16 17
c
Accuracy (Cross−Validation)
#Neighbors
0.994 0.995 0.996 0.997
6 8 10 12
d
Accuracy (Cross−Validation)
#Neighbors
0.964 0.968 0.972 0.976
6 8 10 12
e
NK
B
pDC
CD8.T
Non.classical.Monocytes
Classical.Monocytes
CD4.T
CD123 (IL3RA)
CD19
CD4
CD11c
CD3
CD13
CD20
CD32
HLA−DR
CD16
CD56
CD14
CD64
CD8
CD38
SSC−A
CD45RA
CD15
CD27
IgD
CD197 (CCR7)
FSC−A
CD57
CD127 (IL7RA)
PD−1
CD94 (KLRD1)
CD25
CD195 (CCR5)
0 20 40 60 80 100
Importance

Figure S9

a, Detailed schematic of the data projection workflow implemented in *cyCONDOR*. **b,** UMAP visualization of the training dataset colored according to the assigned Phenograph clustering. **c,** Accuracy of the kNN model trained to predict annotated cell labels across different numbers of neighbors used for model optimization. **d,** Accuracy of the kNN model trained to predict Phenograph clusters across different numbers of neighbors used for model optimization. **e,** Importance score for the assignment of each cell label for individual annotated cell labels**.**

Figure S10

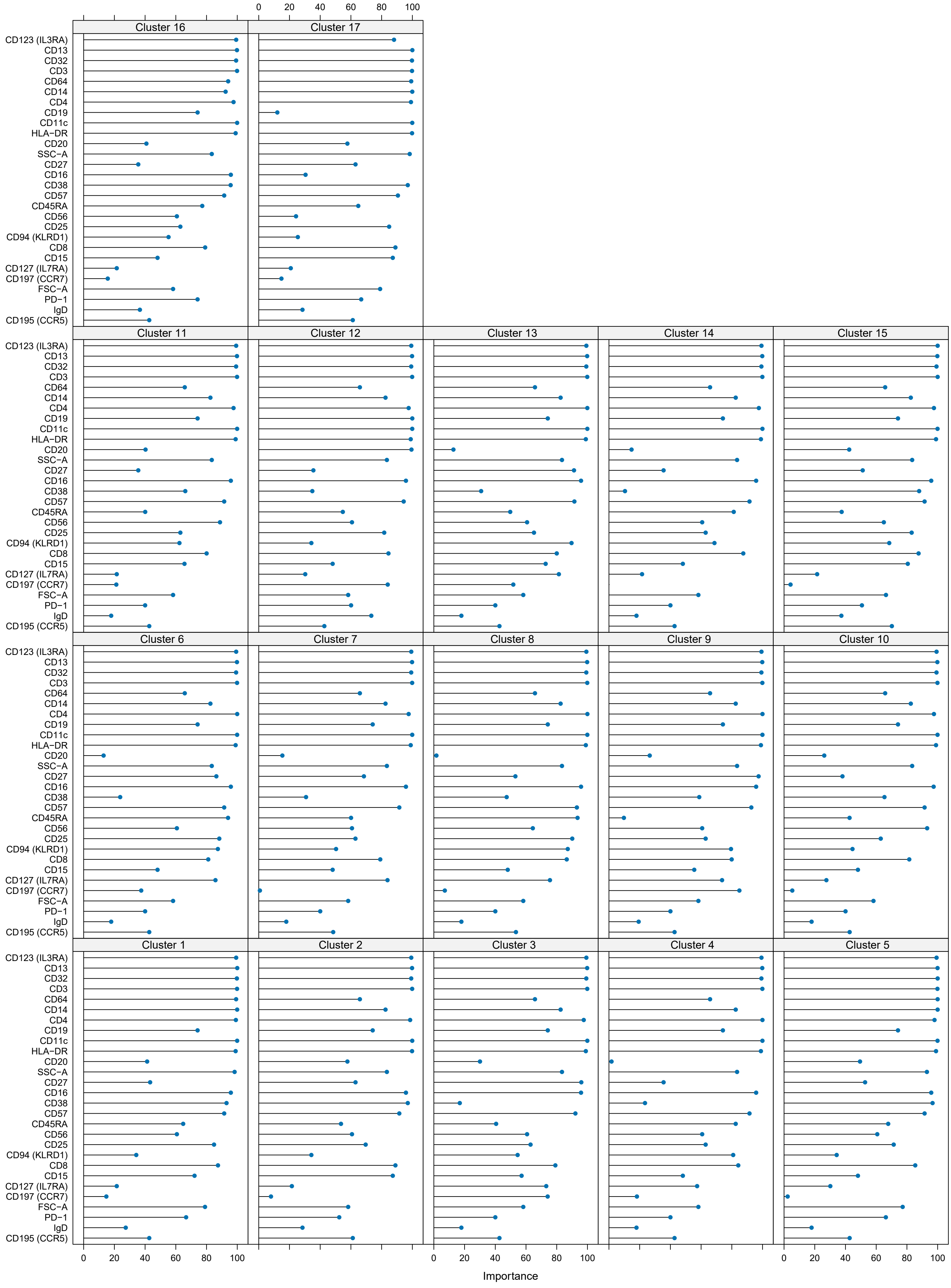

Cluster 16
Cluster 17
Cluster 11
Cluster 12
Cluster 13
Cluster 14
Cluster 15
Cluster 6
Cluster 7
Cluster 8
Cluster 9
Cluster 10
Cluster 1
Cluster 2
Cluster 3
Cluster 4
Cluster 5
CD123 (IL3RA)
CD13
CD32
CD3
CD64
CD14
CD4
CD19
CD11c
HLA−DR
CD20
SSC−A
CD27
CD16
CD38
CD57
CD45RA
CD56
CD25
CD94 (KLRD1)
CD8
CD15
CD127 (IL7RA)
CD197 (CCR7)
FSC−A
PD−1
IgD
CD195 (CCR5)
0
20
40
60
80
100
Importance

Figure S10

Importance score for the assignment of each cell label for individual Phenograph clusters**.**

Figure S11

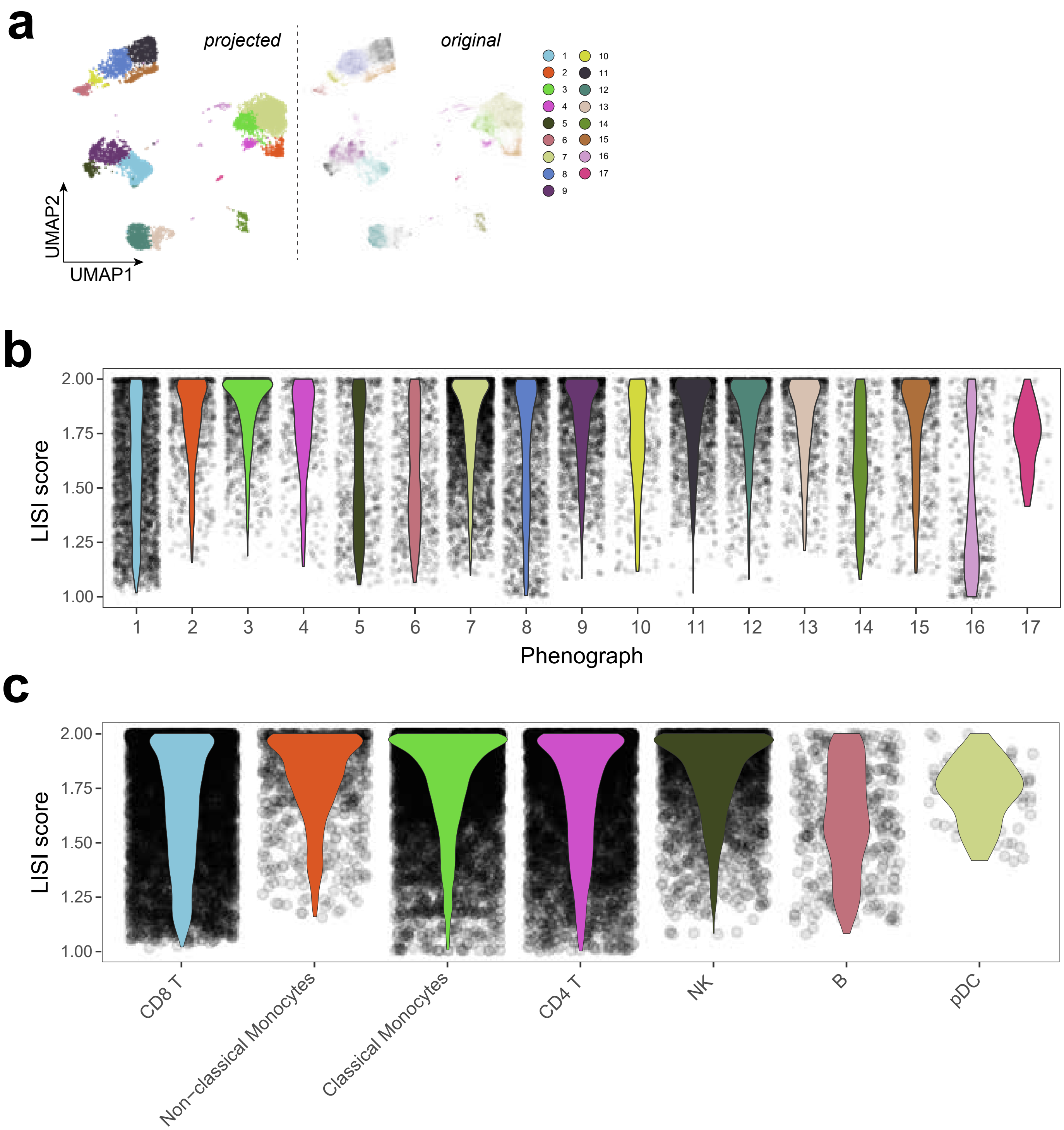
a
projected
original
1
2
3
4
5
6
7
8
9
10
11
12
13
14
15
16
17
UMAP2
UMAP1
b
LISI score
2.00
1.75
1.50
1.25
1.00
1 2 3 4 5 6 7 8 9 10 11 12 13 14 15 16 17
Phenograph
c
LISI score
2.00
1.75
1.50
1.25
1.00
CD8 T
Non−classical Monocytes
Classical Monocytes
CD4 T
NK
B
pDC

Figure S11

a, UMAP visualization of the projected data colored according to the predicted Phenograph cluster. **b,** LISI score calculated between training data and projected data split by Phenograph cluster. **c,** LISI score split by assigned cell type.

Figure S12

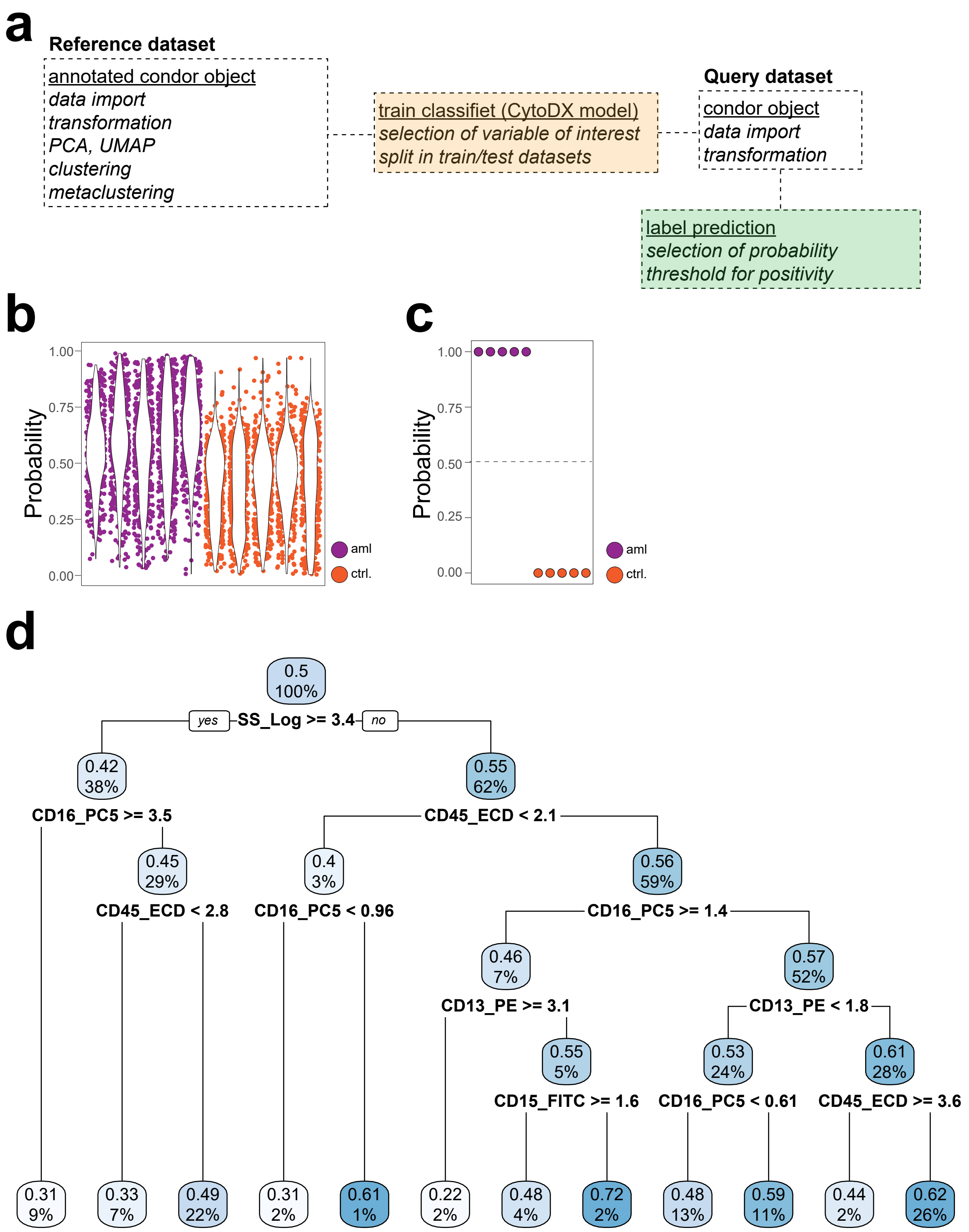
a
Reference dataset
annotated condor object
data import
transformation
PCA, UMAP
clustering
metaclustering
train classifiet (CytoDX model)
selection of variable of interest
split in train/test datasets
Query dataset
condor object
data import
transformation
label prediction
selection of probability
threshold for positivity
b
Probability
1.00
0.75
0.50
0.25
0.00
aml
ctrl.
c
Probability
1.00
0.75
0.50
0.25
0.00
aml
ctrl.
d
0.5 100%
yes
SS_Log >= 3.4
no
0.42 38%
0.55 62%
CD16_PC5 >= 3.5
CD45_ECD < 2.1
0.45 29%
0.4 3%
0.56 59%
CD45_ECD < 2.8
CD16_PC5 < 0.96
CD16_PC5 >= 1.4
0.46 7%
0.57 52%
CD13_PE >= 3.1
CD13_PE < 1.8
0.55 5%
0.53 24%
0.61 28%
CD15_FITC >= 1.6
CD16_PC5 < 0.61
CD45_ECD >= 3.6
0.31 9%
0.33 7%
0.49 22%
0.31 2%
0.61 1%
0.22 2%
0.48 4%
0.72 2%
0.48 13%
0.59 11%
0.44 2%
0.62 26%

Figure S12

a, Detailed schematic of the clinical classifier workflow implemented in *cyCONDOR*. **b,** Single-cell level probability for the training dataset split by sample and colored by experimental group. **c,** Sample level probability for the training dataset split by sample and colored by experimental group. **d,** A decision tree classifies cells as aml. Each branch of the tree represents a specific characteristic, and the value at each node shows the likelihood of aml association for that group of cells. The rules at each branch further divide the cell population into more refined subgroups based on additional characteristics.
