# Supplementary material for: Unveiling the power of high-dimensional cytometry data with cyCONDOR
Source: Nat Commun. 2024 Dec 19;15:10702. doi: 10.1038/s41467-024-55179-w (PMC11659560; doi:10.1038/s41467-024-55179-w)
Supplement: Supplementary file 2 — Description of Additional Supplementary Files [file 41467_2024_55179_MOESM2_ESM.pdf]

Description of Additional Supplementary Files:

Supplementary Data 1: Prepare data for cyCONDOR analysis

Supplementary Data 2: CD19 expression across clusters

Supplementary Data 3: Harmony batch correction for Spectral Flow, cyTOF and CITE-seq data

Supplementary Data 4: Harmony batch correction with simulated batch in cyTOF data

Supplementary Data 5: DA with cyCONDOR across clusters

Supplementary Data 6: DA with cyCONDOR across cell types

Supplementary Data 7: DA with diffcyt across clusters

Supplementary Data 8: DA with diffcyt across cell types

Supplementary Data 9: DE with diffcyt across cell types

Supplementary Data 10: Comparison between paired CITE-seq and HDC data

Supplementary Data 11: Code and output Figure 2 - HDC

Supplementary Data 12: Code and output Figure 2 - cyTOF

Supplementary Data 13: Code and output Figure 2 - SpectralFlow

Supplementary Data 14: Code and output Figure 2 - CITE-seq

Supplementary Data 15: Code and output Figure 3

Supplementary Data 16: Code and output Figure 4

Supplementary Data 17: Code and output Figure 5

Supplementary Data 18: Code and output Figure 6

Supplementary Data 19: Code and output Figure 7 - test data

Supplementary Data 20: Code and output Figure 7 - permutation test
