# Supplementary material for: Unveiling the power of high-dimensional cytometry data with cyCONDOR
Source: Nat Commun. 2024 Dec 19;15:10702. doi: 10.1038/s41467-024-55179-w (PMC11659560; doi:10.1038/s41467-024-55179-w)
Supplement: Supplementary file 3 — Supplementary Data 1 [file 41467_2024_55179_MOESM3_ESM.html]

Supplementary Data 1: Prepare data for cyCONDOR analysis


# Supplementary Data 1: Prepare data for cyCONDOR analysis

We provide here a detailed step-by-step guideline on how to export
compensated `.fcs` files from `FlowJo v10` and
prepare the metadata annotation table for `cyCONDOR`
analysis.

## Step 1

Within the `FlowJo workspace`, you can adjust compensation
as needed and optionally apply a `basic gating hierarchy` to
your data. This is recommended to remove debris and unwanted cells,
reducing the size of your final dataset and improving computational
efficiency. For instance, during immune cell analysis, consider gating
on `CD45+` cells for `cyCONDOR` analysis.

## Step 2

Right-click in the gate you want to export and select
`Export/Concatenate Populations`

*Tip:* If you want to export this gate for all the samples in
the workspace, before this step right-click on the desired gate and
select `Select Equivalent Nodes` to select the same gate in
all samples.

*Exemplary `FlowJo workspace` (left) and exemplary
option window shown after right-clicking on the desired gate*

## Step 3

Now, choose the format for your exported data. `FCS3` is
the recommended format. Additionally, select the destination folder
where you want to save the file. You have two options for selecting
which channels to export:

- **Export all compensated values:** This option is
  selected in the image below and will export all channels that have been
  compensated.
- **Manually select compensated channels:** This option
  allows you to choose specific compensated channels to include in the
  exported `.fcs` file.

*Note:* If data were not compensated in FlowJo, for example
cyTOF data or BD S8 unmixed data, export `uncompensated`
values.

*`Population Export or Concatenate` window*

## Step 4

Click on `Export`. Now all selected files will be exported
in the selected folder. As input for `cyCONDOR` the
`data_path` should only contain the `.fcs` files
intended for the analysis.

*Exemplary folder with exported `.fcs` files*

## Step 5

The `annotation table`, used to load `.fcs`
files, should have the file names of all `.fcs` files in the
first column. Additional columns can be included to store any relevant
metadata for your analysis. There are no restrictions on the type of
information you can include in these metadata columns. We advise to save
the annotation table in a separate folder and save it as comma separated
`.csv` file. An example of annotation table is shown
below:

*Exemplary metadata table visualized in Excel (left) and Notepad
(right)*
