# Supplementary material for: Unveiling the power of high-dimensional cytometry data with cyCONDOR
Source: Nat Commun. 2024 Dec 19;15:10702. doi: 10.1038/s41467-024-55179-w (PMC11659560; doi:10.1038/s41467-024-55179-w)
Supplement: Supplementary file 4 — Supplementary Data 2 [file 41467_2024_55179_MOESM4_ESM.pdf]

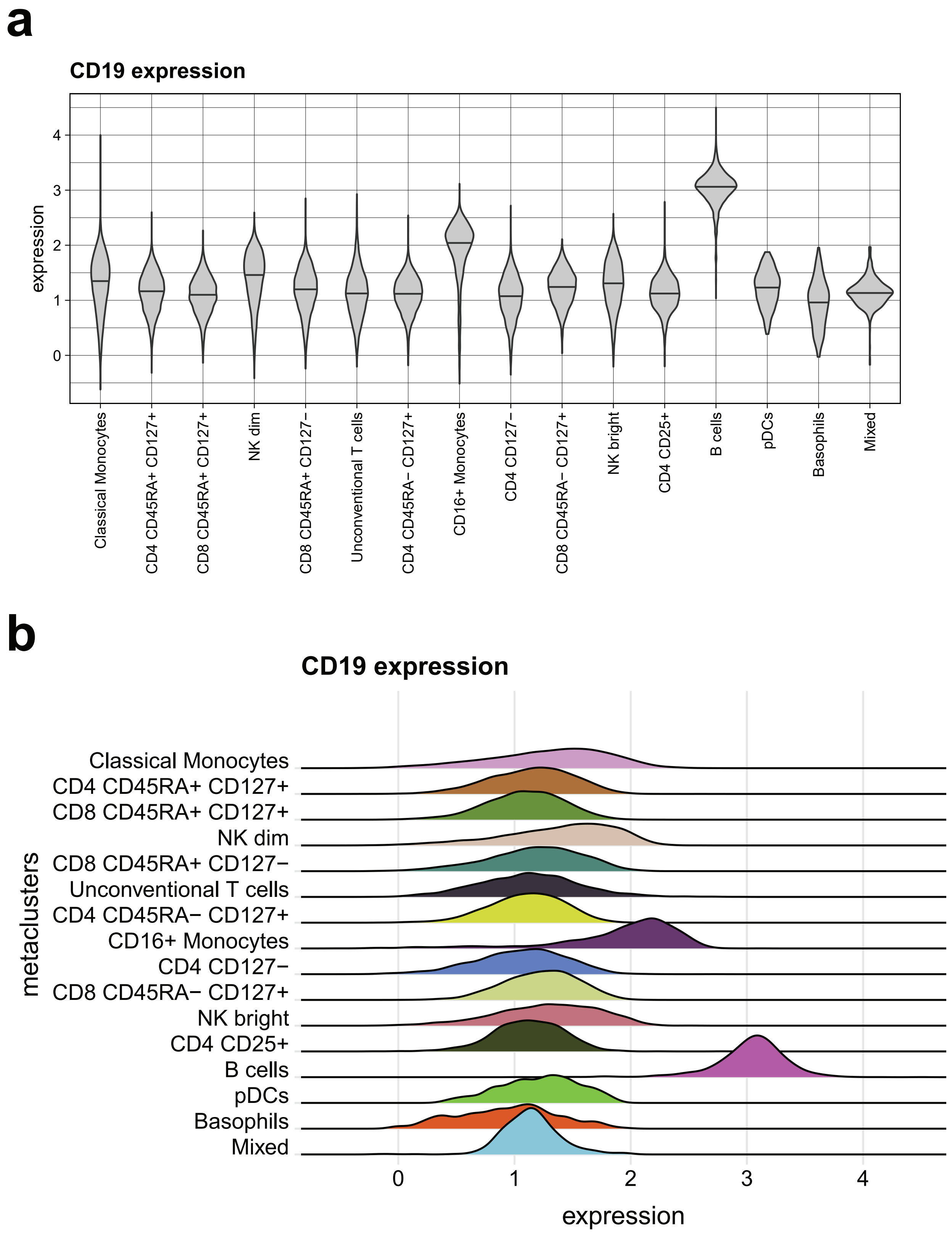


**Supplementary Data 2:** Violin plot (**a**) and ridgeline plot (**b**) of transformed expression values showing the highest expression of CD19 in B cells, nevertheless the plot also highlights an increased "expression" of CD19 also in other cell types expressing high levels of CD16. Despite compensation of the fluorescence signal this is the result of data spread between two channels with high spectral overlap, an effect not possible to fully remove in conventional flow cytometry.
