# Supplementary material for: Unveiling the power of high-dimensional cytometry data with cyCONDOR
Source: Nat Commun. 2024 Dec 19;15:10702. doi: 10.1038/s41467-024-55179-w (PMC11659560; doi:10.1038/s41467-024-55179-w)
Supplement: Supplementary file 5 — Supplementary Data 3 [file 41467_2024_55179_MOESM5_ESM.pdf]

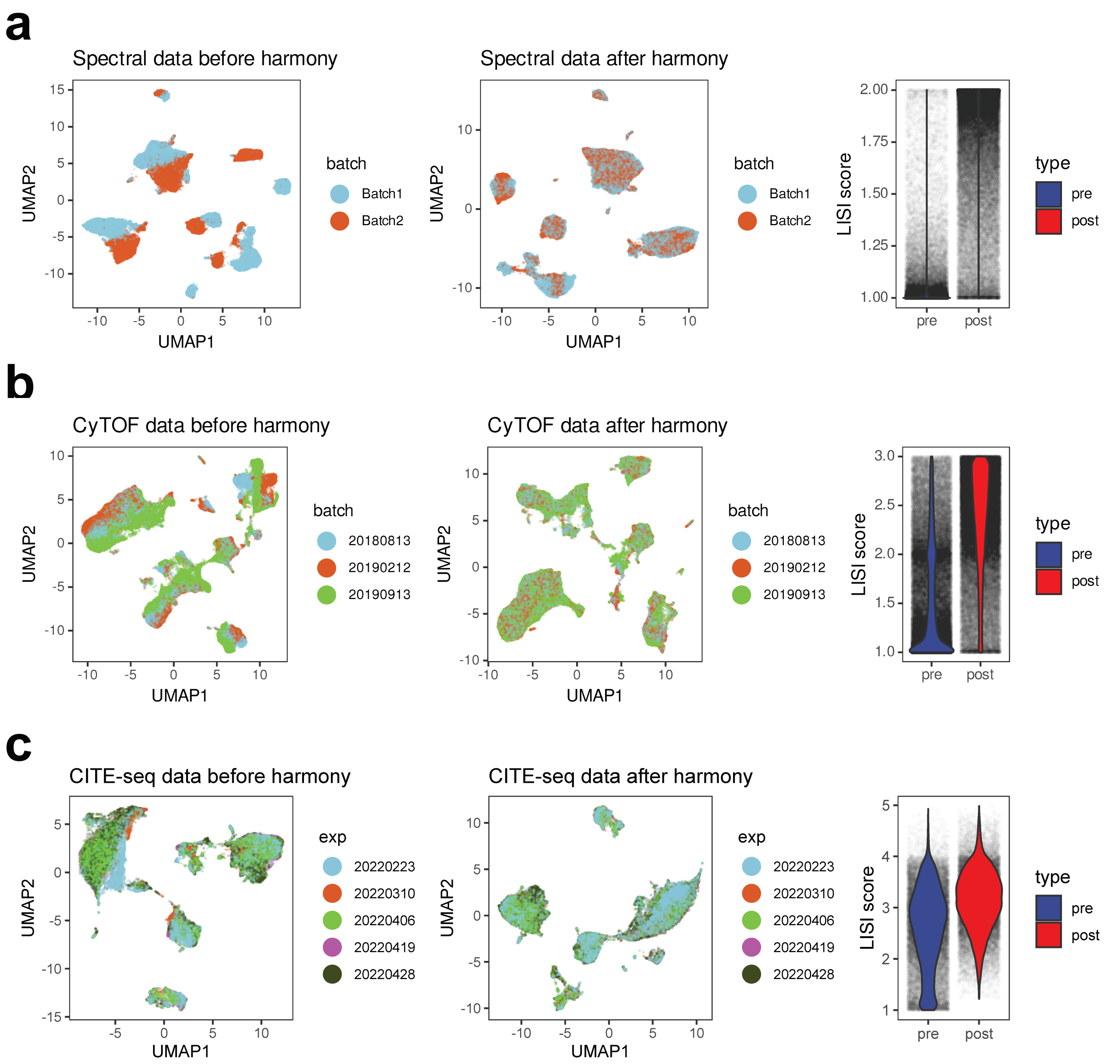

Supplementary Data 3: Validation of harmony batch correction for Spectral Flow,, cyTOF and CITE-seq data. **a:** Harmony batch correction performed on Spectral Flow data, this dataset was kindly provided by Dr. Ogischi and was previously published for the validation of the iMUBAC tool (Ogishi et al., 2021), We show a drastic improvement of the LISI score after batch correction with harmony. **b:** Similarly the cyTOF dataset used to validate the usage of harmony with cyTOF data was also provided by Dr. Ogischi and was previously published for the validation of the iMUBAC tool (Ogishi et al., 2021). Also here we could show that harmony batch correction is able to reduce the technical batch in the dataset showing also here a strong improvement of the LISI score. **c:** Harmony batch correction was validated on CITE-seq data using an unpublished in-house dataset, a total of nine samples measured at five dates following the BD Rhapsody AbSeq protocol. Also in this case harmony correction was able to reduce the technical variation in the dataset substantially improving the LISI score.
