# Supplementary material for: Unveiling the power of high-dimensional cytometry data with cyCONDOR
Source: Nat Commun. 2024 Dec 19;15:10702. doi: 10.1038/s41467-024-55179-w (PMC11659560; doi:10.1038/s41467-024-55179-w)
Supplement: Supplementary file 6 — Supplementary Data 4 [file 41467_2024_55179_MOESM6_ESM.pdf]

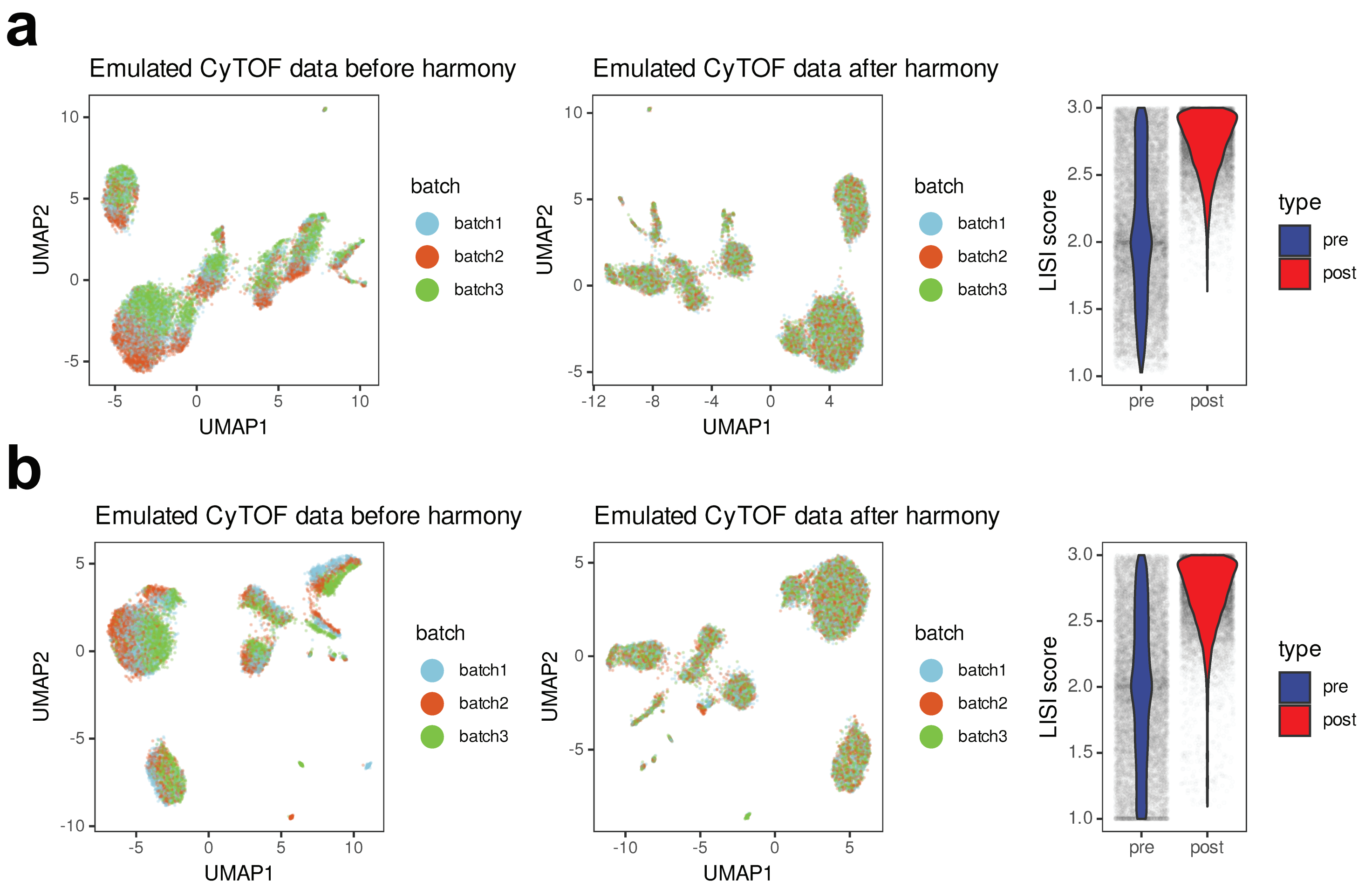

Supplementary Data 4: Validation of harmony batch correction for cyTOF data using generated batch effect. In this validation one sample from a previously published cyTOF dataset (Krieg et al., 2018). We generated both a global (a) and local batch effect (b) using the cytomulate tool (Yang et al., 2023). We report here UMAP dimensionality reduction before and after harmony integration coloured by simulated batch. We also report the LISI score before and after batch correction showing a good integration of the dataset with harmony.
