# Supplementary material for: Unveiling the power of high-dimensional cytometry data with cyCONDOR
Source: Nat Commun. 2024 Dec 19;15:10702. doi: 10.1038/s41467-024-55179-w (PMC11659560; doi:10.1038/s41467-024-55179-w)
Supplement: Supplementary file 12 — Supplementary Data 10 [file 41467_2024_55179_MOESM12_ESM.pdf]

a

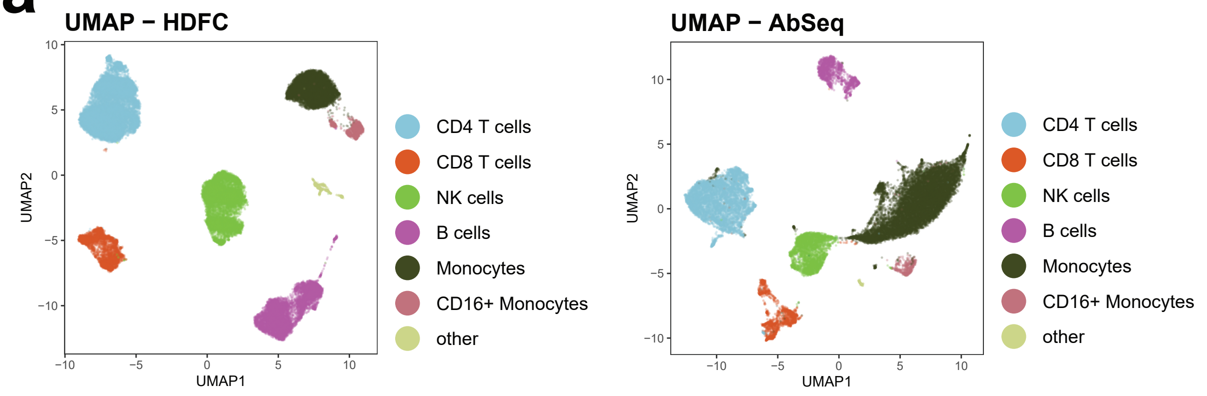

b

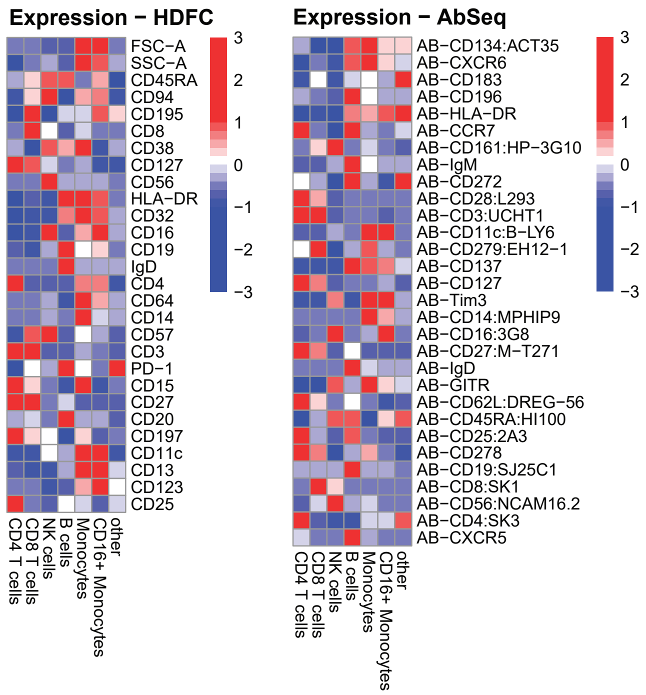

c

```
freq_fc <- getTable(fcd = condor_fc,
                    output_type = "frequency",#alternative "counts"
                    cluster_slot = "Phenograph_pca_norm_k_60",
                    cluster_var = "metaclusters",
                    group_var = "ID",
                    numeric = F)

knitr::kable(freq_fc, row.names = FALSE)
```

group_var	B cells	CD16+ Monocytes	CD4 T cells	CD8 T cells	Monocytes	NK cells	other
193166520_193364_C8	7.46	0.36	70.10	7.30	7.58	6.68	0.52
193202929_193363_D12	43.08	2.08	15.12	4.72	11.74	20.10	3.16
193206554_207046_B4	5.30	2.94	36.54	5.22	32.32	16.20	1.48
193212837_193364_F12	23.34	2.46	25.28	2.88	9.62	35.66	0.76
193223821_193363_E2	7.70	1.46	51.08	15.94	7.06	16.38	0.38
193224956_193363_A4	11.58	3.78	41.56	13.72	6.96	21.62	0.78
195373912_193363_F12	23.14	5.80	11.62	12.36	16.78	28.24	2.06
195376956_193363_F1	28.40	0.72	32.88	7.94	6.98	22.32	0.76
195377061_193363_G8	16.82	5.38	13.62	3.64	40.38	18.58	1.58

```
freq_abseq <- getTable(fcd = condor_abseq,
                       output_type = "frequency",#alternative "counts"
                       cluster_slot = "Phenograph_pca_norm_k_30",
                       cluster_var = "metaclusters",
                       group_var = "ID",
                       numeric = F)

knitr::kable(freq_abseq, row.names = FALSE)
```

group_var	B cells	CD16+ Monocytes	CD4 T cells	CD8 T cells	Monocytes	NK cells	other
193166520_193364_C8	3.508772	1.0121457	53.542510	4.3859649	32.96221	4.251012	0.3373819
193202929_193363_D12	12.504597	2.1331372	7.760206	5.1489518	58.07282	13.497609	0.8826775
193206554_207046_B4	2.843045	0.8458647	32.706767	6.7199248	42.03477	14.661654	0.1879699
193212837_193364_F12	1.776199	2.0870337	2.064831	0.5106572	88.63233	4.551510	0.3774423
193223821_193363_E2	4.411434	1.6205267	36.191762	13.4593743	32.63561	11.186136	0.4951609
193224956_193363_A4	3.991292	3.0116110	32.692308	10.8853411	31.34978	17.706822	0.3628447
195373912_193363_F12	14.529915	1.8518519	10.541310	12.6780627	36.60969	23.646724	0.1424501
195376956_193363_F1	18.446985	0.3153331	30.114308	6.5825778	24.20181	19.392984	0.9459992
195377061_193363_G8	9.026339	3.0186446	11.985795	3.2849956	56.43682	15.921870	0.3255401

d

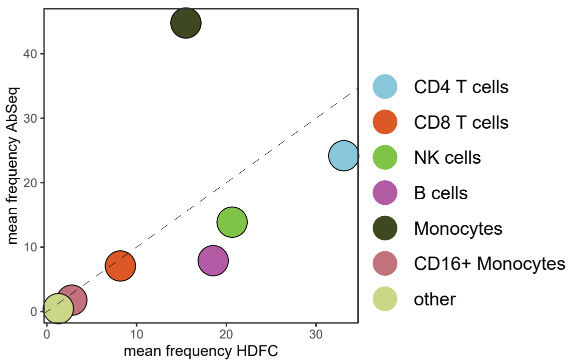

Supplementary Data 10: Exemplification of the cyCONDOR capabilities in the comparison between samples measured with multiple modalities. In this example the same samples were measured with both HDFC and AbSeq (CITE-seq). Thanks to the unified ecosystem provided by cyCONDOR is possible to easily compare the results of the two modalities. After data loading, batch correction, dimensionality reduction and clustering each dataset was annotated. **a:** UMAP dimensionality reduction of both dataset (HDFC left and AbSeq right) coloured by cell type. At this level is already possible to notice how the Monocytes cluster appears to be larger in AbSeq data compared to HDFC. **b:** Heatmap visualisation of marker expression: with a simple built-in cyCONDOR function is possible to plot marker expression of both modalities, here we can see a strong agreement between marker expression in the two modalities. **c:** From each condor object simple tabular summary results can be exported with cyCONDOR built-in function. Here with a single function (getTable) we export the cellular frequencies for each modality, those tables can be easily used to directly compare the result. **d:** Dotplot showing the correlation between the mean frequencies measured by HDFC and those recorded by AbSeq. Interestingly there is an increased frequency of monocytes and a decreased frequency of lymphocytes (B cells, NK cells CD4 T cells) in AbSeq data. This phenomenon can be explained by that in this dataset the cell calling of the cells is based on mRNA content. Due to the lower mRNA content of lymphocytes and the relatively bad quality of the sample (long-term storage) a loss of lymphocytes in favour of monocytes can be observed. This type of comparative analysis can be easily performed in cyCONDOR without the need of further tools.
