# Supplementary material for: Unveiling the power of high-dimensional cytometry data with cyCONDOR
Source: Nat Commun. 2024 Dec 19;15:10702. doi: 10.1038/s41467-024-55179-w (PMC11659560; doi:10.1038/s41467-024-55179-w)
Supplement: Supplementary file 13 — Supplementary Data 11 [file 41467_2024_55179_MOESM13_ESM.html]

Supplementary Data 11: reproducibility data for Figure 2 - HDFC example dataset


# Supplementary Data 11: reproducibility data for Figure 2 - HDFC example dataset

# Loading required packages

```
library(cyCONDOR, quietly = TRUE)
library(ggplot2)
library(ggsci)
library(dplyr)
library(ggpubr)
library(ggrastr)
library(RColorBrewer)
```

# Loading the data

```
condor <- prep_fcd(data_path  = "./data/FC/", 
                    max_cell = 10000, 
                    useCSV = FALSE, 
                    transformation = "auto_logi", 
                    remove_param = c("FSC-H", "SSC-H", "FSC-W", "SSC-W", "Time", "live_dead"), 
                    anno_table = "./data/FC_metadata.csv", 
                    filename_col = "filename",
                    seed = 91, 
                    verbose = TRUE)
#> [1] "Start reading the data"
#> [1] "Loading file 1 out of 6"
#> [1] "Loading file 2 out of 6"
#> [1] "Loading file 3 out of 6"
#> [1] "Loading file 4 out of 6"
#> [1] "Loading file 5 out of 6"
#> [1] "Loading file 6 out of 6"
#> [1] "Start transforming the data"
#> [1] "FSC-A w= 0 t= 189452.890625"
#> [1] "SSC-A w= 0 t= 159321.1875"
#> [1] "CD38 w= 1.08204990999969 t= 14864.46875"
#> [1] "CD8 w= 1.39647877544147 t= 12298.8408203125"
#> [1] "CD195 (CCR5) w= 1.47449646804538 t= 9324.6044921875"
#> [1] "CD94 (KLRD1) w= 1.08644854094052 t= 67681.4140625"
#> [1] "CD45RA w= 0.624871054871812 t= 188189.21875"
#> [1] "HLA-DR w= 0.929461421442766 t= 47207.3984375"
#> [1] "CD56 w= 1.06132042573662 t= 40519.19921875"
#> [1] "CD127 (IL7RA) w= 1.57275952764248 t= 5211.52490234375"
#> [1] "CD14 w= 1.20279949135721 t= 20560.888671875"
#> [1] "CD64 w= 0.945532824421731 t= 339462.875"
#> [1] "CD4 w= 1.00703642192255 t= 95269.0078125"
#> [1] "IgD w= 1.02627346790408 t= 77893.9609375"
#> [1] "CD19 w= 0.87097003245726 t= 196933.3125"
#> [1] "CD16 w= 0.834816694638715 t= 265510.25"
#> [1] "CD32 w= 0.77458993281154 t= 137295.5625"
#> [1] "CD197 (CCR7) w= 1.06501374238218 t= 28634.5234375"
#> [1] "CD20 w= 1.15174790062917 t= 42374.49609375"
#> [1] "CD27 w= 1.25795116181412 t= 27285.08984375"
#> [1] "CD15 w= 1.29238880617719 t= 52487.171875"
#> [1] "PD-1 w= 1.91214147798757 t= 3227.24658203125"
#> [1] "CD3 w= 1.22361754479372 t= 36392.27734375"
#> [1] "CD57 w= 0.392745042488233 t= 316672.84375"
#> [1] "CD25 w= 1.01070056777946 t= 21352.48828125"
#> [1] "CD123 (IL3RA) w= 1.12760875584084 t= 66552.2265625"
#> [1] "CD13 w= 1.06898469845033 t= 101909.6875"
#> [1] "CD11c w= 1.00050229943375 t= 50178.70703125"
```

```
class(condor)
#> [1] "flow_cytometry_dataframe"
```

# Pseudobulk PCA

```
pb_PCA <- runPCA_pseudobulk(condor)
```

```
ggplot(pb_PCA$pca, aes(x = PC1, y = PC2, color = group)) +
  geom_point(size = 7) +
  scale_color_manual(values = c("#BEBEBE", "#CE2827")) +
  theme_bw() + theme(aspect.ratio = 1) + ggtitle("Fig 2b Bulk PCA")
```

```
tmp <- scale(pb_PCA$data)

tmp <- tmp[c("ID3.fcs", "ID5.fcs", "ID7.fcs", "ID6.fcs", "ID8.fcs", "ID10.fcs"),]

pheatmap::pheatmap(tmp, scale = "none", cluster_rows = FALSE, cluster_cols = TRUE,
                     breaks = cyCONDOR::scaleColors(data = tmp, maxvalue = NULL)[["breaks"]],
                     color = cyCONDOR::scaleColors(data = tmp, maxvalue = NULL)[["color"]],
                     main = "Fig 2c Pseudobulk marker heatmap", cellwidth = 15, cellheight = 15)
```

# Dimensionality Reduction

## PCA

```
condor <- runPCA(fcd = condor, 
                 data_slot = "orig", 
                 seed = 91)
```

```
plot_dim_red(fcd = condor, 
             expr_slot = "orig", 
             reduction_method = "pca", 
             reduction_slot = "orig", 
             cluster_slot = NULL,
             param = "group", 
             order = T, 
             title = "Figure s3b - UMAP group", 
             facet_by_variable = FALSE, 
             color_discrete = c("#BEBEBE", "#CE2827"), 
             raster = TRUE, 
             dot_size = 0.2, 
             alpha = 1)
```

```
PC_loadings(fcd = condor, data_slot = "orig", nPC = 1)
```

## UMAP

```
condor <- runUMAP(fcd = condor, 
                  input_type = "pca", 
                  data_slot = "orig", 
                  seed = 91)
```

### Rastered UMAP

```
data <- cbind(condor$umap$pca_orig, condor$anno$cell_anno)
```

```
plot_dim_red(fcd = condor, 
             expr_slot = "orig", 
             reduction_method = "umap", 
             reduction_slot = "pca_orig", 
             cluster_slot = NULL,
             param = "group", 
             order = T, 
             title = "Figure 2d - UMAP group", 
             facet_by_variable = FALSE, 
             color_discrete = c("#BEBEBE", "#CE2827"), 
             raster = TRUE)
```

```
plot_dim_density(fcd = condor, 
                 reduction_method = "umap", 
                 reduction_slot = "pca_orig", 
                 group_var = "group", 
                 title = "Figure S2d - Density Map", 
                 dot_size = 0.2, 
                 alpha = 0.2, color_density = c("Greys", "Reds"))
```

## tSNE

```
condor <- runtSNE(fcd = condor, 
                  input_type = "pca", 
                  data_slot = "orig", 
                  seed = 91, 
                  perplexity = 30)
#> Read the 59049 x 28 data matrix successfully!
#> OpenMP is working. 1 threads.
#> Using no_dims = 2, perplexity = 30.000000, and theta = 0.500000
#> Computing input similarities...
#> Building tree...
#>  - point 10000 of 59049
#>  - point 20000 of 59049
#>  - point 30000 of 59049
#>  - point 40000 of 59049
#>  - point 50000 of 59049
#> Done in 72.94 seconds (sparsity = 0.002264)!
#> Learning embedding...
#> Iteration 50: error is 117.869481 (50 iterations in 12.63 seconds)
#> Iteration 100: error is 117.869479 (50 iterations in 16.00 seconds)
#> Iteration 150: error is 117.556145 (50 iterations in 19.71 seconds)
#> Iteration 200: error is 103.993723 (50 iterations in 16.66 seconds)
#> Iteration 250: error is 100.161726 (50 iterations in 13.61 seconds)
#> Iteration 300: error is 4.892023 (50 iterations in 11.66 seconds)
#> Iteration 350: error is 4.581322 (50 iterations in 11.83 seconds)
#> Iteration 400: error is 4.382370 (50 iterations in 11.91 seconds)
#> Iteration 450: error is 4.238309 (50 iterations in 11.82 seconds)
#> Iteration 500: error is 4.126425 (50 iterations in 11.85 seconds)
#> Iteration 550: error is 4.035800 (50 iterations in 11.60 seconds)
#> Iteration 600: error is 3.960285 (50 iterations in 11.60 seconds)
#> Iteration 650: error is 3.895478 (50 iterations in 11.52 seconds)
#> Iteration 700: error is 3.839520 (50 iterations in 11.41 seconds)
#> Iteration 750: error is 3.790388 (50 iterations in 11.35 seconds)
#> Iteration 800: error is 3.746551 (50 iterations in 11.44 seconds)
#> Iteration 850: error is 3.707015 (50 iterations in 11.75 seconds)
#> Iteration 900: error is 3.671404 (50 iterations in 11.41 seconds)
#> Iteration 950: error is 3.639056 (50 iterations in 11.15 seconds)
#> Iteration 1000: error is 3.609240 (50 iterations in 11.03 seconds)
#> Fitting performed in 251.93 seconds.
```

# Clustering

## Phenograph

```
condor <- runPhenograph(fcd = condor, 
                        input_type = "pca", 
                        data_slot = "orig", 
                        k = 60, 
                        seed = 91)
#> Run Rphenograph starts:
#>   -Input data of 59049 rows and 28 columns
#>   -k is set to 60
#>   Finding nearest neighbors...DONE ~ 38.334 s
#>   Compute jaccard coefficient between nearest-neighbor sets...
#> Presorting knn...
#> presorting DONE ~ 2.196 s
#>   Start jaccard
#> DONE ~ 3.187 s
#>   Build undirected graph from the weighted links...DONE ~ 1.66 s
#>   Run louvain clustering on the graph ...DONE ~ 10.63 s
#> Run Rphenograph DONE, totally takes 53.811s.
#>   Return a community class
#>   -Modularity value: 0.8749651 
#>   -Number of clusters: 25
```

```
plot_dim_red(fcd = condor, 
             expr_slot = "orig", 
             reduction_method = "umap", 
             reduction_slot = "pca_orig", 
             cluster_slot = "phenograph_pca_orig_k_60",
             param = "Phenograph", 
             order = T, 
             title = "Figure 2e - UMAP Phenograph clustering", 
             facet_by_variable = FALSE, 
             raster = TRUE, label_clusters = F)
```

```
plot_marker_HM(fcd = condor, 
               expr_slot = "orig", 
               cluster_slot = "phenograph_pca_orig_k_60", 
               cluster_var = "Phenograph",
               maxvalue = 2, 
               marker_to_exclude = c("FSC-A", "SSC-A"),
               title = "Figure S3b - Marker expression Phenograph clustering", 
               cluster_rows = TRUE)
```

## FlowSOM

```
condor <- runFlowSOM(fcd = condor, 
                     input_type = "pca", 
                     data_slot = "orig", 
                     nClusters = 15, 
                     seed = 91, 
                     prefix = NULL, 
                     ret_model = TRUE)
#> Building SOM
#> Mapping data to SOM
#> Building MST
```

```
plot_dim_red(fcd = condor, 
             expr_slot = "orig", 
             reduction_method = "umap", 
             reduction_slot = "pca_orig", 
             cluster_slot = "FlowSOM_pca_orig_k_15",
             param = "FlowSOM", 
             order = T, 
             title = "Figure S3c - UMAP FlowSOM Clustering", 
             facet_by_variable = FALSE, 
             raster = TRUE)
```

### Visualization of the SOM

```
cluster_palette <- c("#89C5DA", "#DA5724", "#74D944", "#CE50CA", "#3F4921", "#C0717C", "#CBD588", "#5F7FC7",
                     "#673770", "#D3D93E", "#38333E", "#508578", "#D7C1B1", "#689030", "#AD6F3B", "#CD9BCD",
                     "#D14285", "#6DDE88", "#652926", "#7FDCC0", "#C84248", "#8569D5", "#5E738F", "#D1A33D",
                     "#8A7C64", "#599861", "#89C5DA", "#DA5724", "#74D944", "#CE50CA", "#3F4921", "#C0717C", "#CBD588", "#5F7FC7",
                     "#673770", "#D3D93E", "#38333E", "#508578", "#D7C1B1", "#689030", "#AD6F3B", "#CD9BCD",
                     "#D14285", "#6DDE88", "#652926", "#7FDCC0", "#C84248", "#8569D5", "#5E738F", "#D1A33D",
                     "#8A7C64", "#599861")

FlowSOM::PlotPies(condor$extras$FlowSOM_model, cellTypes = condor$clustering$FlowSOM_pca_orig_k_15$FlowSOM, colorPalette = cluster_palette) + ggtitle("Figure S3d - FlowSOM PiePlot")
```

```
plot_marker_HM(fcd = condor, 
               expr_slot = "orig", 
               cluster_slot = "FlowSOM_pca_orig_k_15", 
               cluster_var = "FlowSOM",
               maxvalue = 2, 
               marker_to_exclude = c("FSC-A", "SSC-A"),
               title = "Figure S3e - Marker expression FlowSOM clustering", 
               cluster_rows = TRUE)
```

# Plot single markers - Figure S2e

```
marker_exp_plot <- list()

for (marker in colnames(condor$expr$orig)[3:28]) {
  
  marker_exp_plot[[marker]] <- plot_dim_red(fcd = condor, 
                                            expr_slot = "orig", 
                                            reduction_method = "umap", 
                                            reduction_slot = "pca_orig", 
                                            cluster_slot = NULL,
                                            param = marker, 
                                            order = F, 
                                            title = marker, 
                                            facet_by_variable = FALSE, 
                                            raster = TRUE, 
                                            remove_guide = TRUE)
  
}

ggarrange(plotlist = marker_exp_plot)
```

# Metaclustering

```
condor <- metaclustering(fcd = condor, 
                         cluster_slot = "phenograph_pca_orig_k_60",
                         cluster_var = "Phenograph", 
                         cluster_var_new = "metaclusters", 
                         metaclusters = c("1" = "Classical Monocytes",
                                          "2" = "CD4 CD45RA+ CD127+",
                                          "3" = "CD8 CD45RA+ CD127+", 
                                          "4" = "NK dim",
                                          "5" = "CD8 CD45RA+ CD127-",
                                          "6" = "Classical Monocytes",
                                          "7" = "Unconventional T cells", 
                                          "8" = "CD4 CD45RA- CD127+",
                                          "9" = "CD16+ Monocytes",
                                          "10" = "CD4 CD127-",
                                          "11" = "Classical Monocytes", 
                                          "12" = "CD8 CD45RA- CD127+", 
                                          "13" = "CD8 CD45RA- CD127+",
                                          "14" = "NK bright",
                                          "15" = "CD8 CD45RA+ CD127-",
                                          "16" = "CD4 CD25+",
                                          "17" = "B cells",
                                          "18" = "Unconventional T cells",
                                          "19" = "Classical Monocytes",
                                          "20" = "pDCs",
                                          "21" = "CD8 CD45RA+ CD127+",
                                          "22" = "Basophils",
                                          "23" = "Mixed",
                                          "24" = "B cells",
                                          "25" = "NK bright"))
#>    cluster            metacluster
#> 1        1    Classical Monocytes
#> 2        2     CD4 CD45RA+ CD127+
#> 3        3     CD8 CD45RA+ CD127+
#> 4        4                 NK dim
#> 5        5     CD8 CD45RA+ CD127-
#> 6        6    Classical Monocytes
#> 7        7 Unconventional T cells
#> 8        8     CD4 CD45RA- CD127+
#> 9        9        CD16+ Monocytes
#> 10      10             CD4 CD127-
#> 11      11    Classical Monocytes
#> 12      12     CD8 CD45RA- CD127+
#> 13      13     CD8 CD45RA- CD127+
#> 14      14              NK bright
#> 15      15     CD8 CD45RA+ CD127-
#> 16      16              CD4 CD25+
#> 17      17                B cells
#> 18      18 Unconventional T cells
#> 19      19    Classical Monocytes
#> 20      20                   pDCs
#> 21      21     CD8 CD45RA+ CD127+
#> 22      22              Basophils
#> 23      23                  Mixed
#> 24      24                B cells
#> 25      25              NK bright
```

```
plot_dim_red(fcd = condor, 
             expr_slot = "orig", 
             reduction_method = "umap", 
             reduction_slot = "pca_orig", 
             cluster_slot = "phenograph_pca_orig_k_60",
             param = "metaclusters", 
             order = T, 
             title = "Figure 2f - UMAP Metaclustering", 
             facet_by_variable = FALSE, 
             raster = TRUE, 
             color_discrete = rev(cluster_palette), label_clusters = FALSE)
```

```
plot_marker_HM(fcd = condor, 
               expr_slot = "orig", 
               cluster_slot = "phenograph_pca_orig_k_60", 
               cluster_var = "metaclusters",
               maxvalue = 2, 
               marker_to_exclude = c("FSC-A", "SSC-A"),
               title = "Figure 2f - Marker expression Metaclusters", 
               cluster_rows = TRUE)
```

# tSNE visualization clusters

```
plot_dim_red(fcd = condor, 
             expr_slot = "orig", 
             reduction_method = "tSNE", 
             reduction_slot = "pca_orig", 
             cluster_slot = "phenograph_pca_orig_k_60",
             param = "Phenograph", 
             order = T, 
             title = "Figure S3a - tSNE Phenograph", 
             facet_by_variable = FALSE, 
             raster = TRUE)
```

# Session Info

```
info <- sessionInfo()

info
#> R version 4.3.1 (2023-06-16)
#> Platform: x86_64-pc-linux-gnu (64-bit)
#> Running under: Ubuntu 22.04.3 LTS
#> 
#> Matrix products: default
#> BLAS:   /usr/lib/x86_64-linux-gnu/openblas-pthread/libblas.so.3 
#> LAPACK: /usr/lib/x86_64-linux-gnu/openblas-pthread/libopenblasp-r0.3.20.so;  LAPACK version 3.10.0
#> 
#> locale:
#>  [1] LC_CTYPE=en_US.UTF-8       LC_NUMERIC=C              
#>  [3] LC_TIME=en_US.UTF-8        LC_COLLATE=en_US.UTF-8    
#>  [5] LC_MONETARY=en_US.UTF-8    LC_MESSAGES=en_US.UTF-8   
#>  [7] LC_PAPER=en_US.UTF-8       LC_NAME=C                 
#>  [9] LC_ADDRESS=C               LC_TELEPHONE=C            
#> [11] LC_MEASUREMENT=en_US.UTF-8 LC_IDENTIFICATION=C       
#> 
#> time zone: Etc/UTC
#> tzcode source: system (glibc)
#> 
#> attached base packages:
#> [1] stats     graphics  grDevices utils     datasets  methods   base     
#> 
#> other attached packages:
#> [1] RColorBrewer_1.1-3 ggrastr_1.0.2      ggpubr_0.6.0       dplyr_1.1.3       
#> [5] ggsci_3.0.0        ggplot2_3.4.4      cyCONDOR_0.2.0    
#> 
#> loaded via a namespace (and not attached):
#>   [1] IRanges_2.34.1              Rmisc_1.5.1                
#>   [3] urlchecker_1.0.1            nnet_7.3-19                
#>   [5] CytoNorm_2.0.1              TH.data_1.1-2              
#>   [7] vctrs_0.6.4                 digest_0.6.33              
#>   [9] png_0.1-8                   shape_1.4.6                
#>  [11] proxy_0.4-27                slingshot_2.8.0            
#>  [13] ggrepel_0.9.4               parallelly_1.36.0          
#>  [15] MASS_7.3-60                 reshape2_1.4.4             
#>  [17] httpuv_1.6.12               foreach_1.5.2              
#>  [19] BiocGenerics_0.46.0         withr_2.5.1                
#>  [21] xfun_0.40                   ellipsis_0.3.2             
#>  [23] survival_3.5-7              memoise_2.0.1              
#>  [25] hexbin_1.28.3               ggbeeswarm_0.7.2           
#>  [27] RProtoBufLib_2.12.1         princurve_2.1.6            
#>  [29] profvis_0.3.8               zoo_1.8-12                 
#>  [31] GlobalOptions_0.1.2         DEoptimR_1.1-3             
#>  [33] Formula_1.2-5               prettyunits_1.2.0          
#>  [35] promises_1.2.1              scatterplot3d_0.3-44       
#>  [37] rstatix_0.7.2               globals_0.16.2             
#>  [39] ps_1.7.5                    rstudioapi_0.15.0          
#>  [41] miniUI_0.1.1.1              generics_0.1.3             
#>  [43] ggcyto_1.28.1               base64enc_0.1-3            
#>  [45] processx_3.8.2              curl_5.1.0                 
#>  [47] S4Vectors_0.38.2            zlibbioc_1.46.0            
#>  [49] flowWorkspace_4.12.2        polyclip_1.10-6            
#>  [51] randomForest_4.7-1.1        GenomeInfoDbData_1.2.10    
#>  [53] RBGL_1.76.0                 ncdfFlow_2.46.0            
#>  [55] RcppEigen_0.3.3.9.4         xtable_1.8-4               
#>  [57] stringr_1.5.0               doParallel_1.0.17          
#>  [59] evaluate_0.22               S4Arrays_1.0.6             
#>  [61] hms_1.1.3                   glmnet_4.1-8               
#>  [63] GenomicRanges_1.52.1        irlba_2.3.5.1              
#>  [65] colorspace_2.1-0            isoband_0.2.7              
#>  [67] harmony_1.1.0               reticulate_1.34.0          
#>  [69] readxl_1.4.3                magrittr_2.0.3             
#>  [71] lmtest_0.9-40               readr_2.1.4                
#>  [73] Rgraphviz_2.44.0            later_1.3.1                
#>  [75] lattice_0.22-5              future.apply_1.11.0        
#>  [77] robustbase_0.99-0           XML_3.99-0.15              
#>  [79] cowplot_1.1.1               matrixStats_1.1.0          
#>  [81] RcppAnnoy_0.0.21            xts_0.13.1                 
#>  [83] class_7.3-22                Hmisc_5.1-1                
#>  [85] pillar_1.9.0                nlme_3.1-163               
#>  [87] iterators_1.0.14            compiler_4.3.1             
#>  [89] RSpectra_0.16-1             stringi_1.7.12             
#>  [91] gower_1.0.1                 minqa_1.2.6                
#>  [93] SummarizedExperiment_1.30.2 lubridate_1.9.3            
#>  [95] devtools_2.4.5              CytoML_2.12.0              
#>  [97] plyr_1.8.9                  crayon_1.5.2               
#>  [99] abind_1.4-5                 locfit_1.5-9.8             
#> [101] sp_2.1-1                    sandwich_3.0-2             
#> [103] pcaMethods_1.92.0           codetools_0.2-19           
#> [105] multcomp_1.4-25             recipes_1.0.8              
#> [107] openssl_2.1.1               Rphenograph_0.99.1         
#> [109] TTR_0.24.3                  bslib_0.5.1                
#> [111] e1071_1.7-13                destiny_3.14.0             
#> [113] GetoptLong_1.0.5            ggplot.multistats_1.0.0    
#> [115] mime_0.12                   splines_4.3.1              
#> [117] circlize_0.4.15             Rcpp_1.0.11                
#> [119] sparseMatrixStats_1.12.2    cellranger_1.1.0           
#> [121] knitr_1.44                  utf8_1.2.4                 
#> [123] clue_0.3-65                 lme4_1.1-35.1              
#> [125] fs_1.6.3                    listenv_0.9.0              
#> [127] checkmate_2.3.0             DelayedMatrixStats_1.22.6  
#> [129] pkgbuild_1.4.2              ggsignif_0.6.4             
#> [131] tibble_3.2.1                Matrix_1.6-1.1             
#> [133] rpart.plot_3.1.1            callr_3.7.3                
#> [135] tzdb_0.4.0                  tweenr_2.0.2               
#> [137] pkgconfig_2.0.3             pheatmap_1.0.12            
#> [139] tools_4.3.1                 cachem_1.0.8               
#> [141] smoother_1.1                fastmap_1.1.1              
#> [143] rmarkdown_2.25              scales_1.2.1               
#> [145] grid_4.3.1                  usethis_2.2.2              
#> [147] broom_1.0.5                 sass_0.4.7                 
#> [149] graph_1.78.0                carData_3.0-5              
#> [151] RANN_2.6.1                  rpart_4.1.21               
#> [153] farver_2.1.1                yaml_2.3.7                 
#> [155] MatrixGenerics_1.12.3       foreign_0.8-85             
#> [157] ggthemes_4.2.4              cli_3.6.1                  
#> [159] purrr_1.0.2                 stats4_4.3.1               
#> [161] lifecycle_1.0.3             uwot_0.1.16                
#> [163] askpass_1.2.0               caret_6.0-94               
#> [165] Biobase_2.60.0              mvtnorm_1.2-3              
#> [167] lava_1.7.3                  sessioninfo_1.2.2          
#> [169] backports_1.4.1             cytolib_2.12.1             
#> [171] timechange_0.2.0            gtable_0.3.4               
#> [173] rjson_0.2.21                umap_0.2.10.0              
#> [175] ggridges_0.5.4              Rphenoannoy_0.1.0          
#> [177] parallel_4.3.1              pROC_1.18.5                
#> [179] limma_3.56.2                jsonlite_1.8.7             
#> [181] edgeR_3.42.4                RcppHNSW_0.5.0             
#> [183] bitops_1.0-7                Rtsne_0.16                 
#> [185] FlowSOM_2.8.0               ranger_0.16.0              
#> [187] flowCore_2.12.2             jquerylib_0.1.4            
#> [189] timeDate_4022.108           shiny_1.7.5.1              
#> [191] ConsensusClusterPlus_1.64.0 htmltools_0.5.6.1          
#> [193] diffcyt_1.20.0              glue_1.6.2                 
#> [195] XVector_0.40.0              VIM_6.2.2                  
#> [197] RCurl_1.98-1.13             gridExtra_2.3              
#> [199] boot_1.3-28.1               igraph_1.5.1               
#> [201] TrajectoryUtils_1.8.0       R6_2.5.1                   
#> [203] tidyr_1.3.0                 SingleCellExperiment_1.22.0
#> [205] labeling_0.4.3              vcd_1.4-11                 
#> [207] cluster_2.1.4               pkgload_1.3.3              
#> [209] GenomeInfoDb_1.36.4         ipred_0.9-14               
#> [211] nloptr_2.0.3                DelayedArray_0.26.7        
#> [213] tidyselect_1.2.0            vipor_0.4.5                
#> [215] htmlTable_2.4.2             ggforce_0.4.1              
#> [217] CytoDx_1.20.0               car_3.1-2                  
#> [219] future_1.33.0               ModelMetrics_1.2.2.2       
#> [221] munsell_0.5.0               laeken_0.5.2               
#> [223] data.table_1.14.8           htmlwidgets_1.6.2          
#> [225] ComplexHeatmap_2.16.0       rlang_1.1.1                
#> [227] remotes_2.4.2.1             colorRamps_2.3.1           
#> [229] Cairo_1.6-1                 ggnewscale_0.4.9           
#> [231] fansi_1.0.5                 hardhat_1.3.0              
#> [233] beeswarm_0.4.0              prodlim_2023.08.28
```
