# Supplementary material for: Unveiling the power of high-dimensional cytometry data with cyCONDOR
Source: Nat Commun. 2024 Dec 19;15:10702. doi: 10.1038/s41467-024-55179-w (PMC11659560; doi:10.1038/s41467-024-55179-w)
Supplement: Supplementary file 14 — Supplementary Data 12 [file 41467_2024_55179_MOESM14_ESM.html]

Supplementary Data 12: reproducibility data for Figure 2 - CyTOF example dataset


# Supplementary Data 12: reproducibility data for Figure 2 - CyTOF example dataset

# Loading required packages

```
library(cyCONDOR, quietly = T)
library(ggplot2)
library(ggsci)
library(dplyr)
library(ggpubr)
```

# Loading the data

```
condor <- prep_fcd(data_path = "./data/CyTOF_fcs/", 
                    max_cell = 5000, 
                    useCSV = FALSE, 
                    transformation = "auto_logi", 
                    remove_param = c("Cell_length", "(Rh103)Di", "(Ce138)Di", "(La139)Di", "(Ce140)Di", "(Nd145)Di", "Ir191", "Ir193", "File Number", "Time"), 
                    anno_table = "./data/CyTOF_Blood_RA_FR-FCM-Z293.csv", 
                    filename_col = "filename",
                    seed = 91, 
                    verbose = TRUE)
#> [1] "Start reading the data"
#> [1] "Loading file 1 out of 4"
#> [1] "Loading file 2 out of 4"
#> [1] "Loading file 3 out of 4"
#> [1] "Loading file 4 out of 4"
#> [1] "Start transforming the data"
#> [1] "CD66 w= 0.450696594686834 t= 3809.9638671875"
#> [1] "HLADR w= 0.29871629492215 t= 7616.49072265625"
#> [1] "CD3 w= 0.554043411983728 t= 2287.66357421875"
#> [1] "CD64 w= 0.571518973158057 t= 2161.32006835938"
#> [1] "IL6 w= 0.346604476991339 t= 6095.046875"
#> [1] "CD123 w= 0.415470852684638 t= 4364.83154296875"
#> [1] "IL4 w= 0.385575007160766 t= 5070.51416015625"
#> [1] "CD11a w= 0.259346585274145 t= 9250.4423828125"
#> [1] "CD11b w= 0.263499106320689 t= 8970.099609375"
#> [1] "IL8 w= 0.169576351314102 t= 13640.20703125"
#> [1] "CD16 w= 0.309110832762213 t= 7150.2294921875"
#> [1] "CD23 w= 0.1946413688657 t= 11994.1865234375"
#> [1] "CD86 w= 0.184772013798871 t= 12776.376953125"
#> [1] "CD32 w= 0.212187083010261 t= 11499.0263671875"
#> [1] "MIP1b w= 0.288275091156095 t= 7979.51904296875"
#> [1] "IP10 w= 0.272948029942639 t= 8621.443359375"
#> [1] "TNFa w= 0.338419034396734 t= 6174.85302734375"
#> [1] "IL1a w= 0.260858485877209 t= 8935.0859375"
#> [1] "Perforine w= 0.274599571419447 t= 8438.9677734375"
#> [1] "IL12 w= 0.457338758892604 t= 3684.20825195312"
#> [1] "LILRB2 w= 0.615128236823513 t= 1803.28210449219"
#> [1] "CXCR4 w= 0.215039630626666 t= 10408.7216796875"
#> [1] "TLR2 w= 0.3720295074046 t= 5448.27294921875"
#> [1] "CCR5 w= 0.243370634928372 t= 9793.0859375"
#> [1] "CD28 w= 0.391988577047472 t= 4892.328125"
#> [1] "CD11c w= 0.750960361614346 t= 958.342468261719"
#> [1] "IFNa w= 0.343460253709156 t= 6178.455078125"
#> [1] "CD14 w= 0.220652247236496 t= 10720.8115234375"
#> [1] "IL10 w= 0.535918480789899 t= 2584.29711914062"
#> [1] "TLR7 w= 0.448946651256915 t= 3809.72680664062"
#> [1] "GranzymeB w= 0.215549096428898 t= 11149.2294921875"
#> [1] "CD19 w= 0.365623616796938 t= 5446.96875"
#> [1] "IL1RA w= 0.34705372225224 t= 6038.8046875"
#> [1] "NFKB w= 0.299411692530219 t= 7664.7197265625"
```

```
class(condor)
#> [1] "flow_cytometry_dataframe"
```

# Dimensionality Reduction

## PCA

```
condor <- runPCA(fcd = condor, 
                 data_slot = "orig", 
                 seed = 91)
```

## UMAP

```
condor <- runUMAP(fcd = condor, 
                  input_type = "pca", 
                  data_slot = "orig", 
                  seed = 91)
```

## tSNE

```
condor <- runtSNE(fcd = condor, 
                  input_type = "pca", 
                  data_slot = "orig", 
                  seed = 91, 
                  perplexity = 30)
#> Read the 20000 x 34 data matrix successfully!
#> OpenMP is working. 1 threads.
#> Using no_dims = 2, perplexity = 30.000000, and theta = 0.500000
#> Computing input similarities...
#> Building tree...
#>  - point 10000 of 20000
#>  - point 20000 of 20000
#> Done in 10.78 seconds (sparsity = 0.007109)!
#> Learning embedding...
#> Iteration 50: error is 103.806782 (50 iterations in 3.31 seconds)
#> Iteration 100: error is 101.764372 (50 iterations in 3.36 seconds)
#> Iteration 150: error is 89.163288 (50 iterations in 3.05 seconds)
#> Iteration 200: error is 87.632306 (50 iterations in 2.94 seconds)
#> Iteration 250: error is 87.047610 (50 iterations in 3.02 seconds)
#> Iteration 300: error is 3.802366 (50 iterations in 2.72 seconds)
#> Iteration 350: error is 3.527914 (50 iterations in 2.58 seconds)
#> Iteration 400: error is 3.361369 (50 iterations in 2.60 seconds)
#> Iteration 450: error is 3.247837 (50 iterations in 2.60 seconds)
#> Iteration 500: error is 3.162921 (50 iterations in 2.60 seconds)
#> Iteration 550: error is 3.096518 (50 iterations in 2.64 seconds)
#> Iteration 600: error is 3.042632 (50 iterations in 2.62 seconds)
#> Iteration 650: error is 2.997983 (50 iterations in 2.64 seconds)
#> Iteration 700: error is 2.960086 (50 iterations in 2.62 seconds)
#> Iteration 750: error is 2.927693 (50 iterations in 2.67 seconds)
#> Iteration 800: error is 2.899443 (50 iterations in 2.68 seconds)
#> Iteration 850: error is 2.874639 (50 iterations in 2.72 seconds)
#> Iteration 900: error is 2.852635 (50 iterations in 2.67 seconds)
#> Iteration 950: error is 2.833372 (50 iterations in 2.71 seconds)
#> Iteration 1000: error is 2.816140 (50 iterations in 2.70 seconds)
#> Fitting performed in 55.44 seconds.
```

# Clustering

## Phenograph

```
condor <- runPhenograph(fcd = condor, 
                        input_type = "pca", 
                        data_slot = "orig", 
                        k = 60, 
                        seed = 91)
#> Run Rphenograph starts:
#>   -Input data of 20000 rows and 34 columns
#>   -k is set to 60
#>   Finding nearest neighbors...DONE ~ 10.914 s
#>   Compute jaccard coefficient between nearest-neighbor sets...
#> Presorting knn...
#> presorting DONE ~ 0.733 s
#>   Start jaccard
#> DONE ~ 0.986 s
#>   Build undirected graph from the weighted links...DONE ~ 0.389 s
#>   Run louvain clustering on the graph ...DONE ~ 2.57 s
#> Run Rphenograph DONE, totally takes 14.859s.
#>   Return a community class
#>   -Modularity value: 0.8650738 
#>   -Number of clusters: 23
```

```
plot_dim_red(fcd = condor, 
             expr_slot = "orig", 
             reduction_method = "umap", 
             reduction_slot = "pca_orig", 
             cluster_slot = "phenograph_pca_orig_k_60",
             param = "Phenograph", 
             order = T, 
             title = "Figure 2g - UMAP Phenograph Clustering", 
             facet_by_variable = FALSE, 
             raster = TRUE)
```

```
plot_dim_red(fcd = condor, 
             expr_slot = "orig", 
             reduction_method = "tSNE", 
             reduction_slot = "pca_orig", 
             cluster_slot = "phenograph_pca_orig_k_60",
             param = "Phenograph", 
             order = T, 
             title = "Figure S3f - tSNE Phenograph Clustering", 
             facet_by_variable = FALSE, 
             raster = TRUE)
```

```
plot_marker_HM(fcd = condor, 
               expr_slot = "orig", 
               cluster_slot = "phenograph_pca_orig_k_60", 
               cluster_var = "Phenograph",
               maxvalue = 2, 
               title = "Figure S3g - Marker expression Phenograph clustering", 
               cluster_rows = TRUE, 
               cluster_cols = TRUE)
```

# Session Info

```
info <- sessionInfo()

info
#> R version 4.3.1 (2023-06-16)
#> Platform: x86_64-pc-linux-gnu (64-bit)
#> Running under: Ubuntu 22.04.3 LTS
#> 
#> Matrix products: default
#> BLAS:   /usr/lib/x86_64-linux-gnu/openblas-pthread/libblas.so.3 
#> LAPACK: /usr/lib/x86_64-linux-gnu/openblas-pthread/libopenblasp-r0.3.20.so;  LAPACK version 3.10.0
#> 
#> locale:
#>  [1] LC_CTYPE=en_US.UTF-8       LC_NUMERIC=C              
#>  [3] LC_TIME=en_US.UTF-8        LC_COLLATE=en_US.UTF-8    
#>  [5] LC_MONETARY=en_US.UTF-8    LC_MESSAGES=en_US.UTF-8   
#>  [7] LC_PAPER=en_US.UTF-8       LC_NAME=C                 
#>  [9] LC_ADDRESS=C               LC_TELEPHONE=C            
#> [11] LC_MEASUREMENT=en_US.UTF-8 LC_IDENTIFICATION=C       
#> 
#> time zone: Etc/UTC
#> tzcode source: system (glibc)
#> 
#> attached base packages:
#> [1] stats     graphics  grDevices utils     datasets  methods   base     
#> 
#> other attached packages:
#> [1] ggpubr_0.6.0   dplyr_1.1.3    ggsci_3.0.0    ggplot2_3.4.4  cyCONDOR_0.2.0
#> 
#> loaded via a namespace (and not attached):
#>   [1] IRanges_2.34.1              Rmisc_1.5.1                
#>   [3] urlchecker_1.0.1            nnet_7.3-19                
#>   [5] CytoNorm_2.0.1              TH.data_1.1-2              
#>   [7] vctrs_0.6.4                 digest_0.6.33              
#>   [9] png_0.1-8                   shape_1.4.6                
#>  [11] proxy_0.4-27                slingshot_2.8.0            
#>  [13] ggrepel_0.9.4               parallelly_1.36.0          
#>  [15] MASS_7.3-60                 reshape2_1.4.4             
#>  [17] httpuv_1.6.12               foreach_1.5.2              
#>  [19] BiocGenerics_0.46.0         withr_2.5.1                
#>  [21] ggrastr_1.0.2               xfun_0.40                  
#>  [23] ellipsis_0.3.2              survival_3.5-7             
#>  [25] memoise_2.0.1               hexbin_1.28.3              
#>  [27] ggbeeswarm_0.7.2            RProtoBufLib_2.12.1        
#>  [29] princurve_2.1.6             profvis_0.3.8              
#>  [31] zoo_1.8-12                  GlobalOptions_0.1.2        
#>  [33] DEoptimR_1.1-3              Formula_1.2-5              
#>  [35] prettyunits_1.2.0           promises_1.2.1             
#>  [37] scatterplot3d_0.3-44        rstatix_0.7.2              
#>  [39] globals_0.16.2              ps_1.7.5                   
#>  [41] rstudioapi_0.15.0           miniUI_0.1.1.1             
#>  [43] generics_0.1.3              ggcyto_1.28.1              
#>  [45] base64enc_0.1-3             processx_3.8.2             
#>  [47] curl_5.1.0                  S4Vectors_0.38.2           
#>  [49] zlibbioc_1.46.0             flowWorkspace_4.12.2       
#>  [51] polyclip_1.10-6             randomForest_4.7-1.1       
#>  [53] GenomeInfoDbData_1.2.10     RBGL_1.76.0                
#>  [55] ncdfFlow_2.46.0             RcppEigen_0.3.3.9.4        
#>  [57] xtable_1.8-4                stringr_1.5.0              
#>  [59] doParallel_1.0.17           evaluate_0.22              
#>  [61] S4Arrays_1.0.6              hms_1.1.3                  
#>  [63] glmnet_4.1-8                GenomicRanges_1.52.1       
#>  [65] irlba_2.3.5.1               colorspace_2.1-0           
#>  [67] harmony_1.1.0               reticulate_1.34.0          
#>  [69] readxl_1.4.3                magrittr_2.0.3             
#>  [71] lmtest_0.9-40               readr_2.1.4                
#>  [73] Rgraphviz_2.44.0            later_1.3.1                
#>  [75] lattice_0.22-5              future.apply_1.11.0        
#>  [77] robustbase_0.99-0           XML_3.99-0.15              
#>  [79] cowplot_1.1.1               matrixStats_1.1.0          
#>  [81] RcppAnnoy_0.0.21            xts_0.13.1                 
#>  [83] class_7.3-22                Hmisc_5.1-1                
#>  [85] pillar_1.9.0                nlme_3.1-163               
#>  [87] iterators_1.0.14            compiler_4.3.1             
#>  [89] RSpectra_0.16-1             stringi_1.7.12             
#>  [91] gower_1.0.1                 minqa_1.2.6                
#>  [93] SummarizedExperiment_1.30.2 lubridate_1.9.3            
#>  [95] devtools_2.4.5              CytoML_2.12.0              
#>  [97] plyr_1.8.9                  crayon_1.5.2               
#>  [99] abind_1.4-5                 locfit_1.5-9.8             
#> [101] sp_2.1-1                    sandwich_3.0-2             
#> [103] pcaMethods_1.92.0           codetools_0.2-19           
#> [105] multcomp_1.4-25             recipes_1.0.8              
#> [107] openssl_2.1.1               Rphenograph_0.99.1         
#> [109] TTR_0.24.3                  bslib_0.5.1                
#> [111] e1071_1.7-13                destiny_3.14.0             
#> [113] GetoptLong_1.0.5            ggplot.multistats_1.0.0    
#> [115] mime_0.12                   splines_4.3.1              
#> [117] circlize_0.4.15             Rcpp_1.0.11                
#> [119] sparseMatrixStats_1.12.2    cellranger_1.1.0           
#> [121] knitr_1.44                  utf8_1.2.4                 
#> [123] clue_0.3-65                 lme4_1.1-35.1              
#> [125] fs_1.6.3                    listenv_0.9.0              
#> [127] checkmate_2.3.0             DelayedMatrixStats_1.22.6  
#> [129] pkgbuild_1.4.2              ggsignif_0.6.4             
#> [131] tibble_3.2.1                Matrix_1.6-1.1             
#> [133] rpart.plot_3.1.1            callr_3.7.3                
#> [135] tzdb_0.4.0                  tweenr_2.0.2               
#> [137] pkgconfig_2.0.3             pheatmap_1.0.12            
#> [139] tools_4.3.1                 cachem_1.0.8               
#> [141] smoother_1.1                fastmap_1.1.1              
#> [143] rmarkdown_2.25              scales_1.2.1               
#> [145] grid_4.3.1                  usethis_2.2.2              
#> [147] broom_1.0.5                 sass_0.4.7                 
#> [149] graph_1.78.0                carData_3.0-5              
#> [151] RANN_2.6.1                  rpart_4.1.21               
#> [153] farver_2.1.1                yaml_2.3.7                 
#> [155] MatrixGenerics_1.12.3       foreign_0.8-85             
#> [157] ggthemes_4.2.4              cli_3.6.1                  
#> [159] purrr_1.0.2                 stats4_4.3.1               
#> [161] lifecycle_1.0.3             uwot_0.1.16                
#> [163] askpass_1.2.0               caret_6.0-94               
#> [165] Biobase_2.60.0              mvtnorm_1.2-3              
#> [167] lava_1.7.3                  sessioninfo_1.2.2          
#> [169] backports_1.4.1             cytolib_2.12.1             
#> [171] timechange_0.2.0            gtable_0.3.4               
#> [173] rjson_0.2.21                umap_0.2.10.0              
#> [175] ggridges_0.5.4              Rphenoannoy_0.1.0          
#> [177] parallel_4.3.1              pROC_1.18.5                
#> [179] limma_3.56.2                jsonlite_1.8.7             
#> [181] edgeR_3.42.4                RcppHNSW_0.5.0             
#> [183] bitops_1.0-7                Rtsne_0.16                 
#> [185] FlowSOM_2.8.0               ranger_0.16.0              
#> [187] flowCore_2.12.2             jquerylib_0.1.4            
#> [189] timeDate_4022.108           shiny_1.7.5.1              
#> [191] ConsensusClusterPlus_1.64.0 htmltools_0.5.6.1          
#> [193] diffcyt_1.20.0              glue_1.6.2                 
#> [195] XVector_0.40.0              VIM_6.2.2                  
#> [197] RCurl_1.98-1.13             gridExtra_2.3              
#> [199] boot_1.3-28.1               igraph_1.5.1               
#> [201] TrajectoryUtils_1.8.0       R6_2.5.1                   
#> [203] tidyr_1.3.0                 SingleCellExperiment_1.22.0
#> [205] labeling_0.4.3              vcd_1.4-11                 
#> [207] cluster_2.1.4               pkgload_1.3.3              
#> [209] GenomeInfoDb_1.36.4         ipred_0.9-14               
#> [211] nloptr_2.0.3                DelayedArray_0.26.7        
#> [213] tidyselect_1.2.0            vipor_0.4.5                
#> [215] htmlTable_2.4.2             ggforce_0.4.1              
#> [217] CytoDx_1.20.0               car_3.1-2                  
#> [219] future_1.33.0               ModelMetrics_1.2.2.2       
#> [221] munsell_0.5.0               laeken_0.5.2               
#> [223] data.table_1.14.8           htmlwidgets_1.6.2          
#> [225] ComplexHeatmap_2.16.0       RColorBrewer_1.1-3         
#> [227] rlang_1.1.1                 remotes_2.4.2.1            
#> [229] colorRamps_2.3.1            Cairo_1.6-1                
#> [231] ggnewscale_0.4.9            fansi_1.0.5                
#> [233] hardhat_1.3.0               beeswarm_0.4.0             
#> [235] prodlim_2023.08.28
```
