# Supplementary material for: Unveiling the power of high-dimensional cytometry data with cyCONDOR
Source: Nat Commun. 2024 Dec 19;15:10702. doi: 10.1038/s41467-024-55179-w (PMC11659560; doi:10.1038/s41467-024-55179-w)
Supplement: Supplementary file 16 — Supplementary Data 14 [file 41467_2024_55179_MOESM16_ESM.html]

Supplementary Data 14: reproducibility data for Figure 2 - CITE-seq example dataset


# Supplementary Data 14: reproducibility data for Figure 2 - CITE-seq example dataset

```
library(cyCONDOR, quietly = T)
library(ggplot2)
library(ggsci)
library(dplyr)
library(ggpubr)
library(ggrastr)
```

# Loading the data

*Note* This script requires condor v015

```
condor <- prep_fcd(data_path = "./data/CITE-seq/", 
                    max_cell = 20000, 
                    useCSV = TRUE, 
                    transformation = "clr", 
                    remove_param = c("Time"), 
                    anno_table = "./data/CITE-seq.csv", 
                    filename_col = "filename", 
                    separator_fc_csv = ",",
                    seed = 91, 
                    verbose = TRUE)
#> [1] "Start reading the data"
#> [1] "Loading file 1 out of 1"
#> [1] "Start transforming the data"
```

```
class(condor)
#> [1] "flow_cytometry_dataframe"
```

# Dimensionality Reduction

## PCA

```
condor <- runPCA(fcd = condor, 
                 data_slot = "orig", 
                 seed = 91)
```

## UMAP

```
condor <- runUMAP(fcd = condor, 
                  input_type = "pca", 
                  data_slot = "orig", 
                  seed = 91)
```

## tSNE

```
condor <- runtSNE(fcd = condor, 
                  input_type = "pca", 
                  data_slot = "orig", 
                  seed = 91, 
                  perplexity = 30)
#> Read the 20000 x 30 data matrix successfully!
#> OpenMP is working. 1 threads.
#> Using no_dims = 2, perplexity = 30.000000, and theta = 0.500000
#> Computing input similarities...
#> Building tree...
#>  - point 10000 of 20000
#>  - point 20000 of 20000
#> Done in 9.64 seconds (sparsity = 0.006827)!
#> Learning embedding...
#> Iteration 50: error is 104.531405 (50 iterations in 4.32 seconds)
#> Iteration 100: error is 103.868702 (50 iterations in 10.78 seconds)
#> Iteration 150: error is 93.230283 (50 iterations in 4.07 seconds)
#> Iteration 200: error is 91.891227 (50 iterations in 3.68 seconds)
#> Iteration 250: error is 91.409033 (50 iterations in 3.68 seconds)
#> Iteration 300: error is 3.884969 (50 iterations in 3.12 seconds)
#> Iteration 350: error is 3.586872 (50 iterations in 3.08 seconds)
#> Iteration 400: error is 3.401691 (50 iterations in 3.08 seconds)
#> Iteration 450: error is 3.270344 (50 iterations in 3.05 seconds)
#> Iteration 500: error is 3.172252 (50 iterations in 3.12 seconds)
#> Iteration 550: error is 3.093772 (50 iterations in 3.14 seconds)
#> Iteration 600: error is 3.028790 (50 iterations in 3.11 seconds)
#> Iteration 650: error is 2.974052 (50 iterations in 3.13 seconds)
#> Iteration 700: error is 2.928386 (50 iterations in 3.13 seconds)
#> Iteration 750: error is 2.890085 (50 iterations in 3.08 seconds)
#> Iteration 800: error is 2.857687 (50 iterations in 3.10 seconds)
#> Iteration 850: error is 2.830080 (50 iterations in 3.13 seconds)
#> Iteration 900: error is 2.805851 (50 iterations in 3.12 seconds)
#> Iteration 950: error is 2.784601 (50 iterations in 3.14 seconds)
#> Iteration 1000: error is 2.766019 (50 iterations in 3.18 seconds)
#> Fitting performed in 73.22 seconds.
```

# Clustering

## Phenograph

```
condor <- runPhenograph(fcd = condor, 
                        input_type = "pca", 
                        data_slot = "orig", 
                        k = 30, 
                        seed = 91)
#> Run Rphenograph starts:
#>   -Input data of 20000 rows and 30 columns
#>   -k is set to 30
#>   Finding nearest neighbors...DONE ~ 8.183 s
#>   Compute jaccard coefficient between nearest-neighbor sets...
#> Presorting knn...
#> presorting DONE ~ 0.711 s
#>   Start jaccard
#> DONE ~ 0.151 s
#>   Build undirected graph from the weighted links...DONE ~ 0.689 s
#>   Run louvain clustering on the graph ...DONE ~ 1.728 s
#> Run Rphenograph DONE, totally takes 10.751s.
#>   Return a community class
#>   -Modularity value: 0.8393238 
#>   -Number of clusters: 24
```

```
plot_dim_red(fcd = condor, 
             expr_slot = "orig", 
             reduction_method = "umap", 
             reduction_slot = "pca_orig", 
             cluster_slot = "phenograph_pca_orig_k_30",
             param = "Phenograph", 
             order = T, 
             title = "Figure 2i - UMAP Phenograph Clustering", 
             facet_by_variable = FALSE, 
             raster = TRUE)
```

```
plot_dim_red(fcd = condor, 
             expr_slot = "orig", 
             reduction_method = "tSNE", 
             reduction_slot = "pca_orig", 
             cluster_slot = "phenograph_pca_orig_k_30",
             param = "Phenograph", 
             order = T, 
             title = "Figure S3j - tSNE Phenograph Clustering", 
             facet_by_variable = FALSE, 
             raster = TRUE)
```

```
plot_marker_HM(fcd = condor, 
               expr_slot = "orig", 
               cluster_slot = "phenograph_pca_orig_k_30", 
               cluster_var = "Phenograph",
               maxvalue = 2, 
               title = "Figure S2k - Marker expression Phenograph clustering", 
               cluster_rows = TRUE, 
               cluster_cols = TRUE)
```

# Session Info

```
info <- sessionInfo()

info
#> R version 4.3.1 (2023-06-16)
#> Platform: x86_64-pc-linux-gnu (64-bit)
#> Running under: Ubuntu 22.04.3 LTS
#> 
#> Matrix products: default
#> BLAS:   /usr/lib/x86_64-linux-gnu/openblas-pthread/libblas.so.3 
#> LAPACK: /usr/lib/x86_64-linux-gnu/openblas-pthread/libopenblasp-r0.3.20.so;  LAPACK version 3.10.0
#> 
#> locale:
#>  [1] LC_CTYPE=en_US.UTF-8       LC_NUMERIC=C              
#>  [3] LC_TIME=en_US.UTF-8        LC_COLLATE=en_US.UTF-8    
#>  [5] LC_MONETARY=en_US.UTF-8    LC_MESSAGES=en_US.UTF-8   
#>  [7] LC_PAPER=en_US.UTF-8       LC_NAME=C                 
#>  [9] LC_ADDRESS=C               LC_TELEPHONE=C            
#> [11] LC_MEASUREMENT=en_US.UTF-8 LC_IDENTIFICATION=C       
#> 
#> time zone: Etc/UTC
#> tzcode source: system (glibc)
#> 
#> attached base packages:
#> [1] stats     graphics  grDevices utils     datasets  methods   base     
#> 
#> other attached packages:
#> [1] ggrastr_1.0.2  ggpubr_0.6.0   dplyr_1.1.3    ggsci_3.0.0    ggplot2_3.4.4 
#> [6] cyCONDOR_0.2.0
#> 
#> loaded via a namespace (and not attached):
#>   [1] IRanges_2.34.1              Rmisc_1.5.1                
#>   [3] urlchecker_1.0.1            nnet_7.3-19                
#>   [5] CytoNorm_2.0.1              TH.data_1.1-2              
#>   [7] vctrs_0.6.4                 digest_0.6.33              
#>   [9] png_0.1-8                   shape_1.4.6                
#>  [11] proxy_0.4-27                slingshot_2.8.0            
#>  [13] ggrepel_0.9.4               parallelly_1.36.0          
#>  [15] MASS_7.3-60                 reshape2_1.4.4             
#>  [17] httpuv_1.6.12               foreach_1.5.2              
#>  [19] BiocGenerics_0.46.0         withr_2.5.1                
#>  [21] xfun_0.40                   ellipsis_0.3.2             
#>  [23] survival_3.5-7              memoise_2.0.1              
#>  [25] hexbin_1.28.3               ggbeeswarm_0.7.2           
#>  [27] RProtoBufLib_2.12.1         princurve_2.1.6            
#>  [29] profvis_0.3.8               zoo_1.8-12                 
#>  [31] GlobalOptions_0.1.2         DEoptimR_1.1-3             
#>  [33] Formula_1.2-5               prettyunits_1.2.0          
#>  [35] promises_1.2.1              scatterplot3d_0.3-44       
#>  [37] rstatix_0.7.2               globals_0.16.2             
#>  [39] ps_1.7.5                    rstudioapi_0.15.0          
#>  [41] miniUI_0.1.1.1              generics_0.1.3             
#>  [43] ggcyto_1.28.1               base64enc_0.1-3            
#>  [45] processx_3.8.2              curl_5.1.0                 
#>  [47] S4Vectors_0.38.2            zlibbioc_1.46.0            
#>  [49] flowWorkspace_4.12.2        polyclip_1.10-6            
#>  [51] randomForest_4.7-1.1        GenomeInfoDbData_1.2.10    
#>  [53] RBGL_1.76.0                 ncdfFlow_2.46.0            
#>  [55] RcppEigen_0.3.3.9.4         xtable_1.8-4               
#>  [57] stringr_1.5.0               doParallel_1.0.17          
#>  [59] evaluate_0.22               S4Arrays_1.0.6             
#>  [61] hms_1.1.3                   glmnet_4.1-8               
#>  [63] GenomicRanges_1.52.1        irlba_2.3.5.1              
#>  [65] colorspace_2.1-0            harmony_1.1.0              
#>  [67] reticulate_1.34.0           readxl_1.4.3               
#>  [69] magrittr_2.0.3              lmtest_0.9-40              
#>  [71] readr_2.1.4                 Rgraphviz_2.44.0           
#>  [73] later_1.3.1                 lattice_0.22-5             
#>  [75] future.apply_1.11.0         robustbase_0.99-0          
#>  [77] XML_3.99-0.15               cowplot_1.1.1              
#>  [79] matrixStats_1.1.0           RcppAnnoy_0.0.21           
#>  [81] xts_0.13.1                  class_7.3-22               
#>  [83] Hmisc_5.1-1                 pillar_1.9.0               
#>  [85] nlme_3.1-163                iterators_1.0.14           
#>  [87] compiler_4.3.1              RSpectra_0.16-1            
#>  [89] stringi_1.7.12              gower_1.0.1                
#>  [91] minqa_1.2.6                 SummarizedExperiment_1.30.2
#>  [93] lubridate_1.9.3             devtools_2.4.5             
#>  [95] CytoML_2.12.0               plyr_1.8.9                 
#>  [97] crayon_1.5.2                abind_1.4-5                
#>  [99] locfit_1.5-9.8              sp_2.1-1                   
#> [101] sandwich_3.0-2              pcaMethods_1.92.0          
#> [103] codetools_0.2-19            multcomp_1.4-25            
#> [105] recipes_1.0.8               openssl_2.1.1              
#> [107] Rphenograph_0.99.1          TTR_0.24.3                 
#> [109] bslib_0.5.1                 e1071_1.7-13               
#> [111] destiny_3.14.0              GetoptLong_1.0.5           
#> [113] ggplot.multistats_1.0.0     mime_0.12                  
#> [115] splines_4.3.1               circlize_0.4.15            
#> [117] Rcpp_1.0.11                 sparseMatrixStats_1.12.2   
#> [119] cellranger_1.1.0            knitr_1.44                 
#> [121] utf8_1.2.4                  clue_0.3-65                
#> [123] lme4_1.1-35.1               fs_1.6.3                   
#> [125] listenv_0.9.0               checkmate_2.3.0            
#> [127] DelayedMatrixStats_1.22.6   pkgbuild_1.4.2             
#> [129] ggsignif_0.6.4              tibble_3.2.1               
#> [131] Matrix_1.6-1.1              rpart.plot_3.1.1           
#> [133] callr_3.7.3                 tzdb_0.4.0                 
#> [135] tweenr_2.0.2                pkgconfig_2.0.3            
#> [137] pheatmap_1.0.12             tools_4.3.1                
#> [139] cachem_1.0.8                smoother_1.1               
#> [141] fastmap_1.1.1               rmarkdown_2.25             
#> [143] scales_1.2.1                grid_4.3.1                 
#> [145] usethis_2.2.2               broom_1.0.5                
#> [147] sass_0.4.7                  graph_1.78.0               
#> [149] carData_3.0-5               RANN_2.6.1                 
#> [151] rpart_4.1.21                farver_2.1.1               
#> [153] yaml_2.3.7                  MatrixGenerics_1.12.3      
#> [155] foreign_0.8-85              ggthemes_4.2.4             
#> [157] cli_3.6.1                   purrr_1.0.2                
#> [159] stats4_4.3.1                lifecycle_1.0.3            
#> [161] uwot_0.1.16                 askpass_1.2.0              
#> [163] caret_6.0-94                Biobase_2.60.0             
#> [165] mvtnorm_1.2-3               lava_1.7.3                 
#> [167] sessioninfo_1.2.2           backports_1.4.1            
#> [169] cytolib_2.12.1              timechange_0.2.0           
#> [171] gtable_0.3.4                rjson_0.2.21               
#> [173] umap_0.2.10.0               ggridges_0.5.4             
#> [175] Rphenoannoy_0.1.0           parallel_4.3.1             
#> [177] pROC_1.18.5                 limma_3.56.2               
#> [179] jsonlite_1.8.7              edgeR_3.42.4               
#> [181] RcppHNSW_0.5.0              bitops_1.0-7               
#> [183] Rtsne_0.16                  FlowSOM_2.8.0              
#> [185] ranger_0.16.0               flowCore_2.12.2            
#> [187] jquerylib_0.1.4             timeDate_4022.108          
#> [189] shiny_1.7.5.1               ConsensusClusterPlus_1.64.0
#> [191] htmltools_0.5.6.1           diffcyt_1.20.0             
#> [193] glue_1.6.2                  XVector_0.40.0             
#> [195] VIM_6.2.2                   RCurl_1.98-1.13            
#> [197] gridExtra_2.3               boot_1.3-28.1              
#> [199] igraph_1.5.1                TrajectoryUtils_1.8.0      
#> [201] R6_2.5.1                    tidyr_1.3.0                
#> [203] SingleCellExperiment_1.22.0 labeling_0.4.3             
#> [205] vcd_1.4-11                  cluster_2.1.4              
#> [207] pkgload_1.3.3               GenomeInfoDb_1.36.4        
#> [209] ipred_0.9-14                nloptr_2.0.3               
#> [211] DelayedArray_0.26.7         tidyselect_1.2.0           
#> [213] vipor_0.4.5                 htmlTable_2.4.2            
#> [215] ggforce_0.4.1               CytoDx_1.20.0              
#> [217] car_3.1-2                   future_1.33.0              
#> [219] ModelMetrics_1.2.2.2        munsell_0.5.0              
#> [221] laeken_0.5.2                data.table_1.14.8          
#> [223] htmlwidgets_1.6.2           ComplexHeatmap_2.16.0      
#> [225] RColorBrewer_1.1-3          rlang_1.1.1                
#> [227] remotes_2.4.2.1             colorRamps_2.3.1           
#> [229] Cairo_1.6-1                 ggnewscale_0.4.9           
#> [231] fansi_1.0.5                 hardhat_1.3.0              
#> [233] beeswarm_0.4.0              prodlim_2023.08.28
```
