# Supplementary material for: Unveiling the power of high-dimensional cytometry data with cyCONDOR
Source: Nat Commun. 2024 Dec 19;15:10702. doi: 10.1038/s41467-024-55179-w (PMC11659560; doi:10.1038/s41467-024-55179-w)
Supplement: Supplementary file 17 — Supplementary Data 15 [file 41467_2024_55179_MOESM17_ESM.html]

Supplementary Data 15: reproducibility data for Figure 3 - Batch correction workflow


# Supplementary Data 15: reproducibility data for Figure 3 - Batch correction workflow

```
library(cyCONDOR)
library(ggplot2)
library(ggsci)
library(dplyr)
library(ggrastr)
```

# Loading the data

```
condor <- prep_fcd(data_path = "./data/CureDem/all/", 
                   max_cell = 5000, 
                   useCSV = FALSE, 
                   transformation = "auto_logi", 
                   remove_param = c("Time"), 
                   anno_table = "./data/CureDem/all.csv", 
                   filename_col = "filename",
                   seed = 91, 
                   verbose = TRUE)
#> [1] "Start reading the data"
#> [1] "Loading file 1 out of 9"
#> [1] "Loading file 2 out of 9"
#> [1] "Loading file 3 out of 9"
#> [1] "Loading file 4 out of 9"
#> [1] "Loading file 5 out of 9"
#> [1] "Loading file 6 out of 9"
#> [1] "Loading file 7 out of 9"
#> [1] "Loading file 8 out of 9"
#> [1] "Loading file 9 out of 9"
#> [1] "Start transforming the data"
#> [1] "FSC-A w= 0 t= 244060.421875"
#> [1] "FSC-H w= 0 t= 251039.25"
#> [1] "SSC-A w= 0 t= 125217.46875"
#> [1] "SSC-H w= 0 t= 120821.7265625"
#> [1] "CD45RA w= 0.535987086951411 t= 430051.25"
#> [1] "CD94 w= 1.44905346856755 t= 13573.1630859375"
#> [1] "CD195 w= 1.39327099482644 t= 14148.39453125"
#> [1] "CD8 w= 1.4019469305801 t= 14348.748046875"
#> [1] "CD38 w= 0.862717801077739 t= 272703.625"
#> [1] "CD127 w= 1.60497727873327 t= 8446.59765625"
#> [1] "CD56 w= 1.42106399300575 t= 38219.98828125"
#> [1] "HLA-DR w= 1.11398182760456 t= 159159.890625"
#> [1] "CD32 w= 0.364424992289275 t= 2213640.5"
#> [1] "CD16 w= 1.41238984554325 t= 46237.80078125"
#> [1] "CD19 w= 1.14328637455848 t= 42466.421875"
#> [1] "IgD w= 1.27866093999286 t= 37949.35546875"
#> [1] "CD4 w= 1.09678482972518 t= 76758.203125"
#> [1] "CD64 w= 1.42467286821287 t= 50386.828125"
#> [1] "CD14 w= 1.02027714592099 t= 247490.640625"
#> [1] "CD57 w= 0.482903859658396 t= 516215.125"
#> [1] "CD3 w= 1.16487165878441 t= 47342.30859375"
#> [1] "PD-1 w= 1.91908556726921 t= 2362.58251953125"
#> [1] "CD15 w= 1.06451693532041 t= 70215.265625"
#> [1] "CD27 w= 1.19395349348416 t= 38167.6796875"
#> [1] "CD20 w= 1.11038003804556 t= 73366.5078125"
#> [1] "CD197 w= 0.756978923292817 t= 436610.28125"
#> [1] "CD11c w= 1.15533286054835 t= 15492.81640625"
#> [1] "CD13 w= 0.956790750893093 t= 84301.984375"
#> [1] "CD123 w= 0.953453741941109 t= 121064.6640625"
#> [1] "CD25 w= 0.905119368468185 t= 294416.15625"
```

```
condor$anno$cell_anno$exp <- as.factor(condor$anno$cell_anno$exp)
```

# Dimensionality Reduction

## PCA

```
condor <- runPCA(fcd = condor, 
                 data_slot = "orig", 
                 seed = 91)
```

## UMAP

```
condor <- runUMAP(fcd = condor, 
                  input_type = "pca", 
                  data_slot = "orig", 
                  seed = 91)
```

```
plot_dim_red(fcd = condor, 
             expr_slot = "orig", 
             reduction_method = "umap", 
             reduction_slot = "pca_orig", 
             cluster_slot = NULL,
             param = "exp", 
             order = T, 
             title = "Figure 3b - UMAP by group", 
             facet_by_variable = FALSE, 
             color_discrete = c("#1C75BC", "#BE1E2D", "#8DC63F", "#92278F", "#F15A29"), 
             raster = TRUE)
```

```
plot_dim_red(fcd = condor, 
             expr_slot = "orig", 
             reduction_method = "umap", 
             reduction_slot = "pca_orig", 
             cluster_slot = NULL,
             param = "exp", 
             order = T, 
             title = "Figure 3b - UMAP by group", 
             facet_by_variable = TRUE,
             color_discrete = c("#1C75BC", "#BE1E2D", "#8DC63F", "#92278F", "#F15A29"), 
             raster = TRUE)
```

# Batch correction

## Correct intensities

```
condor <- harmonize_intensities(fcd = condor, 
                                batch = c("exp"), 
                                seed = 91)
#> Warning: HarmonyMatrix is deprecated and will be removed in the future from the
#> API in the future
#> Warning: Warning: The parameters do_pca and npcs are deprecated. They will be ignored for this function call and please remove parameters do_pca and npcs and pass to harmony cell_embeddings directly.
#> This warning is displayed once per session.
#> Transposing data matrix
#> Initializing state using k-means centroids initialization
#> Harmony 1/10
#> Harmony 2/10
#> Harmony 3/10
#> Harmony converged after 3 iterations
```

```
condor <- harmonize_PCA(fcd = condor, 
                        batch = c("exp"), 
                        data_slot = "orig", 
                        seed = 91)
#> Warning: HarmonyMatrix is deprecated and will be removed in the future from the
#> API in the future
#> Transposing data matrix
#> Initializing state using k-means centroids initialization
#> Harmony 1/10
#> Harmony 2/10
#> Harmony 3/10
#> Harmony 4/10
#> Harmony 5/10
#> Harmony converged after 5 iterations
```

```
condor <- runUMAP(fcd = condor, 
                  input_type = "pca", 
                  data_slot = "norm", 
                  seed = 91)
```

```
plot_dim_red(fcd = condor, 
             expr_slot = "orig", 
             reduction_method = "umap", 
             reduction_slot = "pca_norm", 
             cluster_slot = NULL,
             param = "exp", 
             order = T, 
             title = "Figure 3c - UMAP by group", 
             facet_by_variable = FALSE, 
             color_discrete = c("#1C75BC", "#BE1E2D", "#8DC63F", "#92278F", "#F15A29"), 
             raster = TRUE)
```

```
plot_dim_red(fcd = condor, 
             expr_slot = "orig", 
             reduction_method = "umap", 
             reduction_slot = "pca_norm", 
             cluster_slot = NULL,
             param = "exp", 
             order = T, 
             title = "Figure 3c - UMAP by group", 
             facet_by_variable = TRUE,
             color_discrete = c("#1C75BC", "#BE1E2D", "#8DC63F", "#92278F", "#F15A29"), 
             raster = TRUE)
```

# Clustering

```
condor <- runPhenograph(fcd = condor,
                        input_type = "pca",
                        data_slot = "orig",
                        k = 60,
                        seed = 91)
#> Run Rphenograph starts:
#>   -Input data of 45000 rows and 30 columns
#>   -k is set to 60
#>   Finding nearest neighbors...DONE ~ 27.153 s
#>   Compute jaccard coefficient between nearest-neighbor sets...
#> Presorting knn...
#> presorting DONE ~ 1.604 s
#>   Start jaccard
#> DONE ~ 2.392 s
#>   Build undirected graph from the weighted links...DONE ~ 1.848 s
#>   Run louvain clustering on the graph ...DONE ~ 13.598 s
#> Run Rphenograph DONE, totally takes 44.991s.
#>   Return a community class
#>   -Modularity value: 0.8715836 
#>   -Number of clusters: 18
```

```
plot_dim_red(fcd = condor, 
             expr_slot = "orig", 
             reduction_method = "umap", 
             reduction_slot = "pca_orig", 
             cluster_slot = "phenograph_pca_orig_k_60",
             param = "Phenograph", 
             order = T, 
             title = "Figure 2d - UMAP Phenograph clustering", 
             facet_by_variable = FALSE, 
             raster = TRUE)
```

Here we do not use native cyCONDOR code because we want to keep the
max value in the HM consistent across examples, normally the function
`plot_confusion_HM` can be used.

```
cells_cluster <- confusionMatrix(paste0(condor$clustering$phenograph_pca_orig_k_60$Phenograph), 
                                 paste0(condor$anno$cell_anno$exp))
cells_cluster <- cells_cluster[order(factor(rownames(cells_cluster), levels = c(0:nrow(cells_cluster)))), ]
cells_cluster <- cells_cluster[, order(colnames(cells_cluster))]
cells_cluster <- as.matrix(cells_cluster)

tmp <- round(t(t(cells_cluster)/colSums(cells_cluster)) * 1000, 3)

scaled_cM <- round((tmp/Matrix::rowSums(tmp)) * 100, 2)

scaled_cM <- rbind(scaled_cM, c(93, 0,0,0,0))

pheatmap::pheatmap(mat = t(scaled_cM), border_color = "black", 
        display_numbers = TRUE, cluster_rows = FALSE, cluster_cols = FALSE, 
        cellwidth = 30, cellheight = 30, main = "Figure S4c - Confusion - before match correction")
```

```
condor <- runPhenograph(fcd = condor,
                        input_type = "pca",
                        data_slot = "norm",
                        k = 60,
                        seed = 91)
#> Run Rphenograph starts:
#>   -Input data of 45000 rows and 30 columns
#>   -k is set to 60
#>   Finding nearest neighbors...DONE ~ 30.699 s
#>   Compute jaccard coefficient between nearest-neighbor sets...
#> Presorting knn...
#> presorting DONE ~ 1.726 s
#>   Start jaccard
#> DONE ~ 2.355 s
#>   Build undirected graph from the weighted links...DONE ~ 0.907 s
#>   Run louvain clustering on the graph ...DONE ~ 16.966 s
#> Run Rphenograph DONE, totally takes 50.927s.
#>   Return a community class
#>   -Modularity value: 0.8596966 
#>   -Number of clusters: 15
```

```
plot_dim_red(fcd = condor, 
             expr_slot = "orig", 
             reduction_method = "umap", 
             reduction_slot = "pca_norm", 
             cluster_slot = "phenograph_pca_norm_k_60",
             param = "Phenograph", 
             order = T, 
             title = "Figure 3e - Corrected UMAP by cluster", 
             facet_by_variable = FALSE, 
             raster = TRUE)
```

```
cells_cluster <- confusionMatrix(paste0(condor$clustering$phenograph_pca_norm_k_60$Phenograph), 
                                 paste0(condor$anno$cell_anno$exp))
cells_cluster <- cells_cluster[order(factor(rownames(cells_cluster), levels = c(0:nrow(cells_cluster)))), ]
cells_cluster <- cells_cluster[, order(colnames(cells_cluster))]
cells_cluster <- as.matrix(cells_cluster)

tmp <- round(t(t(cells_cluster)/colSums(cells_cluster)) * 1000, 3)

scaled_cM <- round((tmp/Matrix::rowSums(tmp)) * 100, 2)

scaled_cM <- rbind(scaled_cM, c(93, 0,0,0,0))

pheatmap::pheatmap(mat = t(scaled_cM), border_color = "black", 
        display_numbers = TRUE, cluster_rows = FALSE, cluster_cols = FALSE, 
        cellwidth = 30, cellheight = 30, main = "Figure S4d - Confusion after batch correction")
```

```
cells_cluster <- confusionMatrix(paste0(condor$clustering$phenograph_pca_norm_k_60$Phenograph), 
                                 paste0(condor$anno$cell_anno$expfcs_filename))
cells_cluster <- cells_cluster[order(factor(rownames(cells_cluster), levels = c(0:nrow(cells_cluster)))), ]
cells_cluster <- cells_cluster[, order(colnames(cells_cluster))]
cells_cluster <- as.matrix(cells_cluster)

tmp <- round(t(t(cells_cluster)/colSums(cells_cluster)) * 1000, 3)

scaled_cM <- round((tmp/Matrix::rowSums(tmp)) * 100, 2)

scaled_cM <- rbind(scaled_cM, c(93, 0,0,0,0,0,0,0,0))

pheatmap::pheatmap(mat = t(scaled_cM), border_color = "black", 
        display_numbers = TRUE, cluster_rows = FALSE, cluster_cols = FALSE, 
        cellwidth = 30, cellheight = 30, main = "Figure S4e - Confusion after single samples")
```

# Calculate LISI score

```
library(lisi)
```

## Prepare the matrix

```
pre_batch <- cbind(condor$umap$pca_orig, condor$anno$cell_anno)

post_batch <- cbind(condor$umap$pca_norm, condor$anno$cell_anno)

res_pre <- compute_lisi(pre_batch[,c(1,2)], pre_batch, c('exp'))

colnames(res_pre) <- "lisi"

res_post <- compute_lisi(post_batch[,c(1,2)], post_batch, c('exp'))

colnames(res_post) <- "lisi"

lisi_mat_pre <- cbind(pre_batch, res_pre)

lisi_mat_pre$type <- "pre"

lisi_mat_post <- cbind(post_batch, res_post)

lisi_mat_post$type <- "post"

lisi_mat <- rbind(lisi_mat_post, lisi_mat_pre)

lisi_mat$type <- factor(lisi_mat$type, levels = c("pre", "post"))
```

## Visualization

```
ggplot(data = lisi_mat, aes(y = lisi, x = type, fill = type)) +
  geom_jitter_rast(alpha = 0.01, scale =0.5) +
  geom_violin(alpha = 0.8) +
  scale_fill_aaas() +
  theme_bw() +
  theme(aspect.ratio = 2, panel.grid = element_blank()) + 
  ggtitle("Figure S4b - LISI score preand post batch correction")
```

# Session Info

```
info <- sessionInfo()

info
#> R version 4.3.1 (2023-06-16)
#> Platform: x86_64-pc-linux-gnu (64-bit)
#> Running under: Ubuntu 22.04.3 LTS
#> 
#> Matrix products: default
#> BLAS:   /usr/lib/x86_64-linux-gnu/openblas-pthread/libblas.so.3 
#> LAPACK: /usr/lib/x86_64-linux-gnu/openblas-pthread/libopenblasp-r0.3.20.so;  LAPACK version 3.10.0
#> 
#> locale:
#>  [1] LC_CTYPE=en_US.UTF-8       LC_NUMERIC=C              
#>  [3] LC_TIME=en_US.UTF-8        LC_COLLATE=en_US.UTF-8    
#>  [5] LC_MONETARY=en_US.UTF-8    LC_MESSAGES=en_US.UTF-8   
#>  [7] LC_PAPER=en_US.UTF-8       LC_NAME=C                 
#>  [9] LC_ADDRESS=C               LC_TELEPHONE=C            
#> [11] LC_MEASUREMENT=en_US.UTF-8 LC_IDENTIFICATION=C       
#> 
#> time zone: Etc/UTC
#> tzcode source: system (glibc)
#> 
#> attached base packages:
#> [1] stats     graphics  grDevices utils     datasets  methods   base     
#> 
#> other attached packages:
#> [1] lisi_1.0       ggrastr_1.0.2  dplyr_1.1.3    ggsci_3.0.0    ggplot2_3.4.4 
#> [6] cyCONDOR_0.2.0
#> 
#> loaded via a namespace (and not attached):
#>   [1] IRanges_2.34.1              Rmisc_1.5.1                
#>   [3] urlchecker_1.0.1            nnet_7.3-19                
#>   [5] CytoNorm_2.0.1              TH.data_1.1-2              
#>   [7] vctrs_0.6.4                 digest_0.6.33              
#>   [9] png_0.1-8                   shape_1.4.6                
#>  [11] proxy_0.4-27                slingshot_2.8.0            
#>  [13] ggrepel_0.9.4               parallelly_1.36.0          
#>  [15] MASS_7.3-60                 reshape2_1.4.4             
#>  [17] httpuv_1.6.12               foreach_1.5.2              
#>  [19] BiocGenerics_0.46.0         withr_2.5.1                
#>  [21] xfun_0.40                   ggpubr_0.6.0               
#>  [23] ellipsis_0.3.2              survival_3.5-7             
#>  [25] memoise_2.0.1               hexbin_1.28.3              
#>  [27] ggbeeswarm_0.7.2            RProtoBufLib_2.12.1        
#>  [29] princurve_2.1.6             profvis_0.3.8              
#>  [31] zoo_1.8-12                  GlobalOptions_0.1.2        
#>  [33] DEoptimR_1.1-3              Formula_1.2-5              
#>  [35] prettyunits_1.2.0           promises_1.2.1             
#>  [37] scatterplot3d_0.3-44        rstatix_0.7.2              
#>  [39] globals_0.16.2              ps_1.7.5                   
#>  [41] rstudioapi_0.15.0           miniUI_0.1.1.1             
#>  [43] generics_0.1.3              ggcyto_1.28.1              
#>  [45] base64enc_0.1-3             processx_3.8.2             
#>  [47] curl_5.1.0                  S4Vectors_0.38.2           
#>  [49] zlibbioc_1.46.0             flowWorkspace_4.12.2       
#>  [51] polyclip_1.10-6             randomForest_4.7-1.1       
#>  [53] GenomeInfoDbData_1.2.10     RBGL_1.76.0                
#>  [55] ncdfFlow_2.46.0             RcppEigen_0.3.3.9.4        
#>  [57] xtable_1.8-4                stringr_1.5.0              
#>  [59] doParallel_1.0.17           evaluate_0.22              
#>  [61] S4Arrays_1.0.6              hms_1.1.3                  
#>  [63] glmnet_4.1-8                GenomicRanges_1.52.1       
#>  [65] irlba_2.3.5.1               colorspace_2.1-0           
#>  [67] harmony_1.1.0               reticulate_1.34.0          
#>  [69] readxl_1.4.3                magrittr_2.0.3             
#>  [71] lmtest_0.9-40               readr_2.1.4                
#>  [73] Rgraphviz_2.44.0            later_1.3.1                
#>  [75] lattice_0.22-5              future.apply_1.11.0        
#>  [77] robustbase_0.99-0           XML_3.99-0.15              
#>  [79] cowplot_1.1.1               matrixStats_1.1.0          
#>  [81] RcppAnnoy_0.0.21            xts_0.13.1                 
#>  [83] class_7.3-22                Hmisc_5.1-1                
#>  [85] pillar_1.9.0                nlme_3.1-163               
#>  [87] iterators_1.0.14            compiler_4.3.1             
#>  [89] RSpectra_0.16-1             stringi_1.7.12             
#>  [91] gower_1.0.1                 minqa_1.2.6                
#>  [93] SummarizedExperiment_1.30.2 lubridate_1.9.3            
#>  [95] devtools_2.4.5              CytoML_2.12.0              
#>  [97] plyr_1.8.9                  crayon_1.5.2               
#>  [99] abind_1.4-5                 locfit_1.5-9.8             
#> [101] sp_2.1-1                    sandwich_3.0-2             
#> [103] pcaMethods_1.92.0           codetools_0.2-19           
#> [105] multcomp_1.4-25             recipes_1.0.8              
#> [107] openssl_2.1.1               Rphenograph_0.99.1         
#> [109] TTR_0.24.3                  bslib_0.5.1                
#> [111] e1071_1.7-13                destiny_3.14.0             
#> [113] GetoptLong_1.0.5            ggplot.multistats_1.0.0    
#> [115] mime_0.12                   splines_4.3.1              
#> [117] circlize_0.4.15             Rcpp_1.0.11                
#> [119] sparseMatrixStats_1.12.2    cellranger_1.1.0           
#> [121] knitr_1.44                  utf8_1.2.4                 
#> [123] clue_0.3-65                 lme4_1.1-35.1              
#> [125] fs_1.6.3                    listenv_0.9.0              
#> [127] checkmate_2.3.0             DelayedMatrixStats_1.22.6  
#> [129] pkgbuild_1.4.2              ggsignif_0.6.4             
#> [131] tibble_3.2.1                Matrix_1.6-1.1             
#> [133] rpart.plot_3.1.1            callr_3.7.3                
#> [135] tzdb_0.4.0                  tweenr_2.0.2               
#> [137] pkgconfig_2.0.3             pheatmap_1.0.12            
#> [139] tools_4.3.1                 cachem_1.0.8               
#> [141] RhpcBLASctl_0.23-42         smoother_1.1               
#> [143] fastmap_1.1.1               rmarkdown_2.25             
#> [145] scales_1.2.1                grid_4.3.1                 
#> [147] usethis_2.2.2               broom_1.0.5                
#> [149] sass_0.4.7                  graph_1.78.0               
#> [151] carData_3.0-5               RANN_2.6.1                 
#> [153] rpart_4.1.21                farver_2.1.1               
#> [155] yaml_2.3.7                  MatrixGenerics_1.12.3      
#> [157] foreign_0.8-85              ggthemes_4.2.4             
#> [159] cli_3.6.1                   purrr_1.0.2                
#> [161] stats4_4.3.1                lifecycle_1.0.3            
#> [163] uwot_0.1.16                 askpass_1.2.0              
#> [165] caret_6.0-94                Biobase_2.60.0             
#> [167] mvtnorm_1.2-3               lava_1.7.3                 
#> [169] sessioninfo_1.2.2           backports_1.4.1            
#> [171] cytolib_2.12.1              timechange_0.2.0           
#> [173] gtable_0.3.4                rjson_0.2.21               
#> [175] umap_0.2.10.0               ggridges_0.5.4             
#> [177] Rphenoannoy_0.1.0           parallel_4.3.1             
#> [179] pROC_1.18.5                 limma_3.56.2               
#> [181] jsonlite_1.8.7              edgeR_3.42.4               
#> [183] RcppHNSW_0.5.0              bitops_1.0-7               
#> [185] Rtsne_0.16                  FlowSOM_2.8.0              
#> [187] ranger_0.16.0               flowCore_2.12.2            
#> [189] jquerylib_0.1.4             timeDate_4022.108          
#> [191] shiny_1.7.5.1               ConsensusClusterPlus_1.64.0
#> [193] htmltools_0.5.6.1           diffcyt_1.20.0             
#> [195] glue_1.6.2                  XVector_0.40.0             
#> [197] VIM_6.2.2                   RCurl_1.98-1.13            
#> [199] gridExtra_2.3               boot_1.3-28.1              
#> [201] igraph_1.5.1                TrajectoryUtils_1.8.0      
#> [203] R6_2.5.1                    tidyr_1.3.0                
#> [205] SingleCellExperiment_1.22.0 labeling_0.4.3             
#> [207] vcd_1.4-11                  cluster_2.1.4              
#> [209] pkgload_1.3.3               GenomeInfoDb_1.36.4        
#> [211] ipred_0.9-14                nloptr_2.0.3               
#> [213] DelayedArray_0.26.7         tidyselect_1.2.0           
#> [215] vipor_0.4.5                 htmlTable_2.4.2            
#> [217] ggforce_0.4.1               CytoDx_1.20.0              
#> [219] car_3.1-2                   future_1.33.0              
#> [221] ModelMetrics_1.2.2.2        munsell_0.5.0              
#> [223] laeken_0.5.2                data.table_1.14.8          
#> [225] htmlwidgets_1.6.2           ComplexHeatmap_2.16.0      
#> [227] RColorBrewer_1.1-3          rlang_1.1.1                
#> [229] remotes_2.4.2.1             colorRamps_2.3.1           
#> [231] Cairo_1.6-1                 ggnewscale_0.4.9           
#> [233] fansi_1.0.5                 hardhat_1.3.0              
#> [235] beeswarm_0.4.0              prodlim_2023.08.28
```
