# Supplementary material for: Unveiling the power of high-dimensional cytometry data with cyCONDOR
Source: Nat Commun. 2024 Dec 19;15:10702. doi: 10.1038/s41467-024-55179-w (PMC11659560; doi:10.1038/s41467-024-55179-w)
Supplement: Supplementary file 18 — Supplementary Data 16 [file 41467_2024_55179_MOESM18_ESM.html]

Supplementary Data 16: reproducibility data for Figure 4 - Pseudotime analysis workflow


# Supplementary Data 16: reproducibility data for Figure 4 - Pseudotime analysis workflow

```
library(cyCONDOR)
library(ggplot2)
library(ggsci)
library(dplyr)
library(ggpubr)
library(pheatmap)
```

# Loading the data

```
condor <- prep_fcd(data_path = "./Data/CyTOF_BM/", 
                    max_cell = 10000, 
                    useCSV = FALSE, 
                    transformation = "auto_logi", 
                    remove_param = c("Cell Length", "191-DNA", "193-DNA", "EventNum", "110-CD3", "111-CD3", "112-CD3", "113-CD3", "114-CD3", "Time"), 
                    anno_table = "./data/Bendall_et_al_Science_2011_singlets.csv", 
                    filename_col = "filename",
                    seed = 91, 
                    verbose = TRUE)
#> [1] "Start reading the data"
#> [1] "Loading file 1 out of 1"
#> [1] "Start transforming the data"
#> [1] "110_114-CD3 w= 0.494035406408185 t= 25214.306640625"
#> [1] "164-CD15 w= 0.881279581317068 t= 10077.1953125"
#> [1] "166-CD44 w= 0.910254860667858 t= 4695.90771484375"
#> [1] "167-CD7 w= 0.873761002078388 t= 3285.91528320312"
#> [1] "168-CD13 w= 0.601975368814055 t= 7564.45263671875"
#> [1] "170-CD56 w= 0.715743012823327 t= 2758.20239257812"
#> [1] "151-CD123 w= 0.553229064352839 t= 954.899780273438"
#> [1] "153-IgM w= 0.583977313137632 t= 3276.46020507812"
#> [1] "156-CD10 w= 0.745882747008157 t= 1956.94934082031"
#> [1] "158-CD33 w= 0.99732724925751 t= 1705.11877441406"
#> [1] "160-CD14 w= 1.1164174769125 t= 1280.43798828125"
#> [1] "165-CD16 w= 0.754183198180059 t= 3987.7734375"
#> [1] "115-CD45 w= 0.54408057666489 t= 6970.75927734375"
#> [1] "139-CD45RA w= 0.848973358181249 t= 3992.02514648438"
#> [1] "175-CXCR4 w= 0.823214639684549 t= 5624.69970703125"
#> [1] "142-CD19 w= 1.16439163521258 t= 535.895202636719"
#> [1] "144-CD11b w= 0.778389313589042 t= 1897.92724609375"
#> [1] "145-CD4 w= 0.476812738672134 t= 5216.748046875"
#> [1] "146-CD8 w= 0.617277499790798 t= 5215.27783203125"
#> [1] "148-CD34 w= 0.411752713107874 t= 7333.931640625"
#> [1] "150-CD161 w= 0.780490346995322 t= 1180.24365234375"
#> [1] "141-CD235ab w= 0.919394871159076 t= 5890.10498046875"
#> [1] "103-Viability w= 0.899976177989344 t= 1952.2431640625"
#> [1] "147-CD20 w= 0.540950758596191 t= 6252.22119140625"
#> [1] "152-CD41 w= 0.293008766316461 t= 6344.4228515625"
#> [1] "154-CD11c w= 1.17321699872376 t= 235.263916015625"
#> [1] "159-CD38 w= 0.818174324698806 t= 6779.41455078125"
#> [1] "169-CD61 w= 0.622296356703662 t= 4947.873046875"
#> [1] "171-CD117 w= 0.573383498425138 t= 3361.87158203125"
#> [1] "172-CD47 w= 0.859094918610869 t= 4283.056640625"
#> [1] "174-HLADR w= 0.901031154744318 t= 4383.521484375"
#> [1] "176-CD90 w= 0.689668368158964 t= 3264.10620117188"
```

```
class(condor)
#> [1] "flow_cytometry_dataframe"
```

# Dimensionality Reduction

## PCA

```
condor <- runPCA(fcd = condor, 
                 data_slot = "orig", 
                 seed = 91)
```

## UMAP

```
condor <- runUMAP(fcd = condor, 
                  input_type = "pca", 
                  data_slot = "orig", 
                  seed = 91)
```

# Clustering

## Phenograph

```
condor <- runPhenograph(fcd = condor, 
                        input_type = "pca", 
                        data_slot = "orig", 
                        k = 10, 
                        seed = 91)
#> Run Rphenograph starts:
#>   -Input data of 10000 rows and 32 columns
#>   -k is set to 10
#>   Finding nearest neighbors...DONE ~ 2.804 s
#>   Compute jaccard coefficient between nearest-neighbor sets...
#> Presorting knn...
#> presorting DONE ~ 0.307 s
#>   Start jaccard
#> DONE ~ 0.003 s
#>   Build undirected graph from the weighted links...DONE ~ 0.037 s
#>   Run louvain clustering on the graph ...DONE ~ 0.185 s
#> Run Rphenograph DONE, totally takes 3.029s.
#>   Return a community class
#>   -Modularity value: 0.8838145 
#>   -Number of clusters: 26
```

```
plot_dim_red(fcd = condor, 
             expr_slot = "orig", 
             reduction_method = "umap", 
             reduction_slot = "pca_orig", 
             cluster_slot = "phenograph_pca_orig_k_10",
             param = "Phenograph", 
             order = T, 
             title = "Figure S5b - UMAP Phenograph clustering", 
             facet_by_variable = FALSE, 
             raster = TRUE)
```

```
plot_marker_HM(fcd = condor, 
               expr_slot = "orig", 
               cluster_slot = "phenograph_pca_orig_k_10", 
               cluster_var = "Phenograph",
               maxvalue = 2, 
               title = "Figure S5c - Marker expression Phenograph clustering", 
               cluster_rows = TRUE, 
               cluster_cols = TRUE)
#> Warning in qt(conf.interval/2 + 0.5, datac$N - 1): NaNs produced
```

# Metaclustering

```
condor <- metaclustering(fcd = condor, 
                         cluster_slot = "phenograph_pca_orig_k_10", 
                         cluster_var = "Phenograph", 
                         cluster_var_new = "metaclusters", 
                         metaclusters = c("1" = "Mature B cells IL3Ra+", 
                                          "2" = "Monoblast", 
                                          "3" = "Granulocytes", 
                                          "4" = "Platlets", 
                                          "5" = "Mature B cells IL3Ra+", 
                                          "6" = "Erytrocytes", 
                                          "7" = "CD8+ T cells", 
                                          "8" = "Monocytes", 
                                          "9" = "Mature B cells", 
                                          "10" = "Trombocytes", 
                                          "11" = "NK cells", 
                                          "12" = "CD4+ T cells", 
                                          "13" = "Erytroblast",
                                          "14" = "NKT",
                                          "15" = "Erytroblast",
                                          "16" = "HSCs",
                                          "17" = "DP T cells",
                                          "18" = "pDCs",
                                          "19" = "Immature B cells",
                                          "20" = "Monoblast",
                                          "21" = "CD4+ T cells",
                                          "22" = "Plasma cells",
                                          "23" = "Myelocytes",
                                          "24" = "Promyelocytes",
                                          "25" = "CD8+ T cells",
                                          "26" = "CD8+ T cells"))
#>    cluster           metacluster
#> 1        1 Mature B cells IL3Ra+
#> 2        2             Monoblast
#> 3        3          Granulocytes
#> 4        4              Platlets
#> 5        5 Mature B cells IL3Ra+
#> 6        6           Erytrocytes
#> 7        7          CD8+ T cells
#> 8        8             Monocytes
#> 9        9        Mature B cells
#> 10      10           Trombocytes
#> 11      11              NK cells
#> 12      12          CD4+ T cells
#> 13      13           Erytroblast
#> 14      14                   NKT
#> 15      15           Erytroblast
#> 16      16                  HSCs
#> 17      17            DP T cells
#> 18      18                  pDCs
#> 19      19      Immature B cells
#> 20      20             Monoblast
#> 21      21          CD4+ T cells
#> 22      22          Plasma cells
#> 23      23            Myelocytes
#> 24      24         Promyelocytes
#> 25      25          CD8+ T cells
#> 26      26          CD8+ T cells
```

```
plot_dim_red(fcd = condor, 
             expr_slot = "orig", 
             reduction_method = "umap", 
             reduction_slot = "pca_orig", 
             cluster_slot = "phenograph_pca_orig_k_10",
             param = "metaclusters", 
             order = T, 
             title = "Figure 4b - UMAP Metaclusters", 
             facet_by_variable = FALSE, 
             raster = TRUE)
```

# Subset the monocytes - pDCs lineages

```
selections <- rownames(condor$clustering$phenograph_pca_orig_k_10[condor$clustering$phenograph_pca_orig_k_10$metaclusters %in% c("HSCs", "pDCs", "Monoblast", "Monocytes"), ])

condor_filter <- filter_fcd(fcd = condor,
                            cell_ids = selections)
```

## PCA

```
condor_filter <- runPCA(fcd = condor_filter, 
                 data_slot = "orig", 
                 seed = 91)
```

## UMAP

```
condor_filter <- runUMAP(fcd = condor_filter, 
                  input_type = "pca", 
                  data_slot = "orig", 
                  seed = 91)
```

# Clustering

## Phenograph

```
condor_filter <- runPhenograph(fcd = condor_filter, 
                               input_type = "pca", 
                               data_slot = "orig", 
                               k = 10, 
                               seed = 91, 
                               prefix = "filter")
#> Run Rphenograph starts:
#>   -Input data of 1855 rows and 32 columns
#>   -k is set to 10
#>   Finding nearest neighbors...DONE ~ 0.415 s
#>   Compute jaccard coefficient between nearest-neighbor sets...
#> Presorting knn...
#> presorting DONE ~ 0.061 s
#>   Start jaccard
#> DONE ~ 0.001 s
#>   Build undirected graph from the weighted links...DONE ~ 0.006 s
#>   Run louvain clustering on the graph ...DONE ~ 0.017 s
#> Run Rphenograph DONE, totally takes 0.439s.
#>   Return a community class
#>   -Modularity value: 0.7786039 
#>   -Number of clusters: 15
```

```
## Remove contaminating cluster
selections <- rownames(condor_filter$clustering$phenograph_filter_pca_orig_k_10[!condor_filter$clustering$phenograph_filter_pca_orig_k_10$Phenograph %in% c("11"), ])

condor_filter <- filter_fcd(fcd = condor_filter,
                            cell_ids = selections)
```

```
plot_dim_red(fcd = condor_filter, 
             expr_slot = "orig", 
             reduction_method = "umap", 
             reduction_slot = "pca_orig", 
             cluster_slot = "phenograph_filter_pca_orig_k_10",
             param = "Phenograph", 
             order = T, 
             title = "Figure S5d - UMAP by group", 
             facet_by_variable = FALSE, 
             raster = TRUE, 
             alpha = 1, 
             dot_size = 1)
```

```
plot_marker_HM(fcd = condor_filter, 
               expr_slot = "orig", 
               cluster_slot = "phenograph_filter_pca_orig_k_10", 
               cluster_var = "Phenograph",
               maxvalue = 2, 
               title = "Figure S5e - Marker expression Phenograph clustering", 
               cluster_rows = TRUE, 
               cluster_cols = TRUE)
```

# Metaclustering

```
condor_filter <- metaclustering(fcd = condor_filter, 
                         cluster_slot = "phenograph_filter_pca_orig_k_10", 
                         cluster_var = "Phenograph", 
                         cluster_var_new = "metaclusters", 
                         metaclusters = c("1" = "Myelocytes", 
                                          "2" = "Monocytes", 
                                          "3" = "Monocytes", 
                                          "4" = "Monocytes", 
                                          "5" = "HSCs", 
                                          "6" = "Monoblast", 
                                          "7" = "pDCs", 
                                          "8" = "CMPs", 
                                          "9" = "Monocytes", 
                                          "10" = "Pre-DC", 
                                          "11" = "CMPs", 
                                          "12" = "Monoblast", 
                                          "13" = "Monocytes",
                                          "14" = "Monocytes",
                                          "15" = "Monocytes"))
#>    cluster metacluster
#> 1        1  Myelocytes
#> 2        2   Monocytes
#> 3        3   Monocytes
#> 4        4   Monocytes
#> 5        5        HSCs
#> 6        6   Monoblast
#> 7        7        pDCs
#> 8        8        CMPs
#> 9        9   Monocytes
#> 10      10      Pre-DC
#> 11      11        CMPs
#> 12      12   Monoblast
#> 13      13   Monocytes
#> 14      14   Monocytes
#> 15      15   Monocytes
```

```
plot_dim_red(fcd = condor_filter, 
             expr_slot = "orig", 
             reduction_method = "umap", 
             reduction_slot = "pca_orig", 
             cluster_slot = "phenograph_filter_pca_orig_k_10",
             param = "metaclusters", 
             order = T, 
             title = "Figure 4c - UMAP by group", 
             facet_by_variable = FALSE, 
             raster = TRUE, 
             alpha = 1, 
             dot_size = 1)
```

# Pseudotime analysis

```
condor_filter <- runPseudotime(fcd = condor_filter, 
                               reduction_method = "umap", 
                               reduction_slot = "pca_orig", 
                               cluster_slot = "phenograph_filter_pca_orig_k_10", 
                               cluster_var = "metaclusters",
                               approx_points = NULL, 
                               seed = 91)
#> [1] "Slingshot - getLineages"
#> [1] "Slingshot - getCurves"
```

```
plot_dim_red(fcd = condor_filter, 
             expr_slot = "orig", 
             reduction_method = "umap", 
             reduction_slot = "pca_orig", 
             cluster_slot = "phenograph_filter_pca_orig_k_10", 
             add_pseudotime = TRUE, 
             pseudotime_slot = "slingshot_umap_pca_orig",
             param = "mean", 
             order = T, 
             title = "Figure 4e - UMAP by speudotime", 
             facet_by_variable = FALSE, 
             raster = TRUE, 
             alpha = 1, 
             dot_size = 1) + 
  geom_path(data = condor_filter$extras$slingshot_umap_pca_orig$lineages %>% arrange(Order), aes(group = Lineage), size = 0.5)
#> Warning: Using `size` aesthetic for lines was deprecated in ggplot2 3.4.0.
#> ℹ Please use `linewidth` instead.
#> This warning is displayed once every 8 hours.
#> Call `lifecycle::last_lifecycle_warnings()` to see where this warning was
#> generated.
```

# Heatmap visualization of trajectory

## DCs

```
selections <- rownames(condor_filter$clustering$phenograph_filter_pca_orig_k_10[condor_filter$clustering$phenograph_filter_pca_orig_k_10$metaclusters %in% c("HSCs", "Pre-DC", "pDCs"), ])

condor_dcs <- filter_fcd(fcd = condor_filter,
                         cell_ids = selections)
```

```
expression <- condor_dcs$expr$orig

anno <- cbind(condor_dcs$clustering$phenograph_filter_pca_orig_k_10[, c("Phenograph", "metaclusters")], condor_dcs$pseudotime$slingshot_umap_pca_orig)

anno <- anno[order(anno$Lineage2, decreasing = TRUE),]

expression <- expression[rownames(anno), c("174-HLADR", "151-CD123", "148-CD34")]

my_colour = list(metaclusters = c(HSCs = "#689030", pDCs = "#CD9BCD", `Pre-DC` = "#2B3990"))
```

```
pheatmap(mat = expression, 
         scale = "column", 
         show_rownames = FALSE, 
         cluster_rows = F, 
         cluster_cols = F, 
         annotation_row = anno[, c("metaclusters", "Lineage2")], 
         annotation_colors = my_colour, 
         breaks = scaleColors(expression, maxvalue = 2)[["breaks"]], 
         color = scaleColors(expression, maxvalue = 2)[["color"]], 
         main = "Figure S6b - Heatmap pDCs trajectory")
```

## Monocytes

```
selections <- rownames(condor_filter$clustering$phenograph_filter_pca_orig_k_10[condor_filter$clustering$phenograph_filter_pca_orig_k_10$metaclusters %in% c("HSCs", "CMPs", "Monoblast", "Monocytes"), ])

condor_mono <- filter_fcd(fcd = condor_filter,
                          cell_ids = selections)
```

```
expression <- condor_mono$expr$orig

anno <- cbind(condor_mono$clustering$phenograph_filter_pca_orig_k_10[, c("Phenograph", "metaclusters")], condor_mono$pseudotime$slingshot_umap_pca_orig)

anno <- anno[order(anno$Lineage2, decreasing = FALSE),]

expression <- expression[rownames(anno), c("148-CD34", "160-CD14", "144-CD11b")]

my_colour = list(metaclusters = c(Monocytes = "#CBD588", HSCs = "#689030", Monoblast = "#DA5724", CMPs = "#F7941D"))
```

```
pheatmap(mat = expression, 
         scale = "column", 
         show_rownames = FALSE, 
         cluster_rows = F, 
         cluster_cols = F, 
         annotation_row = anno[, c("metaclusters", "Lineage2")], 
         annotation_colors = my_colour, 
         breaks = scaleColors(expression, maxvalue = 2)[["breaks"]], 
         color = scaleColors(expression, maxvalue = 2)[["color"]], main = "Figure 4f - Heatmap Monocytes pseudotime")
```

# Session Info

```
info <- sessionInfo()

info
#> R version 4.3.1 (2023-06-16)
#> Platform: x86_64-pc-linux-gnu (64-bit)
#> Running under: Ubuntu 22.04.3 LTS
#> 
#> Matrix products: default
#> BLAS:   /usr/lib/x86_64-linux-gnu/openblas-pthread/libblas.so.3 
#> LAPACK: /usr/lib/x86_64-linux-gnu/openblas-pthread/libopenblasp-r0.3.20.so;  LAPACK version 3.10.0
#> 
#> locale:
#>  [1] LC_CTYPE=en_US.UTF-8       LC_NUMERIC=C              
#>  [3] LC_TIME=en_US.UTF-8        LC_COLLATE=en_US.UTF-8    
#>  [5] LC_MONETARY=en_US.UTF-8    LC_MESSAGES=en_US.UTF-8   
#>  [7] LC_PAPER=en_US.UTF-8       LC_NAME=C                 
#>  [9] LC_ADDRESS=C               LC_TELEPHONE=C            
#> [11] LC_MEASUREMENT=en_US.UTF-8 LC_IDENTIFICATION=C       
#> 
#> time zone: Etc/UTC
#> tzcode source: system (glibc)
#> 
#> attached base packages:
#> [1] stats     graphics  grDevices utils     datasets  methods   base     
#> 
#> other attached packages:
#> [1] pheatmap_1.0.12 ggpubr_0.6.0    dplyr_1.1.3     ggsci_3.0.0    
#> [5] ggplot2_3.4.4   cyCONDOR_0.2.0 
#> 
#> loaded via a namespace (and not attached):
#>   [1] IRanges_2.34.1              Rmisc_1.5.1                
#>   [3] urlchecker_1.0.1            nnet_7.3-19                
#>   [5] CytoNorm_2.0.1              TH.data_1.1-2              
#>   [7] vctrs_0.6.4                 digest_0.6.33              
#>   [9] png_0.1-8                   shape_1.4.6                
#>  [11] proxy_0.4-27                slingshot_2.8.0            
#>  [13] ggrepel_0.9.4               parallelly_1.36.0          
#>  [15] MASS_7.3-60                 reshape2_1.4.4             
#>  [17] httpuv_1.6.12               foreach_1.5.2              
#>  [19] BiocGenerics_0.46.0         withr_2.5.1                
#>  [21] ggrastr_1.0.2               xfun_0.40                  
#>  [23] ellipsis_0.3.2              survival_3.5-7             
#>  [25] memoise_2.0.1               hexbin_1.28.3              
#>  [27] ggbeeswarm_0.7.2            RProtoBufLib_2.12.1        
#>  [29] princurve_2.1.6             profvis_0.3.8              
#>  [31] zoo_1.8-12                  GlobalOptions_0.1.2        
#>  [33] DEoptimR_1.1-3              Formula_1.2-5              
#>  [35] prettyunits_1.2.0           promises_1.2.1             
#>  [37] scatterplot3d_0.3-44        rstatix_0.7.2              
#>  [39] globals_0.16.2              ps_1.7.5                   
#>  [41] rstudioapi_0.15.0           miniUI_0.1.1.1             
#>  [43] generics_0.1.3              ggcyto_1.28.1              
#>  [45] base64enc_0.1-3             processx_3.8.2             
#>  [47] curl_5.1.0                  S4Vectors_0.38.2           
#>  [49] zlibbioc_1.46.0             flowWorkspace_4.12.2       
#>  [51] polyclip_1.10-6             randomForest_4.7-1.1       
#>  [53] GenomeInfoDbData_1.2.10     RBGL_1.76.0                
#>  [55] ncdfFlow_2.46.0             RcppEigen_0.3.3.9.4        
#>  [57] xtable_1.8-4                stringr_1.5.0              
#>  [59] doParallel_1.0.17           evaluate_0.22              
#>  [61] S4Arrays_1.0.6              hms_1.1.3                  
#>  [63] glmnet_4.1-8                GenomicRanges_1.52.1       
#>  [65] irlba_2.3.5.1               colorspace_2.1-0           
#>  [67] harmony_1.1.0               reticulate_1.34.0          
#>  [69] readxl_1.4.3                magrittr_2.0.3             
#>  [71] lmtest_0.9-40               readr_2.1.4                
#>  [73] Rgraphviz_2.44.0            later_1.3.1                
#>  [75] lattice_0.22-5              future.apply_1.11.0        
#>  [77] robustbase_0.99-0           XML_3.99-0.15              
#>  [79] cowplot_1.1.1               matrixStats_1.1.0          
#>  [81] RcppAnnoy_0.0.21            xts_0.13.1                 
#>  [83] class_7.3-22                Hmisc_5.1-1                
#>  [85] pillar_1.9.0                nlme_3.1-163               
#>  [87] iterators_1.0.14            compiler_4.3.1             
#>  [89] RSpectra_0.16-1             stringi_1.7.12             
#>  [91] gower_1.0.1                 minqa_1.2.6                
#>  [93] SummarizedExperiment_1.30.2 lubridate_1.9.3            
#>  [95] devtools_2.4.5              CytoML_2.12.0              
#>  [97] plyr_1.8.9                  crayon_1.5.2               
#>  [99] abind_1.4-5                 locfit_1.5-9.8             
#> [101] sp_2.1-1                    sandwich_3.0-2             
#> [103] pcaMethods_1.92.0           codetools_0.2-19           
#> [105] multcomp_1.4-25             recipes_1.0.8              
#> [107] openssl_2.1.1               Rphenograph_0.99.1         
#> [109] TTR_0.24.3                  bslib_0.5.1                
#> [111] e1071_1.7-13                destiny_3.14.0             
#> [113] GetoptLong_1.0.5            ggplot.multistats_1.0.0    
#> [115] mime_0.12                   splines_4.3.1              
#> [117] circlize_0.4.15             Rcpp_1.0.11                
#> [119] sparseMatrixStats_1.12.2    cellranger_1.1.0           
#> [121] knitr_1.44                  utf8_1.2.4                 
#> [123] clue_0.3-65                 lme4_1.1-35.1              
#> [125] fs_1.6.3                    listenv_0.9.0              
#> [127] checkmate_2.3.0             DelayedMatrixStats_1.22.6  
#> [129] pkgbuild_1.4.2              ggsignif_0.6.4             
#> [131] tibble_3.2.1                Matrix_1.6-1.1             
#> [133] rpart.plot_3.1.1            callr_3.7.3                
#> [135] tzdb_0.4.0                  tweenr_2.0.2               
#> [137] pkgconfig_2.0.3             tools_4.3.1                
#> [139] cachem_1.0.8                smoother_1.1               
#> [141] fastmap_1.1.1               rmarkdown_2.25             
#> [143] scales_1.2.1                grid_4.3.1                 
#> [145] usethis_2.2.2               broom_1.0.5                
#> [147] sass_0.4.7                  FNN_1.1.3.2                
#> [149] graph_1.78.0                carData_3.0-5              
#> [151] RANN_2.6.1                  rpart_4.1.21               
#> [153] farver_2.1.1                yaml_2.3.7                 
#> [155] MatrixGenerics_1.12.3       foreign_0.8-85             
#> [157] ggthemes_4.2.4              cli_3.6.1                  
#> [159] purrr_1.0.2                 stats4_4.3.1               
#> [161] lifecycle_1.0.3             uwot_0.1.16                
#> [163] askpass_1.2.0               caret_6.0-94               
#> [165] Biobase_2.60.0              mvtnorm_1.2-3              
#> [167] lava_1.7.3                  sessioninfo_1.2.2          
#> [169] backports_1.4.1             cytolib_2.12.1             
#> [171] timechange_0.2.0            gtable_0.3.4               
#> [173] rjson_0.2.21                umap_0.2.10.0              
#> [175] ggridges_0.5.4              Rphenoannoy_0.1.0          
#> [177] parallel_4.3.1              pROC_1.18.5                
#> [179] limma_3.56.2                jsonlite_1.8.7             
#> [181] edgeR_3.42.4                RcppHNSW_0.5.0             
#> [183] bitops_1.0-7                Rtsne_0.16                 
#> [185] FlowSOM_2.8.0               ranger_0.16.0              
#> [187] flowCore_2.12.2             jquerylib_0.1.4            
#> [189] timeDate_4022.108           shiny_1.7.5.1              
#> [191] ConsensusClusterPlus_1.64.0 htmltools_0.5.6.1          
#> [193] diffcyt_1.20.0              glue_1.6.2                 
#> [195] XVector_0.40.0              VIM_6.2.2                  
#> [197] RCurl_1.98-1.13             gridExtra_2.3              
#> [199] boot_1.3-28.1               igraph_1.5.1               
#> [201] TrajectoryUtils_1.8.0       R6_2.5.1                   
#> [203] tidyr_1.3.0                 SingleCellExperiment_1.22.0
#> [205] labeling_0.4.3              vcd_1.4-11                 
#> [207] cluster_2.1.4               pkgload_1.3.3              
#> [209] GenomeInfoDb_1.36.4         ipred_0.9-14               
#> [211] nloptr_2.0.3                DelayedArray_0.26.7        
#> [213] tidyselect_1.2.0            vipor_0.4.5                
#> [215] htmlTable_2.4.2             ggforce_0.4.1              
#> [217] CytoDx_1.20.0               car_3.1-2                  
#> [219] future_1.33.0               ModelMetrics_1.2.2.2       
#> [221] munsell_0.5.0               laeken_0.5.2               
#> [223] data.table_1.14.8           htmlwidgets_1.6.2          
#> [225] ComplexHeatmap_2.16.0       RColorBrewer_1.1-3         
#> [227] rlang_1.1.1                 remotes_2.4.2.1            
#> [229] colorRamps_2.3.1            Cairo_1.6-1                
#> [231] ggnewscale_0.4.9            fansi_1.0.5                
#> [233] hardhat_1.3.0               beeswarm_0.4.0             
#> [235] prodlim_2023.08.28
```
