# Supplementary material for: Unveiling the power of high-dimensional cytometry data with cyCONDOR
Source: Nat Commun. 2024 Dec 19;15:10702. doi: 10.1038/s41467-024-55179-w (PMC11659560; doi:10.1038/s41467-024-55179-w)
Supplement: Supplementary file 19 — Supplementary Data 17 [file 41467_2024_55179_MOESM19_ESM.html]

Supplementary Data 17: reproducibility data for Figure 5 - Differential Analysis


# Supplementary Data 17: reproducibility data for Figure 5 - Differential Analysis

```
library(cyCONDOR)
library(ggplot2)
library(ggsci)
library(dplyr)
library(ggrastr)
library(ggrastr)
library(ggpubr)
```

# Loading the data

```
condor <- prep_fcd(data_path = "/home/user/data/Data/Figure 2 - Example Workflow/data/FC/", 
                    max_cell = 10000, 
                    useCSV = FALSE, 
                    transformation = "auto_logi", 
                    remove_param = c("FSC-H", "SSC-H", "FSC-W", "SSC-W", "Time", "InFile", "live_dead"), 
                    anno_table = "/home/user/data/Data/Figure 2 - Example Workflow/data/FC_metadata.csv", 
                    filename_col = "filename",
                    seed = 91, 
                    verbose = TRUE)
#> [1] "Start reading the data"
#> [1] "Loading file 1 out of 6"
#> [1] "Loading file 2 out of 6"
#> [1] "Loading file 3 out of 6"
#> [1] "Loading file 4 out of 6"
#> [1] "Loading file 5 out of 6"
#> [1] "Loading file 6 out of 6"
#> [1] "Start transforming the data"
#> [1] "FSC-A w= 0 t= 189452.890625"
#> [1] "SSC-A w= 0 t= 159321.1875"
#> [1] "CD38 w= 1.08204990999969 t= 14864.46875"
#> [1] "CD8 w= 1.39647877544147 t= 12298.8408203125"
#> [1] "CD195 (CCR5) w= 1.47449646804538 t= 9324.6044921875"
#> [1] "CD94 (KLRD1) w= 1.08644854094052 t= 67681.4140625"
#> [1] "CD45RA w= 0.624871054871812 t= 188189.21875"
#> [1] "HLA-DR w= 0.929461421442766 t= 47207.3984375"
#> [1] "CD56 w= 1.06132042573662 t= 40519.19921875"
#> [1] "CD127 (IL7RA) w= 1.57275952764248 t= 5211.52490234375"
#> [1] "CD14 w= 1.20279949135721 t= 20560.888671875"
#> [1] "CD64 w= 0.945532824421731 t= 339462.875"
#> [1] "CD4 w= 1.00703642192255 t= 95269.0078125"
#> [1] "IgD w= 1.02627346790408 t= 77893.9609375"
#> [1] "CD19 w= 0.87097003245726 t= 196933.3125"
#> [1] "CD16 w= 0.834816694638715 t= 265510.25"
#> [1] "CD32 w= 0.77458993281154 t= 137295.5625"
#> [1] "CD197 (CCR7) w= 1.06501374238218 t= 28634.5234375"
#> [1] "CD20 w= 1.15174790062917 t= 42374.49609375"
#> [1] "CD27 w= 1.25795116181412 t= 27285.08984375"
#> [1] "CD15 w= 1.29238880617719 t= 52487.171875"
#> [1] "PD-1 w= 1.91214147798757 t= 3227.24658203125"
#> [1] "CD3 w= 1.22361754479372 t= 36392.27734375"
#> [1] "CD57 w= 0.392745042488233 t= 316672.84375"
#> [1] "CD25 w= 1.01070056777946 t= 21352.48828125"
#> [1] "CD123 (IL3RA) w= 1.12760875584084 t= 66552.2265625"
#> [1] "CD13 w= 1.06898469845033 t= 101909.6875"
#> [1] "CD11c w= 1.00050229943375 t= 50178.70703125"
```

```
class(condor)
#> [1] "flow_cytometry_dataframe"
```

# Dimensionality Reduction

## PCA

```
condor <- runPCA(fcd = condor, 
                 data_slot = "orig", 
                 seed = 91)
```

## UMAP

```
condor <- runUMAP(fcd = condor, 
                  input_type = "pca", 
                  data_slot = "orig", 
                  seed = 91)
```

# Clustering

## Phenograph

```
condor <- runPhenograph(fcd = condor, 
                        input_type = "pca", 
                        data_slot = "orig", 
                        k = 60, 
                        seed = 91)
#> Run Rphenograph starts:
#>   -Input data of 59049 rows and 28 columns
#>   -k is set to 60
#>   Finding nearest neighbors...DONE ~ 39.775 s
#>   Compute jaccard coefficient between nearest-neighbor sets...
#> Presorting knn...
#> presorting DONE ~ 2.222 s
#>   Start jaccard
#> DONE ~ 3.093 s
#>   Build undirected graph from the weighted links...DONE ~ 2.197 s
#>   Run louvain clustering on the graph ...DONE ~ 10.221 s
#> Run Rphenograph DONE, totally takes 55.286s.
#>   Return a community class
#>   -Modularity value: 0.8749651 
#>   -Number of clusters: 25
```

```
plot_dim_red(fcd = condor, 
             expr_slot = "orig", 
             reduction_method = "umap", 
             reduction_slot = "pca_orig", 
             cluster_slot = "phenograph_pca_orig_k_60",
             param = "Phenograph", 
             order = T, 
             title = "Figure S7b", 
             facet_by_variable = FALSE, 
             raster = TRUE)
```

# Metaclustering

```
condor <- metaclustering(fcd = condor, 
                         cluster_slot = "phenograph_pca_orig_k_60", 
                         cluster_var = "Phenograph", 
                         cluster_var_new = "metaclusters", 
                         metaclusters = c("1" = "Classical Monocytes", 
                                          "2" = "CD4", 
                                          "3" = "CD8", 
                                          "4" = "Nk dim", 
                                          "5" = "CD8", 
                                          "6" = "Classical Monocytes", 
                                          "7" = "Unconventional T cells", 
                                          "8" = "CD4", 
                                          "9" = "CD16+ Monocytes", 
                                          "10" = "CD4", 
                                          "11" = "Classical Monocytes", 
                                          "12" = "CD8", 
                                          "13" = "CD8",
                                          "14" = "NK bright",
                                          "15" = "CD8",
                                          "16" = "CD4",
                                          "17" = "B cells",
                                          "18" = "Unconventional T cells",
                                          "19" = "Classical Monocytes",
                                          "20" = "pDCs",
                                          "21" = "CD8",
                                          "22" = "Basophils",
                                          "23" = "Mixed",
                                          "24" = "B cells",
                                          "25" = "NK bright"))
#>    cluster            metacluster
#> 1        1    Classical Monocytes
#> 2        2                    CD4
#> 3        3                    CD8
#> 4        4                 Nk dim
#> 5        5                    CD8
#> 6        6    Classical Monocytes
#> 7        7 Unconventional T cells
#> 8        8                    CD4
#> 9        9        CD16+ Monocytes
#> 10      10                    CD4
#> 11      11    Classical Monocytes
#> 12      12                    CD8
#> 13      13                    CD8
#> 14      14              NK bright
#> 15      15                    CD8
#> 16      16                    CD4
#> 17      17                B cells
#> 18      18 Unconventional T cells
#> 19      19    Classical Monocytes
#> 20      20                   pDCs
#> 21      21                    CD8
#> 22      22              Basophils
#> 23      23                  Mixed
#> 24      24                B cells
#> 25      25              NK bright
```

```
plot_dim_red(fcd = condor, 
             expr_slot = "orig", 
             reduction_method = "umap", 
             reduction_slot = "pca_orig", 
             cluster_slot = "phenograph_pca_orig_k_60",
             param = "metaclusters", 
             order = T, 
             title = "Figure 5b", 
             facet_by_variable = FALSE, 
             raster = TRUE)
```

# Differential reppresentation

```
plot_confusion_HM(fcd = condor, 
                  cluster_slot = "phenograph_pca_orig_k_60", 
                  cluster_var = "Phenograph", 
                  group_var = "group", 
                  size = 30, 
                  title = "Figure S7c - Confusion Matrix - clusters")
```

```
plot_confusion_HM(fcd = condor, 
                  cluster_slot = "phenograph_pca_orig_k_60", 
                  cluster_var = "metaclusters", 
                  group_var = "group", 
                  size = 30, 
                  title = "Figure 5c - Confusion Matrix - metaclusters")
```

```
plot_frequency_barplot(fcd = condor,
                       cluster_slot = "phenograph_pca_orig_k_60",
                       cluster_var = "Phenograph",
                       group_var = "group",
                       facet_var = "group", 
                       title = "Figure S7d - Frequency clusters")
```

```
plot_frequency_barplot(fcd = condor,
                       cluster_slot = "phenograph_pca_orig_k_60",
                       cluster_var = "metaclusters",
                       group_var = "group",
                       facet_var = "group", 
                       title = "Figure 5d - Frequency clusters")
```

```
# Figure S7e
# factor levels can be used to influence plotting order of groupes on the x-axis
condor$anno$cell_anno$group<-factor(condor$anno$cell_anno$group, levels=c("ctrl","pat"))

# assign output to object plots
plots <- plot_frequency_boxplot(fcd = condor,
                                cluster_slot = "phenograph_pca_orig_k_60", 
                                cluster_var = "Phenograph",
                                sample_var = "sample_ID", 
                                group_var = "group", 
                                numeric = F,
                                color_palette = c("pat"="#F15A29","ctrl"="#92278F"))

# the list of plots can be summarized to one graph, by using e.g. the cowplot package.
cowplot::plot_grid(plotlist = plots, nrow = 4)
```

```
#ggsave("/home/user/data/Figures/Figure 5/raw/new/Fig_s7e.pdf", width = 12, height = 12)
```

```
# Figure 5e
# factor levels can be used to influence plotting order of groupes on the x-axis
condor$anno$cell_anno$group<-factor(condor$anno$cell_anno$group, levels=c("ctrl","pat"))

# assign output to object plots
plots <- plot_frequency_boxplot(fcd = condor,
                                cluster_slot = "phenograph_pca_orig_k_60", 
                                cluster_var = "metaclusters",
                                sample_var = "sample_ID", 
                                group_var = "group", 
                                numeric = F,
                                color_palette = c("pat"="#F15A29","ctrl"="#92278F"))

# the list of plots can be summarized to one graph, by using e.g. the cowplot package.
cowplot::plot_grid(plotlist = plots, nrow = 4)
```

```
#ggsave("/home/user/data/Figures/Figure 5/raw/new/Fig_5e.pdf", width = 5, height = 12)
```

```
results_ttest_metacluster <- frequency_t_test(fcd = condor,
                                              cluster_slot = "phenograph_pca_orig_k_60", 
                                              cluster_var = "metaclusters",
                                              sample_var = "sample_ID", 
                                              group_var = "group",
                                              paired_test = F,
                                              p.adjust.method = "bonferroni",
                                              numeric = F)

results_ttest_metacluster
#> # A tibble: 11 × 13
#>    cluster        .y.   group1 group2    n1    n2 statistic    df       p  p.adj
#>    <fct>          <chr> <chr>  <chr>  <int> <int>     <dbl> <dbl>   <dbl>  <dbl>
#>  1 B cells        value ctrl   pat        3     3     2.01   2.67 0.149   1     
#>  2 Basophils      value ctrl   pat        3     3     2.67   2.43 0.0951  1     
#>  3 CD16+ Monocyt… value ctrl   pat        3     3    -1.36   3.58 0.252   1     
#>  4 CD4            value ctrl   pat        3     3     3.59   3.16 0.0341  0.375 
#>  5 CD8            value ctrl   pat        3     3    -3.73   3.27 0.0289  0.318 
#>  6 Classical Mon… value ctrl   pat        3     3    -2.03   2.27 0.164   1     
#>  7 Mixed          value ctrl   pat        3     3     0.713  2.55 0.536   1     
#>  8 NK bright      value ctrl   pat        3     3     0.411  2.05 0.72    1     
#>  9 Nk dim         value ctrl   pat        3     3     0.522  3.75 0.631   1     
#> 10 pDCs           value ctrl   pat        3     3     2.62   3.84 0.0615  0.676 
#> 11 Unconventiona… value ctrl   pat        3     3   -12.7    2.17 0.00447 0.0492
#> # ℹ 3 more variables: p.adj.signif <chr>, p.adj_method <chr>,
#> #   applied_test <chr>

#write.csv(results_ttest_metacluster, file = "../../Tables/240706_Table2_DA_cyCONDOR_metaclusters.csv")
```

```
results_ttest_cluster <- frequency_t_test(fcd = condor,
                                          cluster_slot = "phenograph_pca_orig_k_60", 
                                          cluster_var = "Phenograph",
                                          sample_var = "sample_ID", 
                                          group_var = "group",
                                          paired_test = F,
                                          p.adjust.method = "bonferroni",
                                          numeric = F)

results_ttest_cluster
#> # A tibble: 25 × 13
#>    cluster .y.   group1 group2    n1    n2 statistic    df     p p.adj
#>    <fct>   <chr> <chr>  <chr>  <int> <int>     <dbl> <dbl> <dbl> <dbl>
#>  1 1       value ctrl   pat        3     3    -1.22   2.18 0.338     1
#>  2 10      value ctrl   pat        3     3     1.53   3.15 0.219     1
#>  3 11      value ctrl   pat        3     3    -0.633  3.09 0.571     1
#>  4 12      value ctrl   pat        3     3     1.36   3.97 0.247     1
#>  5 13      value ctrl   pat        3     3     1.07   2.00 0.396     1
#>  6 14      value ctrl   pat        3     3     0.648  2.09 0.581     1
#>  7 15      value ctrl   pat        3     3    -2.55   2.01 0.125     1
#>  8 16      value ctrl   pat        3     3     0.314  3.66 0.771     1
#>  9 17      value ctrl   pat        3     3     2.24   2.79 0.118     1
#> 10 18      value ctrl   pat        3     3    -1.10   2.00 0.387     1
#> # ℹ 15 more rows
#> # ℹ 3 more variables: p.adj.signif <chr>, p.adj_method <chr>,
#> #   applied_test <chr>

#write.csv(results_ttest_cluster, file = "../../Tables/240706_Table1_DA_cyCONDOR_clusters.csv")
```

# Differential expression

```
plot_marker_group_HM(fcd = condor,
                     expr_slot = "orig",
                     marker_to_exclude = c("FSC-A","SSC-A"),
                     cluster_slot = "phenograph_pca_orig_k_60",
                     cluster_var = "Phenograph",
                     group_var = "group", 
                     title = "Figure S8a")
```

```
plot_marker_group_HM(fcd = condor,
                     expr_slot = "orig",
                     marker_to_exclude = c("FSC-A","SSC-A"),
                     cluster_slot = "phenograph_pca_orig_k_60",
                     cluster_var = "metaclusters",
                     group_var = "group", 
                     title = "Figure 5f")
```

# diffcyt

## Clusters

### Differential aboundance

```
condor_se<-prepInputDiffcyt(fcd = condor,
                            cluster_slot = "phenograph_pca_orig_k_60",
                            cluster_var = "Phenograph",
                            sample_var = "sample_ID",
                            meta_vars = c("expfcs_filename","group"),
                            marker_state = NULL,
                            marker_type = NULL)
```

```
condor_se
#> class: SummarizedExperiment 
#> dim: 59049 28 
#> metadata(2): experiment_info n_cells
#> assays(1): exprs
#> rownames(59049): ID10.fcs_1 ID10.fcs_2 ... ID8.fcs_59048 ID8.fcs_59049
#> rowData names(4): sample_id expfcs_filename group cluster_id
#> colnames(28): FSC-A SSC-A ... CD13 CD11c
#> colData names(3): channel_name marker_name marker_class
```

```
# calculate cell counts
d_counts <- diffcyt::calcCounts(condor_se)

# inspect object
d_counts
#> class: SummarizedExperiment 
#> dim: 25 6 
#> metadata(0):
#> assays(1): counts
#> rownames(25): 1 2 ... 24 25
#> rowData names(2): cluster_id n_cells
#> colnames(6): ID10 ID3 ... ID7 ID8
#> colData names(3): sample_id expfcs_filename group
```

```
# inspect counts
SummarizedExperiment::assays(d_counts)$counts
#>    ID10  ID3  ID5  ID6  ID7  ID8
#> 1  2378 1572 1453 1007 1266 2375
#> 2   569  993  933  191 1475 1498
#> 3   607 1008  811  707  283  656
#> 4   641  825  517 1242 1490   79
#> 5  1912  140  615 1053   68 1623
#> 6   221  108  119   77  129   85
#> 7   493   20   29  245    7  579
#> 8   564 1837 1614  535 1572  613
#> 9   342  350  219  494  143  255
#> 10  365  500  485  263  875  533
#> 11  541  524  436  875  714  492
#> 12  228  360  215  290  465   92
#> 13  233  112  512  210  509  241
#> 14  315  446  493  583  506  243
#> 15  211    2    8   32    0  206
#> 16  101  324  202  312  126  114
#> 17  103  352  430  179  171   86
#> 18    3    8    5  183    1   19
#> 19   44  134  364  143   42   56
#> 20   24   80   66   30   55   54
#> 21   75  157  109  187   25   41
#> 22   13   80   35   24   59   25
#> 23    7   48  248   90   10   31
#> 24    8    2   63   13    0    0
#> 25    2   18   19   84    9    4
```

```
# Calculate cluster medians
d_medians <- diffcyt::calcMedians(condor_se)

#inspect medians
d_medians
#> class: SummarizedExperiment 
#> dim: 25 6 
#> metadata(2): id_type_markers id_state_markers
#> assays(28): FSC-A SSC-A ... CD13 CD11c
#> rownames(25): 1 2 ... 24 25
#> rowData names(1): cluster_id
#> colnames(6): ID10 ID3 ... ID7 ID8
#> colData names(3): sample_id expfcs_filename group
```

```
experiment_info<-condor_se@metadata$experiment_info

design <- diffcyt::createDesignMatrix(experiment_info,
                                      cols_design = c("group"))
design
#>      (Intercept) grouppat
#> ID10           1        1
#> ID3            1        0
#> ID5            1        0
#> ID6            1        1
#> ID7            1        0
#> ID8            1        1
#> attr(,"assign")
#> [1] 0 1
#> attr(,"contrasts")
#> attr(,"contrasts")$group
#> [1] "contr.treatment"
```

```
# Create contrast matrix
contrast <- diffcyt::createContrast(c(0, 1))

# check
nrow(contrast) == ncol(design)
#> [1] TRUE

data.frame(parameters = colnames(design), contrast)
#>    parameters contrast
#> 1 (Intercept)        0
#> 2    grouppat        1
```

```
# perform differential abundance test
res_DA <- diffcyt::testDA_edgeR(d_counts, design, contrast)
```

```
# table of results ordered by increasing adjusted p-value
cluster_DA_results <- as.data.frame(diffcyt::topTable(res_DA, all = TRUE))

cluster_DA_results
#>    cluster_id        p_val        p_adj
#> 7           7 2.079442e-08 5.198606e-07
#> 15         15 3.394726e-05 4.243408e-04
#> 8           8 2.488831e-03 2.074026e-02
#> 5           5 7.096350e-03 4.435219e-02
#> 18         18 9.415567e-03 4.707783e-02
#> 22         22 3.020419e-02 1.258508e-01
#> 17         17 5.127675e-02 1.831312e-01
#> 20         20 1.591209e-01 4.972528e-01
#> 1           1 4.290172e-01 6.319465e-01
#> 9           9 2.831441e-01 6.319465e-01
#> 10         10 2.689944e-01 6.319465e-01
#> 12         12 3.101163e-01 6.319465e-01
#> 13         13 3.420306e-01 6.319465e-01
#> 19         19 2.565443e-01 6.319465e-01
#> 23         23 3.612324e-01 6.319465e-01
#> 24         24 4.297236e-01 6.319465e-01
#> 25         25 4.011066e-01 6.319465e-01
#> 2           2 4.730098e-01 6.569581e-01
#> 4           4 6.581336e-01 7.864376e-01
#> 11         11 6.575238e-01 7.864376e-01
#> 14         14 6.606076e-01 7.864376e-01
#> 16         16 7.668253e-01 8.713924e-01
#> 6           6 8.300361e-01 9.022131e-01
#> 21         21 8.703053e-01 9.065680e-01
#> 3           3 9.509044e-01 9.509044e-01

#write.csv(cluster_DA_results, file = "../../Tables/240706_Table3_DA_diffcyt_clusters.csv")
```

## Metaclusters

### Differential aboundance

```
condor_se<-prepInputDiffcyt(fcd = condor,
                            cluster_slot = "phenograph_pca_orig_k_60",
                            cluster_var = "metaclusters",
                            sample_var = "sample_ID",
                            meta_vars = c("expfcs_filename","group"),
                            marker_state = NULL,
                            marker_type = NULL)
```

```
condor_se
#> class: SummarizedExperiment 
#> dim: 59049 28 
#> metadata(2): experiment_info n_cells
#> assays(1): exprs
#> rownames(59049): ID10.fcs_1 ID10.fcs_2 ... ID8.fcs_59048 ID8.fcs_59049
#> rowData names(4): sample_id expfcs_filename group cluster_id
#> colnames(28): FSC-A SSC-A ... CD13 CD11c
#> colData names(3): channel_name marker_name marker_class
```

```
# calculate cell counts
d_counts <- diffcyt::calcCounts(condor_se)

# inspect object
d_counts
#> class: SummarizedExperiment 
#> dim: 11 6 
#> metadata(0):
#> assays(1): counts
#> rownames(11): Classical Monocytes CD4 ... Basophils Mixed
#> rowData names(2): cluster_id n_cells
#> colnames(6): ID10 ID3 ... ID7 ID8
#> colData names(3): sample_id expfcs_filename group
```

```
# inspect counts
SummarizedExperiment::assays(d_counts)$counts
#>                        ID10  ID3  ID5  ID6  ID7  ID8
#> Classical Monocytes    3184 2338 2372 2102 2151 3008
#> CD4                    1599 3654 3234 1301 4048 2758
#> CD8                    3266 1779 2270 2479 1350 2859
#> Nk dim                  641  825  517 1242 1490   79
#> Unconventional T cells  496   28   34  428    8  598
#> CD16+ Monocytes         342  350  219  494  143  255
#> NK bright               317  464  512  667  515  247
#> B cells                 111  354  493  192  171   86
#> pDCs                     24   80   66   30   55   54
#> Basophils                13   80   35   24   59   25
#> Mixed                     7   48  248   90   10   31
```

```
# Calculate cluster medians
d_medians <- diffcyt::calcMedians(condor_se)

#inspect medians
d_medians
#> class: SummarizedExperiment 
#> dim: 11 6 
#> metadata(2): id_type_markers id_state_markers
#> assays(28): FSC-A SSC-A ... CD13 CD11c
#> rownames(11): Classical Monocytes CD4 ... Basophils Mixed
#> rowData names(1): cluster_id
#> colnames(6): ID10 ID3 ... ID7 ID8
#> colData names(3): sample_id expfcs_filename group
```

```
# inspect medians
SummarizedExperiment::assays(d_medians)$CD57
#>                             ID10       ID3       ID5       ID6       ID7
#> Classical Monocytes    1.2342792 0.9569836 0.8784949 1.1214760 0.9437813
#> CD4                    0.8636581 0.7256709 0.6477634 0.8112442 0.6627282
#> CD8                    3.2863153 0.8976232 0.9852229 2.3968618 0.7624646
#> Nk dim                 3.3696450 3.5735292 3.0539440 3.4984811 3.3147029
#> Unconventional T cells 3.8024406 2.7093433 2.7061293 3.3138678 3.3122278
#> CD16+ Monocytes        0.7645921 0.7576529 0.7646734 0.9454071 0.5967356
#> NK bright              0.7663569 0.8889427 0.7204651 0.8904777 0.7738680
#> B cells                0.6241813 0.3338990 0.4309125 0.5653783 0.3539324
#> pDCs                   0.9298423 1.0022037 0.7510136 0.9939891 0.7913450
#> Basophils              1.2820025 1.0921873 0.9382373 1.2456938 1.3153434
#> Mixed                  0.6692602 0.6345080 0.6300146 0.8673538 1.0005692
#>                              ID8
#> Classical Monocytes    1.2888429
#> CD4                    0.7754308
#> CD8                    3.2786161
#> Nk dim                 2.9819875
#> Unconventional T cells 3.7977096
#> CD16+ Monocytes        0.8756224
#> NK bright              1.0096297
#> B cells                0.4325274
#> pDCs                   1.0707367
#> Basophils              1.2879905
#> Mixed                  0.6692396
```

```
experiment_info<-condor_se@metadata$experiment_info

design <- diffcyt::createDesignMatrix(experiment_info,
                                      cols_design = c("group"))
design
#>      (Intercept) grouppat
#> ID10           1        1
#> ID3            1        0
#> ID5            1        0
#> ID6            1        1
#> ID7            1        0
#> ID8            1        1
#> attr(,"assign")
#> [1] 0 1
#> attr(,"contrasts")
#> attr(,"contrasts")$group
#> [1] "contr.treatment"
```

```
# Create contrast matrix
contrast <- diffcyt::createContrast(c(0, 1))

# check
nrow(contrast) == ncol(design)
#> [1] TRUE

data.frame(parameters = colnames(design), contrast)
#>    parameters contrast
#> 1 (Intercept)        0
#> 2    grouppat        1
```

```
# perform differential abundance test
res_DA <- diffcyt::testDA_edgeR(d_counts, design, contrast)
```

```
# table of results ordered by increasing adjusted p-value
metacluster_DA_results <- as.data.frame(diffcyt::topTable(res_DA, all = TRUE))

metacluster_DA_results
#>                                    cluster_id        p_val        p_adj
#> Unconventional T cells Unconventional T cells 3.340550e-11 3.674605e-10
#> Basophils                           Basophils 1.375695e-02 7.566325e-02
#> B cells                               B cells 3.357648e-02 1.231138e-01
#> CD4                                       CD4 5.331948e-02 1.466286e-01
#> CD8                                       CD8 1.046075e-01 1.927530e-01
#> pDCs                                     pDCs 1.051380e-01 1.927530e-01
#> CD16+ Monocytes               CD16+ Monocytes 2.237650e-01 3.516308e-01
#> Mixed                                   Mixed 3.370247e-01 4.634089e-01
#> Classical Monocytes       Classical Monocytes 4.519686e-01 5.524061e-01
#> Nk dim                                 Nk dim 6.362066e-01 6.998273e-01
#> NK bright                           NK bright 7.241735e-01 7.241735e-01

#write.csv(metacluster_DA_results, file = "../../Tables/240706_Table4_DA_diffcyt_metaclusterclusters.csv")
```

### Differential expression

```
experiment_info<-condor_se@metadata$experiment_info

formula <- diffcyt::createFormula(experiment_info, cols_fixed = "group")

formula
#> $formula
#> y ~ group
#> <environment: 0x5652ce41bd68>
#> 
#> $data
#>      group
#> ID10   pat
#> ID3   ctrl
#> ID5   ctrl
#> ID6    pat
#> ID7   ctrl
#> ID8    pat
#> 
#> $random_terms
#> [1] FALSE
```

```
marker_info <- SummarizedExperiment::colData(condor_se) %>% as.data.frame()
marker_info
#>                channel_name   marker_name marker_class
#> FSC-A                 FSC-A         FSC-A         type
#> SSC-A                 SSC-A         SSC-A         type
#> CD38                   CD38          CD38         type
#> CD8                     CD8           CD8         type
#> CD195 (CCR5)   CD195 (CCR5)  CD195 (CCR5)         type
#> CD94 (KLRD1)   CD94 (KLRD1)  CD94 (KLRD1)         type
#> CD45RA               CD45RA        CD45RA         type
#> HLA-DR               HLA-DR        HLA-DR         type
#> CD56                   CD56          CD56         type
#> CD127 (IL7RA) CD127 (IL7RA) CD127 (IL7RA)         type
#> CD14                   CD14          CD14         type
#> CD64                   CD64          CD64         type
#> CD4                     CD4           CD4         type
#> IgD                     IgD           IgD         type
#> CD19                   CD19          CD19         type
#> CD16                   CD16          CD16         type
#> CD32                   CD32          CD32         type
#> CD197 (CCR7)   CD197 (CCR7)  CD197 (CCR7)         type
#> CD20                   CD20          CD20         type
#> CD27                   CD27          CD27         type
#> CD15                   CD15          CD15         type
#> PD-1                   PD-1          PD-1         type
#> CD3                     CD3           CD3         type
#> CD57                   CD57          CD57         type
#> CD25                   CD25          CD25         type
#> CD123 (IL3RA) CD123 (IL3RA) CD123 (IL3RA)         type
#> CD13                   CD13          CD13         type
#> CD11c                 CD11c         CD11c         type
```

```
#get logical vector of marker to be tested
markers_oi <- !names(SummarizedExperiment::assays(d_medians)) %in% c("SSC-A","FSC-A")

# perform differential state analysis
res_DS <- diffcyt::testDS_LMM(d_counts, d_medians, formula, contrast,
                                markers_to_test = markers_oi)
```

```
res_metacluster <-as.data.frame(diffcyt::topTable(res_DS, all = TRUE))

res_metacluster[1:10,]
#>                                    cluster_id     marker_id       p_val
#> CD8                                       CD8  CD94 (KLRD1) 0.003455261
#> CD8.1                                     CD8          CD57 0.001970022
#> Classical Monocytes       Classical Monocytes          CD57 0.005718462
#> CD8.2                                     CD8          CD27 0.009780213
#> Unconventional T cells Unconventional T cells CD123 (IL3RA) 0.009053205
#> pDCs                                     pDCs        CD45RA 0.018106991
#> Classical Monocytes.1     Classical Monocytes          CD27 0.014399514
#> CD4                                       CD4          CD57 0.017293975
#> Basophils                           Basophils CD123 (IL3RA) 0.013176378
#> CD8.3                                     CD8          CD56 0.029806043
#>                            p_adj
#> CD8                    0.4941023
#> CD8.1                  0.4941023
#> Classical Monocytes    0.5451600
#> CD8.2                  0.5594282
#> Unconventional T cells 0.5594282
#> pDCs                   0.5753999
#> Classical Monocytes.1  0.5753999
#> CD4                    0.5753999
#> Basophils              0.5753999
#> CD8.3                  0.7095779

#write.csv(res_metacluster, file = "../../Tables/240706_Table5_DE_diffcyt_metaclusters.csv")
```

## Figure s8b

```
plot_marker_boxplot(fcd = condor,
                    marker = c("CD94 (KLRD1)", "CD57"),
                    expr_slot ="orig",
                    cluster_slot = "phenograph_pca_orig_k_60",
                    cluster_var = "metaclusters",
                    cluster_to_show = "CD8",
                    facet_by_clustering = T,
                    group_var = "group", 
                    sample_var = "sample_ID",
                    fun = "median", 
                    color_palette = c("pat"="#F15A29","ctrl"="#92278F")) + theme(aspect.ratio = 1)
```

# Session Info

```
info <- sessionInfo()

info
#> R version 4.3.1 (2023-06-16)
#> Platform: x86_64-pc-linux-gnu (64-bit)
#> Running under: Ubuntu 22.04.3 LTS
#> 
#> Matrix products: default
#> BLAS:   /usr/lib/x86_64-linux-gnu/openblas-pthread/libblas.so.3 
#> LAPACK: /usr/lib/x86_64-linux-gnu/openblas-pthread/libopenblasp-r0.3.20.so;  LAPACK version 3.10.0
#> 
#> locale:
#>  [1] LC_CTYPE=en_US.UTF-8       LC_NUMERIC=C              
#>  [3] LC_TIME=en_US.UTF-8        LC_COLLATE=en_US.UTF-8    
#>  [5] LC_MONETARY=en_US.UTF-8    LC_MESSAGES=en_US.UTF-8   
#>  [7] LC_PAPER=en_US.UTF-8       LC_NAME=C                 
#>  [9] LC_ADDRESS=C               LC_TELEPHONE=C            
#> [11] LC_MEASUREMENT=en_US.UTF-8 LC_IDENTIFICATION=C       
#> 
#> time zone: Etc/UTC
#> tzcode source: system (glibc)
#> 
#> attached base packages:
#> [1] stats     graphics  grDevices utils     datasets  methods   base     
#> 
#> other attached packages:
#> [1] ggpubr_0.6.0   ggrastr_1.0.2  dplyr_1.1.3    ggsci_3.0.0    ggplot2_3.4.4 
#> [6] cyCONDOR_0.2.0
#> 
#> loaded via a namespace (and not attached):
#>   [1] IRanges_2.34.1              Rmisc_1.5.1                
#>   [3] urlchecker_1.0.1            nnet_7.3-19                
#>   [5] CytoNorm_2.0.1              TH.data_1.1-2              
#>   [7] vctrs_0.6.4                 digest_0.6.33              
#>   [9] png_0.1-8                   shape_1.4.6                
#>  [11] proxy_0.4-27                slingshot_2.8.0            
#>  [13] ggrepel_0.9.4               parallelly_1.36.0          
#>  [15] MASS_7.3-60                 reshape2_1.4.4             
#>  [17] httpuv_1.6.12               foreach_1.5.2              
#>  [19] BiocGenerics_0.46.0         withr_2.5.1                
#>  [21] xfun_0.40                   ellipsis_0.3.2             
#>  [23] survival_3.5-7              memoise_2.0.1              
#>  [25] hexbin_1.28.3               ggbeeswarm_0.7.2           
#>  [27] RProtoBufLib_2.12.1         princurve_2.1.6            
#>  [29] profvis_0.3.8               zoo_1.8-12                 
#>  [31] GlobalOptions_0.1.2         DEoptimR_1.1-3             
#>  [33] Formula_1.2-5               prettyunits_1.2.0          
#>  [35] promises_1.2.1              scatterplot3d_0.3-44       
#>  [37] rstatix_0.7.2               globals_0.16.2             
#>  [39] ps_1.7.5                    rstudioapi_0.15.0          
#>  [41] miniUI_0.1.1.1              generics_0.1.3             
#>  [43] ggcyto_1.28.1               base64enc_0.1-3            
#>  [45] processx_3.8.2              curl_5.1.0                 
#>  [47] S4Vectors_0.38.2            zlibbioc_1.46.0            
#>  [49] flowWorkspace_4.12.2        polyclip_1.10-6            
#>  [51] randomForest_4.7-1.1        GenomeInfoDbData_1.2.10    
#>  [53] RBGL_1.76.0                 ncdfFlow_2.46.0            
#>  [55] RcppEigen_0.3.3.9.4         xtable_1.8-4               
#>  [57] stringr_1.5.0               doParallel_1.0.17          
#>  [59] evaluate_0.22               S4Arrays_1.0.6             
#>  [61] hms_1.1.3                   glmnet_4.1-8               
#>  [63] GenomicRanges_1.52.1        irlba_2.3.5.1              
#>  [65] colorspace_2.1-0            harmony_1.1.0              
#>  [67] reticulate_1.34.0           readxl_1.4.3               
#>  [69] magrittr_2.0.3              lmtest_0.9-40              
#>  [71] readr_2.1.4                 Rgraphviz_2.44.0           
#>  [73] later_1.3.1                 lattice_0.22-5             
#>  [75] future.apply_1.11.0         robustbase_0.99-0          
#>  [77] XML_3.99-0.15               cowplot_1.1.1              
#>  [79] matrixStats_1.1.0           RcppAnnoy_0.0.21           
#>  [81] xts_0.13.1                  class_7.3-22               
#>  [83] Hmisc_5.1-1                 pillar_1.9.0               
#>  [85] nlme_3.1-163                iterators_1.0.14           
#>  [87] compiler_4.3.1              RSpectra_0.16-1            
#>  [89] stringi_1.7.12              gower_1.0.1                
#>  [91] minqa_1.2.6                 SummarizedExperiment_1.30.2
#>  [93] lubridate_1.9.3             devtools_2.4.5             
#>  [95] CytoML_2.12.0               plyr_1.8.9                 
#>  [97] crayon_1.5.2                abind_1.4-5                
#>  [99] locfit_1.5-9.8              sp_2.1-1                   
#> [101] sandwich_3.0-2              pcaMethods_1.92.0          
#> [103] codetools_0.2-19            multcomp_1.4-25            
#> [105] recipes_1.0.8               openssl_2.1.1              
#> [107] Rphenograph_0.99.1          TTR_0.24.3                 
#> [109] bslib_0.5.1                 e1071_1.7-13               
#> [111] destiny_3.14.0              GetoptLong_1.0.5           
#> [113] ggplot.multistats_1.0.0     mime_0.12                  
#> [115] splines_4.3.1               circlize_0.4.15            
#> [117] Rcpp_1.0.11                 sparseMatrixStats_1.12.2   
#> [119] cellranger_1.1.0            knitr_1.44                 
#> [121] utf8_1.2.4                  clue_0.3-65                
#> [123] lme4_1.1-35.1               fs_1.6.3                   
#> [125] listenv_0.9.0               checkmate_2.3.0            
#> [127] DelayedMatrixStats_1.22.6   pkgbuild_1.4.2             
#> [129] ggsignif_0.6.4              tibble_3.2.1               
#> [131] Matrix_1.6-1.1              rpart.plot_3.1.1           
#> [133] callr_3.7.3                 tzdb_0.4.0                 
#> [135] tweenr_2.0.2                pkgconfig_2.0.3            
#> [137] pheatmap_1.0.12             tools_4.3.1                
#> [139] cachem_1.0.8                smoother_1.1               
#> [141] fastmap_1.1.1               rmarkdown_2.25             
#> [143] scales_1.2.1                grid_4.3.1                 
#> [145] usethis_2.2.2               broom_1.0.5                
#> [147] sass_0.4.7                  graph_1.78.0               
#> [149] carData_3.0-5               RANN_2.6.1                 
#> [151] rpart_4.1.21                farver_2.1.1               
#> [153] yaml_2.3.7                  MatrixGenerics_1.12.3      
#> [155] foreign_0.8-85              ggthemes_4.2.4             
#> [157] cli_3.6.1                   purrr_1.0.2                
#> [159] stats4_4.3.1                lifecycle_1.0.3            
#> [161] uwot_0.1.16                 askpass_1.2.0              
#> [163] caret_6.0-94                Biobase_2.60.0             
#> [165] mvtnorm_1.2-3               lava_1.7.3                 
#> [167] sessioninfo_1.2.2           backports_1.4.1            
#> [169] cytolib_2.12.1              timechange_0.2.0           
#> [171] gtable_0.3.4                rjson_0.2.21               
#> [173] umap_0.2.10.0               ggridges_0.5.4             
#> [175] Rphenoannoy_0.1.0           parallel_4.3.1             
#> [177] pROC_1.18.5                 limma_3.56.2               
#> [179] jsonlite_1.8.7              edgeR_3.42.4               
#> [181] RcppHNSW_0.5.0              bitops_1.0-7               
#> [183] Rtsne_0.16                  FlowSOM_2.8.0              
#> [185] ranger_0.16.0               flowCore_2.12.2            
#> [187] jquerylib_0.1.4             timeDate_4022.108          
#> [189] shiny_1.7.5.1               ConsensusClusterPlus_1.64.0
#> [191] htmltools_0.5.6.1           diffcyt_1.20.0             
#> [193] glue_1.6.2                  XVector_0.40.0             
#> [195] VIM_6.2.2                   RCurl_1.98-1.13            
#> [197] gridExtra_2.3               boot_1.3-28.1              
#> [199] igraph_1.5.1                TrajectoryUtils_1.8.0      
#> [201] R6_2.5.1                    tidyr_1.3.0                
#> [203] SingleCellExperiment_1.22.0 labeling_0.4.3             
#> [205] vcd_1.4-11                  cluster_2.1.4              
#> [207] pkgload_1.3.3               GenomeInfoDb_1.36.4        
#> [209] ipred_0.9-14                nloptr_2.0.3               
#> [211] DelayedArray_0.26.7         tidyselect_1.2.0           
#> [213] vipor_0.4.5                 htmlTable_2.4.2            
#> [215] ggforce_0.4.1               CytoDx_1.20.0              
#> [217] car_3.1-2                   future_1.33.0              
#> [219] ModelMetrics_1.2.2.2        munsell_0.5.0              
#> [221] laeken_0.5.2                data.table_1.14.8          
#> [223] htmlwidgets_1.6.2           ComplexHeatmap_2.16.0      
#> [225] RColorBrewer_1.1-3          rlang_1.1.1                
#> [227] remotes_2.4.2.1             colorRamps_2.3.1           
#> [229] Cairo_1.6-1                 ggnewscale_0.4.9           
#> [231] fansi_1.0.5                 hardhat_1.3.0              
#> [233] beeswarm_0.4.0              prodlim_2023.08.28
```
