# Supplementary material for: Unveiling the power of high-dimensional cytometry data with cyCONDOR
Source: Nat Commun. 2024 Dec 19;15:10702. doi: 10.1038/s41467-024-55179-w (PMC11659560; doi:10.1038/s41467-024-55179-w)
Supplement: Supplementary file 20 — Supplementary Data 18 [file 41467_2024_55179_MOESM20_ESM.html]

Supplementary Data 18: reproducibility data for Figure 6 - Label transfer workflow


# Supplementary Data 18: reproducibility data for Figure 6 - Label transfer workflow

```
library(cyCONDOR)
library(ggpubr)
library(ggrastr)
library(pheatmap)
```

# Loading the data for training

```
condor_train <- prep_fcd(data_path = "./data_and_envs/fcs_train/", 
                        max_cell = 5000, 
                        useCSV = FALSE, 
                        transformation = "auto_logi", 
                        remove_param = c("FSC-H", "SSC-H", "FSC-W", "SSC-W", "Time", "live_dead"), 
                        anno_table = "./data_and_envs/metadata_train.csv", 
                        filename_col = "filename",
                        seed = 91, 
                        verbose = TRUE)
```

```
## [1] "Start reading the data"
## [1] "Loading file 1 out of 9"
## [1] "Loading file 2 out of 9"
## [1] "Loading file 3 out of 9"
## [1] "Loading file 4 out of 9"
## [1] "Loading file 5 out of 9"
## [1] "Loading file 6 out of 9"
## [1] "Loading file 7 out of 9"
## [1] "Loading file 8 out of 9"
## [1] "Loading file 9 out of 9"
## [1] "Start transforming the data"
## [1] "FSC-A w= 0 t= 189548.3125"
## [1] "SSC-A w= 0 t= 143017.28125"
## [1] "CD38 w= 1.00467092903809 t= 21098.90234375"
## [1] "CD8 w= 1.39087928377008 t= 13280.115234375"
## [1] "CD195 (CCR5) w= 1.47496968547118 t= 9324.6044921875"
## [1] "CD94 (KLRD1) w= 1.14024165714939 t= 52697.83984375"
## [1] "CD45RA w= 0.575559538942434 t= 188189.21875"
## [1] "HLA-DR w= 0.906457376716275 t= 52268.72265625"
## [1] "CD56 w= 1.08251168835056 t= 38711.34375"
## [1] "CD127 (IL7RA) w= 1.24594483351245 t= 22799.275390625"
## [1] "CD14 w= 1.20856525032223 t= 19707.974609375"
## [1] "CD64 w= 1.46255388991963 t= 30131.552734375"
## [1] "CD4 w= 1.11074922553137 t= 60661.015625"
## [1] "IgD w= 1.01328989695129 t= 86759.4296875"
## [1] "CD19 w= 1.1442639974397 t= 56064.35546875"
## [1] "CD16 w= 0.907155415750036 t= 183188.46875"
## [1] "CD32 w= 1.14295376712075 t= 24696.62109375"
## [1] "CD197 (CCR7) w= 1.06373634722298 t= 28634.5234375"
## [1] "CD20 w= 1.07250534509397 t= 59596.2265625"
## [1] "CD27 w= 1.31710110098513 t= 22037.9765625"
## [1] "CD15 w= 1.29414575567539 t= 52487.171875"
## [1] "PD-1 w= 1.91283109324646 t= 3218.92749023438"
## [1] "CD3 w= 1.2093241717371 t= 36624.5625"
## [1] "CD57 w= 0.36295690399458 t= 386989.96875"
## [1] "CD25 w= 1.05414262118344 t= 16987.53515625"
## [1] "CD123 (IL3RA) w= 1.12703027334259 t= 66552.2265625"
## [1] "CD13 w= 1.06214617659589 t= 105455.2265625"
## [1] "CD11c w= 0.961656743322294 t= 61599.1171875"
```

```
class(condor_train)
```

```
## [1] "flow_cytometry_dataframe"
```

```
condor_train$anno$cell_anno$group <- "train"
```

# Loading the data

```
condor_test <- prep_fcd(data_path = "./data_and_envs/fcs_test/", 
                        max_cell = 10000, 
                        useCSV = FALSE, 
                        transformation = "auto_logi", 
                        remove_param = c("FSC-H", "SSC-H", "FSC-W", "SSC-W", "Time", "live_dead"), 
                        anno_table = "./data_and_envs/metadata_test.csv", 
                        filename_col = "filename",
                        seed = 91)
```

```
class(condor_test)
```

```
## [1] "flow_cytometry_dataframe"
```

```
condor_test$anno$cell_anno$group <- "test"
```

# UMAP Projection

## Run UMAP keeping the model

```
condor_train <- runUMAP(fcd = condor_train, 
                        input_type = "expr", 
                        data_slot = "orig", 
                        seed = 91, 
                        nThreads = 4, 
                        ret_model = TRUE)
```

## Add data to the embedding

```
condor_test <- learnUMAP(fcd = condor_test, 
                         input_type = "expr", 
                         data_slot = "orig", 
                         fcd_model = condor_train, 
                         nEpochs = 100, 
                         seed = 91, 
                         nThreads = 4)
```

# Train a classifier for the label transfer

```
condor_train <- runPhenograph(fcd = condor_train, 
                              input_type = "expr", 
                              data_slot = "orig", 
                              k = 150, 
                              seed = 91)
```

```
## Run Rphenograph starts:
##   -Input data of 45000 rows and 28 columns
##   -k is set to 150
```

```
##   Finding nearest neighbors...DONE ~ 47.577 s
##   Compute jaccard coefficient between nearest-neighbor sets...
```

```
## Presorting knn...
```

```
## presorting DONE ~ 2.016 s
##   Start jaccard
## DONE ~ 38.978 s
##   Build undirected graph from the weighted links...DONE ~ 3.88 s
##   Run louvain clustering on the graph ...DONE ~ 25.822 s
```

```
## Run Rphenograph DONE, totally takes 116.257s.
```

```
##   Return a community class
##   -Modularity value: 0.846826 
##   -Number of clusters: 17
```

## Visualization

```
plot_dim_red(fcd = condor_train, 
             expr_slot = "orig", 
             reduction_method = "umap", 
             reduction_slot = "expr_orig", 
             cluster_slot = "phenograph_expr_orig_k_150",
             param = "Phenograph", 
             order = T, 
             title = "Figure S9b -UMAP on the training dataset, Phenograph clusters", 
             facet_by_variable = FALSE, 
             raster = TRUE)
```

# Metaclustering

```
condor_train <- metaclustering(fcd = condor_train, 
                               cluster_slot = "phenograph_expr_orig_k_150", 
                               cluster_var = "Phenograph", 
                               cluster_var_new = "metaclusters", 
                               metaclusters = c("1" = "CD8 T", 
                                                "2" = "Non-classical Monocytes", 
                                                "3" = "Classical Monocytes", 
                                                "4" = "Classical Monocytes", 
                                                "5" = "CD8 T", 
                                                "6" = "CD4 T", 
                                                "7" = "Classical Monocytes", 
                                                "8" = "CD4 T", 
                                                "9" = "CD8 T", 
                                                "10" = "CD4 T", 
                                                "11" = "CD4 T", 
                                                "12" = "NK", 
                                                "13" = "NK",
                                                "14" = "B",
                                                "15" = "CD4 T",
                                                "16" = "Classical Monocytes",
                                                "17" = "pDC"))
```

```
##    cluster             metacluster
## 1        1                   CD8 T
## 2        2 Non-classical Monocytes
## 3        3     Classical Monocytes
## 4        4     Classical Monocytes
## 5        5                   CD8 T
## 6        6                   CD4 T
## 7        7     Classical Monocytes
## 8        8                   CD4 T
## 9        9                   CD8 T
## 10      10                   CD4 T
## 11      11                   CD4 T
## 12      12                      NK
## 13      13                      NK
## 14      14                       B
## 15      15                   CD4 T
## 16      16     Classical Monocytes
## 17      17                     pDC
```

```
plot_dim_red(fcd = condor_train, 
             expr_slot = "orig", 
             reduction_method = "umap", 
             reduction_slot = "expr_orig", 
             cluster_slot = "phenograph_expr_orig_k_150",
             param = "metaclusters", 
             order = T, 
             title = "Figure 6b - UMAP on the training dataset, metaclusters", 
             facet_by_variable = FALSE, 
             raster = TRUE)
```

# Label transfer

## Train label trasfer kNN clussifier

```
condor_train <- train_transfer_model(fcd = condor_train, 
                                     data_slot = "orig", 
                                     input_type = "expr", 
                                     cluster_slot = "phenograph_expr_orig_k_150", 
                                     cluster_var = "metaclusters",
                                     method = "knn", 
                                     tuneLength = 5, 
                                     trControl = caret::trainControl(method = "cv"), 
                                     seed = 91)
```

```
## Loading required package: lattice
```

```
## 
## Attaching package: 'caret'
```

```
## The following object is masked from 'package:cyCONDOR':
## 
##     confusionMatrix
```

```
condor_train$extras$lt_model$performance_plot + ggtitle("Figure S9c_meta - kNN accuracy")
```

```
# Figure S9e - kNN importance

condor_train$extras$lt_model$features_plot
```

## Trasfer lables on the new data

```
condor_test <- predict_labels(fcd = condor_test, 
                              data_slot = "orig", 
                              input_type = "expr", 
                              fcd_model = condor_train, 
                              label = "label_pred", 
                              seed = 91)
```

## Train label transfer kNN classifier - cluster level

```
condor_train_fine <- train_transfer_model(fcd = condor_train, 
                                          data_slot = "orig", 
                                          input_type = "expr", 
                                          cluster_slot = "phenograph_expr_orig_k_150", 
                                          cluster_var = "Phenograph",
                                          method = "knn", 
                                          tuneLength = 5, 
                                          trControl = caret::trainControl(method = "cv"), 
                                          seed = 91)
```

```
condor_train_fine$extras$lt_model$performance_plot + ggtitle("Figure S9d_cluster - kNN accuracy")
```

```
# Figure S10 - kNN importance

condor_train_fine$extras$lt_model$features_plot
```

```
condor_test <- predict_labels(fcd = condor_test, 
                              data_slot = "orig", 
                              input_type = "expr", 
                              fcd_model = condor_train_fine, 
                              label = "label_pred_fine", 
                              seed = 91)
```

# Manual annotation of the dataset

```
condor_test <- runPhenograph(fcd = condor_test, 
                             input_type = "expr", 
                             data_slot = "orig", 
                             k = 10, 
                             seed = 91)
```

```
## Run Rphenograph starts:
##   -Input data of 10000 rows and 28 columns
##   -k is set to 10
```

```
##   Finding nearest neighbors...DONE ~ 2.746 s
##   Compute jaccard coefficient between nearest-neighbor sets...
```

```
## Presorting knn...
```

```
## presorting DONE ~ 0.296 s
##   Start jaccard
## DONE ~ 0.003 s
##   Build undirected graph from the weighted links...DONE ~ 0.03 s
##   Run louvain clustering on the graph ...DONE ~ 0.144 s
```

```
## Run Rphenograph DONE, totally takes 2.92299999999989s.
```

```
##   Return a community class
##   -Modularity value: 0.8734183 
##   -Number of clusters: 22
```

## Metaclustering

```
condor_test <- metaclustering(fcd = condor_test, 
                              cluster_slot = "phenograph_expr_orig_k_10", 
                              cluster_var = "Phenograph", 
                              cluster_var_new = "metaclusters", 
                              metaclusters = c("1" = "Classical Monocytes", 
                                               "2" = "CD4 T", 
                                               "3" = "CD8 T", 
                                               "4" = "NK", 
                                               "5" = "CD8 T", 
                                               "6" = "CD8 T", 
                                               "7" = "Classical Monocytes", 
                                               "8" = "Classical Monocytes", 
                                               "9" = "CD4 T", 
                                               "10" = "CD4 T", 
                                               "11" = "Non-classical Monocytes", 
                                               "12" = "CD4 T", 
                                               "13" = "Classical Monocytes",
                                               "14" = "CD8 T",
                                               "15" = "NK",
                                               "16" = "CD8 T",
                                               "17" = "B",
                                               "18" = "CD8 T",
                                               "19" = "Classical Monocytes",
                                               "20" = "NK",
                                               "21" = "pDC",
                                               "22" = "CD8 T"))
```

```
##    cluster             metacluster
## 1        1     Classical Monocytes
## 2        2                   CD4 T
## 3        3                   CD8 T
## 4        4                      NK
## 5        5                   CD8 T
## 6        6                   CD8 T
## 7        7     Classical Monocytes
## 8        8     Classical Monocytes
## 9        9                   CD4 T
## 10      10                   CD4 T
## 11      11 Non-classical Monocytes
## 12      12                   CD4 T
## 13      13     Classical Monocytes
## 14      14                   CD8 T
## 15      15                      NK
## 16      16                   CD8 T
## 17      17                       B
## 18      18                   CD8 T
## 19      19     Classical Monocytes
## 20      20                      NK
## 21      21                     pDC
## 22      22                   CD8 T
```

```
variables <- condor_test$clustering$phenograph_expr_orig_k_10$metaclusters 
group <- condor_test$clustering$label_pred$predicted_label 
size <- 30 
title <- "Figure 6f - confusion matrix"

# quantify cells of each sample per cluster
cells_cluster <- cyCONDOR::confusionMatrix(paste0(variables),
                                   paste0(group))

cells_cluster <- cells_cluster[order(factor(rownames(cells_cluster),levels=c(0:nrow(cells_cluster)))),]

cells_cluster <- cells_cluster[, order(colnames(cells_cluster))]

cells_cluster <- as.matrix(cells_cluster)

# calculate percentage of cells from sample per cluster
scaled_cM <- round((cells_cluster / Matrix::rowSums(cells_cluster))*100,2)

pheatmap::pheatmap(
  mat = t(scaled_cM),
  border_color = "black",display_numbers = TRUE,
  cluster_rows = F,
  cluster_cols = F,
  cellwidth = size,
  cellheight = size,
  main = title)
```

# Plotting for figure 6

## Prepare the dataframe

```
train <- cbind(condor_train$umap$expr_orig, 
               condor_train$clustering$phenograph_expr_orig_k_150[, c(1,3)])

train$type <- "original"

test <- cbind(condor_test$umap$expr_orig, 
              condor_test$clustering$label_pred_fine,
              condor_test$clustering$label_pred)

test$Description <- NULL
test$Description <- NULL

colnames(test) <- c("UMAP1", "UMAP2", "Phenograph", "metaclusters")

test$type <- "predicted"

vis_data <- rbind(train, test)
```

# Overlap UMAP

```
vis_data$type <- factor(vis_data$type, levels = c("original", "predicted"))
```

```
ggplot(data = vis_data, aes(x = UMAP1, y = UMAP2, color = type, alpha = type, size = type)) +
  geom_point_rast() +
  scale_color_manual(values = c("gray", "#92278F")) +
  scale_alpha_manual(values = c(0.5, 1)) +
  scale_size_manual(values = c(0.1, 0.5)) +
  theme_bw() +
  theme(aspect.ratio = 1, panel.grid = element_blank()) + 
  ggtitle("Figure 6c - UMAP projected")
```

```
cluster_palette <- c("#89C5DA", "#DA5724", "#74D944", "#CE50CA", "#3F4921", "#C0717C", "#CBD588", "#5F7FC7",
                     "#673770", "#D3D93E", "#38333E", "#508578", "#D7C1B1", "#689030", "#AD6F3B", "#CD9BCD",
                     "#D14285", "#6DDE88", "#652926", "#7FDCC0", "#C84248", "#8569D5", "#5E738F", "#D1A33D",
                     "#8A7C64", "#599861", "#89C5DA", "#DA5724", "#74D944", "#CE50CA", "#3F4921", "#C0717C", "#CBD588", "#5F7FC7",
                     "#673770", "#D3D93E", "#38333E", "#508578", "#D7C1B1", "#689030", "#AD6F3B", "#CD9BCD",
                     "#D14285", "#6DDE88", "#652926", "#7FDCC0", "#C84248", "#8569D5", "#5E738F", "#D1A33D",
                     "#8A7C64", "#599861")

ggplot(data = vis_data, aes(x = UMAP1, y = UMAP2, color = metaclusters, alpha = type, size = type)) +
  geom_point_rast() +
  scale_color_manual(values = cluster_palette) +
  scale_alpha_manual(values = c(0.01, 1)) +
  scale_size_manual(values = c(0.01, 0.1)) +
  theme_bw() +
  theme(aspect.ratio = 1, panel.grid = element_blank()) + 
  ggtitle("Figure 6e - Predicted cell labels") + facet_wrap(~type)
```

```
ggplot(data = vis_data, aes(x = UMAP1, y = UMAP2, color = Phenograph, alpha = type, size = type)) +
  geom_point_rast() +
  scale_color_manual(values = cluster_palette) +
  scale_alpha_manual(values = c(0.01, 1)) +
  scale_size_manual(values = c(0.01, 0.1)) +
  theme_bw() +
  theme(aspect.ratio = 1, panel.grid = element_blank()) + 
  ggtitle("Figure S11a - Predicted clusters") + facet_wrap(~type)
```

# Calculate LISI score

```
library(lisi)
```

## Prepare dataset

```
set.seed(1960)

train_filt <- train[sample(rownames(train), 10000, replace = FALSE),]

lisi_mat <- rbind(train_filt, test)

res <- compute_lisi(lisi_mat[,c(1,2)], lisi_mat, c('type'))

colnames(res) <- "lisi"

lisi_mat <- cbind(lisi_mat, res)
```

# Visualization

```
ggplot(data = lisi_mat, aes(y = lisi, x = "LISI")) + 
  geom_jitter_rast(alpha = 0.1) +
  geom_violin(alpha = 0.8) +
  theme_bw() +
  theme(aspect.ratio = 2, panel.grid = element_blank()) + ggtitle("Figure 6d - LISI global")
```

```
ggplot(data = lisi_mat, aes(y = lisi, x = metaclusters, fill = metaclusters)) + 
  geom_jitter_rast(alpha = 0.1) +
  geom_violin() +
  theme_bw() +
  scale_fill_manual(values = cluster_palette) +
  theme(aspect.ratio = 1/4, panel.grid = element_blank())+ ggtitle("Figure S11c - LISI metaclusters")
```

```
ggplot(data = lisi_mat, aes(y = lisi, x = Phenograph, fill = Phenograph)) + 
  geom_jitter_rast(alpha = 0.1) +
  geom_violin() +
  theme_bw() +
  scale_fill_manual(values = cluster_palette) +
  theme(aspect.ratio = 1/4, panel.grid = element_blank())+ ggtitle("Figure S11b - LISI clusters")
```

# Session Info

```
info <- sessionInfo()

info
```

```
## R version 4.3.1 (2023-06-16)
## Platform: x86_64-pc-linux-gnu (64-bit)
## Running under: Ubuntu 22.04.3 LTS
## 
## Matrix products: default
## BLAS:   /usr/lib/x86_64-linux-gnu/openblas-pthread/libblas.so.3 
## LAPACK: /usr/lib/x86_64-linux-gnu/openblas-pthread/libopenblasp-r0.3.20.so;  LAPACK version 3.10.0
## 
## locale:
##  [1] LC_CTYPE=en_US.UTF-8       LC_NUMERIC=C              
##  [3] LC_TIME=en_US.UTF-8        LC_COLLATE=en_US.UTF-8    
##  [5] LC_MONETARY=en_US.UTF-8    LC_MESSAGES=en_US.UTF-8   
##  [7] LC_PAPER=en_US.UTF-8       LC_NAME=C                 
##  [9] LC_ADDRESS=C               LC_TELEPHONE=C            
## [11] LC_MEASUREMENT=en_US.UTF-8 LC_IDENTIFICATION=C       
## 
## time zone: Etc/UTC
## tzcode source: system (glibc)
## 
## attached base packages:
## [1] stats     graphics  grDevices utils     datasets  methods   base     
## 
## other attached packages:
## [1] lisi_1.0        caret_6.0-94    lattice_0.22-5  pheatmap_1.0.12
## [5] ggrastr_1.0.2   ggpubr_0.6.0    ggplot2_3.4.4   cyCONDOR_0.2.0 
## 
## loaded via a namespace (and not attached):
##   [1] IRanges_2.34.1              Rmisc_1.5.1                
##   [3] urlchecker_1.0.1            nnet_7.3-19                
##   [5] CytoNorm_2.0.1              TH.data_1.1-2              
##   [7] vctrs_0.6.4                 digest_0.6.33              
##   [9] png_0.1-8                   shape_1.4.6                
##  [11] proxy_0.4-27                slingshot_2.8.0            
##  [13] ggrepel_0.9.4               parallelly_1.36.0          
##  [15] MASS_7.3-60                 reshape2_1.4.4             
##  [17] httpuv_1.6.12               foreach_1.5.2              
##  [19] BiocGenerics_0.46.0         withr_2.5.1                
##  [21] xfun_0.40                   ellipsis_0.3.2             
##  [23] survival_3.5-7              memoise_2.0.1              
##  [25] hexbin_1.28.3               ggbeeswarm_0.7.2           
##  [27] RProtoBufLib_2.12.1         princurve_2.1.6            
##  [29] profvis_0.3.8               ggsci_3.0.0                
##  [31] zoo_1.8-12                  GlobalOptions_0.1.2        
##  [33] DEoptimR_1.1-3              Formula_1.2-5              
##  [35] prettyunits_1.2.0           promises_1.2.1             
##  [37] scatterplot3d_0.3-44        rstatix_0.7.2              
##  [39] globals_0.16.2              ps_1.7.5                   
##  [41] rstudioapi_0.15.0           miniUI_0.1.1.1             
##  [43] generics_0.1.3              ggcyto_1.28.1              
##  [45] base64enc_0.1-3             processx_3.8.2             
##  [47] curl_5.1.0                  S4Vectors_0.38.2           
##  [49] zlibbioc_1.46.0             flowWorkspace_4.12.2       
##  [51] polyclip_1.10-6             randomForest_4.7-1.1       
##  [53] GenomeInfoDbData_1.2.10     RBGL_1.76.0                
##  [55] ncdfFlow_2.46.0             RcppEigen_0.3.3.9.4        
##  [57] xtable_1.8-4                stringr_1.5.0              
##  [59] doParallel_1.0.17           evaluate_0.22              
##  [61] S4Arrays_1.0.6              hms_1.1.3                  
##  [63] glmnet_4.1-8                GenomicRanges_1.52.1       
##  [65] irlba_2.3.5.1               colorspace_2.1-0           
##  [67] harmony_1.1.0               reticulate_1.34.0          
##  [69] readxl_1.4.3                magrittr_2.0.3             
##  [71] lmtest_0.9-40               readr_2.1.4                
##  [73] Rgraphviz_2.44.0            later_1.3.1                
##  [75] future.apply_1.11.0         robustbase_0.99-0          
##  [77] XML_3.99-0.15               cowplot_1.1.1              
##  [79] matrixStats_1.1.0           RcppAnnoy_0.0.21           
##  [81] xts_0.13.1                  class_7.3-22               
##  [83] Hmisc_5.1-1                 pillar_1.9.0               
##  [85] nlme_3.1-163                iterators_1.0.14           
##  [87] compiler_4.3.1              RSpectra_0.16-1            
##  [89] stringi_1.7.12              gower_1.0.1                
##  [91] minqa_1.2.6                 SummarizedExperiment_1.30.2
##  [93] lubridate_1.9.3             devtools_2.4.5             
##  [95] CytoML_2.12.0               plyr_1.8.9                 
##  [97] crayon_1.5.2                abind_1.4-5                
##  [99] locfit_1.5-9.8              sp_2.1-1                   
## [101] sandwich_3.0-2              pcaMethods_1.92.0          
## [103] dplyr_1.1.3                 codetools_0.2-19           
## [105] multcomp_1.4-25             recipes_1.0.8              
## [107] openssl_2.1.1               Rphenograph_0.99.1         
## [109] TTR_0.24.3                  bslib_0.5.1                
## [111] e1071_1.7-13                destiny_3.14.0             
## [113] GetoptLong_1.0.5            ggplot.multistats_1.0.0    
## [115] mime_0.12                   splines_4.3.1              
## [117] circlize_0.4.15             Rcpp_1.0.11                
## [119] sparseMatrixStats_1.12.2    cellranger_1.1.0           
## [121] knitr_1.44                  utf8_1.2.4                 
## [123] clue_0.3-65                 lme4_1.1-35.1              
## [125] fs_1.6.3                    listenv_0.9.0              
## [127] checkmate_2.3.0             DelayedMatrixStats_1.22.6  
## [129] pkgbuild_1.4.2              ggsignif_0.6.4             
## [131] tibble_3.2.1                Matrix_1.6-1.1             
## [133] rpart.plot_3.1.1            callr_3.7.3                
## [135] tzdb_0.4.0                  tweenr_2.0.2               
## [137] pkgconfig_2.0.3             tools_4.3.1                
## [139] cachem_1.0.8                smoother_1.1               
## [141] fastmap_1.1.1               rmarkdown_2.25             
## [143] scales_1.2.1                grid_4.3.1                 
## [145] usethis_2.2.2               broom_1.0.5                
## [147] sass_0.4.7                  graph_1.78.0               
## [149] carData_3.0-5               RANN_2.6.1                 
## [151] rpart_4.1.21                farver_2.1.1               
## [153] yaml_2.3.7                  MatrixGenerics_1.12.3      
## [155] foreign_0.8-85              ggthemes_4.2.4             
## [157] cli_3.6.1                   purrr_1.0.2                
## [159] stats4_4.3.1                lifecycle_1.0.3            
## [161] uwot_0.1.16                 askpass_1.2.0              
## [163] Biobase_2.60.0              mvtnorm_1.2-3              
## [165] lava_1.7.3                  sessioninfo_1.2.2          
## [167] backports_1.4.1             cytolib_2.12.1             
## [169] timechange_0.2.0            gtable_0.3.4               
## [171] rjson_0.2.21                umap_0.2.10.0              
## [173] ggridges_0.5.4              Rphenoannoy_0.1.0          
## [175] parallel_4.3.1              pROC_1.18.5                
## [177] limma_3.56.2                jsonlite_1.8.7             
## [179] edgeR_3.42.4                RcppHNSW_0.5.0             
## [181] bitops_1.0-7                Rtsne_0.16                 
## [183] FlowSOM_2.8.0               ranger_0.16.0              
## [185] flowCore_2.12.2             jquerylib_0.1.4            
## [187] timeDate_4022.108           shiny_1.7.5.1              
## [189] ConsensusClusterPlus_1.64.0 htmltools_0.5.6.1          
## [191] diffcyt_1.20.0              glue_1.6.2                 
## [193] XVector_0.40.0              VIM_6.2.2                  
## [195] RCurl_1.98-1.13             gridExtra_2.3              
## [197] boot_1.3-28.1               igraph_1.5.1               
## [199] TrajectoryUtils_1.8.0       R6_2.5.1                   
## [201] tidyr_1.3.0                 SingleCellExperiment_1.22.0
## [203] labeling_0.4.3              vcd_1.4-11                 
## [205] cluster_2.1.4               pkgload_1.3.3              
## [207] GenomeInfoDb_1.36.4         ipred_0.9-14               
## [209] nloptr_2.0.3                DelayedArray_0.26.7        
## [211] tidyselect_1.2.0            vipor_0.4.5                
## [213] htmlTable_2.4.2             ggforce_0.4.1              
## [215] CytoDx_1.20.0               car_3.1-2                  
## [217] future_1.33.0               ModelMetrics_1.2.2.2       
## [219] munsell_0.5.0               laeken_0.5.2               
## [221] data.table_1.14.8           htmlwidgets_1.6.2          
## [223] ComplexHeatmap_2.16.0       RColorBrewer_1.1-3         
## [225] rlang_1.1.1                 remotes_2.4.2.1            
## [227] colorRamps_2.3.1            Cairo_1.6-1                
## [229] ggnewscale_0.4.9            fansi_1.0.5                
## [231] hardhat_1.3.0               beeswarm_0.4.0             
## [233] prodlim_2023.08.28
```
