# Supplementary material for: Unveiling the power of high-dimensional cytometry data with cyCONDOR
Source: Nat Commun. 2024 Dec 19;15:10702. doi: 10.1038/s41467-024-55179-w (PMC11659560; doi:10.1038/s41467-024-55179-w)
Supplement: Supplementary file 21 — Supplementary Data 19 [file 41467_2024_55179_MOESM21_ESM.html]

Supplementary Data 19: reproducibility data for Figure 7 - CytoDX example


# Supplementary Data 19: reproducibility data for Figure 7 - CytoDX example

```
library(cyCONDOR)
library(ggplot2)
library(ggpubr)
```

# Loading the data - Training Set

```
condor <- prep_fcd(data_path = "/home/user/data/Data/Figure 7 - Clinical Classifier/data_and_envs/CytoDX/train/", 
                    max_cell = 10000000, 
                    useCSV = FALSE, 
                    transformation = "auto_logi", 
                    remove_param = c("FSC-A","FSC-W","FSC-H","Time"), 
                    anno_table = "/home/user/data/Data/Figure 7 - Clinical Classifier/data_and_envs/CytoDX/fcs_info_train.csv", 
                    filename_col = "fcsName",
                    seed = 91, 
                    verbose = TRUE)
```

```
## [1] "Start reading the data"
## [1] "Loading file 1 out of 10"
## [1] "Loading file 2 out of 10"
## [1] "Loading file 3 out of 10"
## [1] "Loading file 4 out of 10"
## [1] "Loading file 5 out of 10"
## [1] "Loading file 6 out of 10"
## [1] "Loading file 7 out of 10"
## [1] "Loading file 8 out of 10"
## [1] "Loading file 9 out of 10"
## [1] "Loading file 10 out of 10"
## [1] "Start transforming the data"
## [1] "FS Lin w= 0 t= 1023"
## [1] "SS Log w= 0 t= 1420.18115234375"
## [1] "CD15-FITC w= 0 t= 3651.7412109375"
## [1] "CD13-PE w= 0 t= 3651.7412109375"
## [1] "CD45-ECD w= 0 t= 2665.515625"
## [1] "CD16-PC5 w= 0 t= 9139.8173828125"
## [1] "CD56-PC7 w= 0 t= 2167.39208984375"
```

```
class(condor)
```

```
## [1] "flow_cytometry_dataframe"
```

## Visualize the data on a UMAP

```
condor <- runPCA(fcd = condor, 
                 data_slot = "orig", 
                 seed = 91)
```

```
condor <- runUMAP(fcd = condor, 
                  input_type = "pca", 
                  data_slot = "orig", 
                  seed = 91)
```

```
plot_dim_red(fcd = condor, 
             expr_slot = "orig", 
             reduction_method = "umap", 
             reduction_slot = "pca_orig", 
             cluster_slot = NULL,
             param = "Label", 
             order = T, 
             title = "Figure 7b - UMAP training data", 
             facet_by_variable = FALSE, 
             color_discrete = c("#92278F", "#F15A29"), 
             raster = TRUE, 
             alpha = 1, 
             dot_size = 1)
```

## Build the classifier model

```
# Re order variables

condor$anno$cell_anno$Label <- factor(condor$anno$cell_anno$Label, levels = c("normal", "aml"), labels = c("1_normal", "2_aml"))
```

```
condor <- train_classifier_model(fcd = condor, 
                                 input_type = "expr", 
                                 data_slot = "orig", 
                                 sample_names = "expfcs_filename", 
                                 classification_variable = condor$anno$cell_anno$Label, 
                                 family = "binomial", 
                                 type1 = "response", 
                                 parallelCore = 1, 
                                 reg = FALSE, 
                                 seed = 91)
```

```
## Warning in lognet(xd, is.sparse, ix, jx, y, weights, offset, alpha, nobs, : one
## multinomial or binomial class has fewer than 8 observations; dangerous ground
```

### Explore the model

```
condor$extras$classifier_model$train.Data.sample
```

```
##                    sample y1.Truth    y.Pred.s0
## sample11.fcs sample11.fcs    2_aml 1.000000e+00
## sample12.fcs sample12.fcs    2_aml 1.000000e+00
## sample13.fcs sample13.fcs    2_aml 9.999999e-01
## sample14.fcs sample14.fcs    2_aml 1.000000e+00
## sample15.fcs sample15.fcs    2_aml 1.000000e+00
## sample16.fcs sample16.fcs 1_normal 5.949578e-14
## sample17.fcs sample17.fcs 1_normal 6.325788e-17
## sample18.fcs sample18.fcs 1_normal 2.428552e-13
## sample19.fcs sample19.fcs 1_normal 6.003047e-08
## sample20.fcs sample20.fcs 1_normal 1.747054e-13
```

```
condor$extras$classifier_model$train.Data.cell[1:10,]
```

```
##          sample y1.Truth y.Pred.s0
## 1  sample11.fcs    2_aml 0.5836773
## 2  sample11.fcs    2_aml 0.5299637
## 3  sample11.fcs    2_aml 0.6896542
## 4  sample11.fcs    2_aml 0.4914881
## 5  sample11.fcs    2_aml 0.5393115
## 6  sample11.fcs    2_aml 0.3407959
## 7  sample11.fcs    2_aml 0.6714140
## 8  sample11.fcs    2_aml 0.6862076
## 9  sample11.fcs    2_aml 0.8487911
## 10 sample11.fcs    2_aml 0.8658376
```

# Load the data - test Set

```
condor_test <- prep_fcd(data_path = "/home/user/data/Data/Figure 7 - Clinical Classifier/data_and_envs/CytoDX/test/", 
                      max_cell = 10000000, 
                    useCSV = FALSE, 
                    transformation = "auto_logi", 
                    remove_param = c("FSC-A","FSC-W","FSC-H","Time"), 
                    anno_table = "/home/user/data/Data/Figure 7 - Clinical Classifier/data_and_envs/CytoDX/fcs_info_test.csv", 
                    filename_col = "fcsName",
                    seed = 91)
```

```
class(condor_test)
```

```
## [1] "flow_cytometry_dataframe"
```

## Predict classification

```
# Re order variables

condor_test$anno$cell_anno$Label <- factor(condor_test$anno$cell_anno$Label, levels = c("normal", "aml"), labels = c("1_normal", "2_aml"))
```

```
condor_test <- predict_classifier(fcd = condor_test, 
                                  input_type = "expr", 
                                  data_slot = "orig", 
                                  sample_names = "expfcs_filename", 
                                  model_object = condor$extras$classifier_model, 
                                  seed = 91)
```

### Explore Prediction

```
condor_test$extras$classifier_prediction$xNew.Pred.sample
```

```
##                    sample    y.Pred.s0
## sample1.fcs   sample1.fcs 1.000000e+00
## sample10.fcs sample10.fcs 6.021657e-12
## sample2.fcs   sample2.fcs 1.000000e+00
## sample3.fcs   sample3.fcs 9.998114e-01
## sample4.fcs   sample4.fcs 1.000000e+00
## sample5.fcs   sample5.fcs 1.000000e+00
## sample6.fcs   sample6.fcs 1.944953e-15
## sample7.fcs   sample7.fcs 5.864772e-03
## sample8.fcs   sample8.fcs 1.735012e-03
## sample9.fcs   sample9.fcs 3.344107e-17
```

```
condor_test$extras$classifier_prediction$xNew.Pred.cell[1:10,]
```

```
##         sample y.Pred.s0
## 1  sample1.fcs 0.6212374
## 2  sample1.fcs 0.6780328
## 3  sample1.fcs 0.5818562
## 4  sample1.fcs 0.3354043
## 5  sample1.fcs 0.4015879
## 6  sample1.fcs 0.7143018
## 7  sample1.fcs 0.5221815
## 8  sample1.fcs 0.4555662
## 9  sample1.fcs 0.7290862
## 10 sample1.fcs 0.5975344
```

# Visualize the results

## Training Data

```
anno <- read.csv("/home/user/data/Data/Figure 7 - Clinical Classifier/data_and_envs/CytoDX/fcs_info_train.csv")

tmp <- merge(x = condor$extras$classifier_model$train.Data.cell, y = anno, by.x = "sample", by.y = "fcsName")

ggplot(tmp, aes(x = sample, y = y.Pred.s0, color = Label)) +
  geom_jitter() + 
  geom_violin() +
  scale_color_manual(values = c("#92278F", "#F15A29")) +
  theme_bw() + 
  theme(aspect.ratio = 1) + 
  ggtitle("Figure S12b - Training prediction cell")
```

```
tmp <- merge(x = condor$extras$classifier_model$train.Data.sample, y = anno, by.x = "sample", by.y = "fcsName")

ggplot(tmp, aes(x = sample, y = y.Pred.s0, color = Label)) +
  geom_point(size = 4) +
  scale_color_manual(values = c("#92278F", "#F15A29")) +
  theme_bw() + 
  theme(aspect.ratio = 2) + 
  ggtitle("Figure S12c - Training prediction sample")
```

## Test Data

```
anno <- read.csv("/home/user/data/Data/Figure 7 - Clinical Classifier/data_and_envs/CytoDX/fcs_info_test.csv")

tmp <- merge(x = condor_test$extras$classifier_prediction$xNew.Pred.cell, y = anno, by.x = "sample", by.y = "fcsName")

tmp$sample <- factor(tmp$sample, levels = c("sample1.fcs", "sample2.fcs", "sample3.fcs",  "sample4.fcs",  "sample5.fcs",  "sample6.fcs",  "sample7.fcs",  "sample8.fcs",  "sample9.fcs", "sample10.fcs"))

ggplot(tmp, aes(x = sample, y = y.Pred.s0, color = Label)) +
  geom_jitter() +
  geom_violin() +
  scale_color_manual(values = c("#92278F", "#F15A29")) +
  theme_bw() + 
  theme(aspect.ratio = 1) + 
  ggtitle("Figure 7c - Test prediction cell")
```

```
tmp <- merge(x = condor_test$extras$classifier_prediction$xNew.Pred.sample, y = anno, by.x = "sample", by.y = "fcsName")

tmp$sample <- factor(tmp$sample, levels = c("sample1.fcs", "sample2.fcs", "sample3.fcs",  "sample4.fcs",  "sample5.fcs",  "sample6.fcs",  "sample7.fcs",  "sample8.fcs",  "sample9.fcs", "sample10.fcs"))

ggplot(tmp, aes(x = sample, y = y.Pred.s0, color = Label)) +
  geom_point(size = 4) +
  scale_color_manual(values = c("#92278F", "#F15A29")) +
  theme_bw() + 
  theme(aspect.ratio = 2) + 
  ggtitle("Figure 7d - Test prediction sample")
```

# Visualization of the decision three

```
# Figure S12d - Decision tree

library(CytoDx)

treeGate(P = condor$extras$classifier_model$train.Data.cell$y.Pred.s0,
         x= condor$expr$orig)
```

```
## n= 5000 
## 
## node), split, n, deviance, yval
##       * denotes terminal node
## 
##  1) root 5000 219.240700 0.5000000  
##    2) SS_Log>=3.419922 1900  64.191190 0.4192114  
##      4) CD16_PC5>=3.490234 430  10.491470 0.3107815 *
##      5) CD16_PC5< 3.490234 1470  47.165360 0.4509290  
##       10) CD45_ECD< 2.822267 349  12.859740 0.3274761 *
##       11) CD45_ECD>=2.822267 1121  27.330690 0.4893635 *
##    3) SS_Log< 3.419922 3100 135.048000 0.5495156  
##      6) CD45_ECD< 2.089872 156  10.780670 0.4014948  
##       12) CD16_PC5< 0.9622334 108   4.707132 0.3080286 *
##       13) CD16_PC5>=0.9622334 48   3.007226 0.6117938 *
##      7) CD45_ECD>=2.089872 2944 120.668300 0.5573591  
##       14) CD16_PC5>=1.424444 354  19.995640 0.4590620  
##         28) CD13_PE>=3.117188 98   2.950622 0.2241499 *
##         29) CD13_PE< 3.117188 256   9.566762 0.5489893  
##           58) CD15_FITC>=1.572577 180   4.024033 0.4759978 *
##           59) CD15_FITC< 1.572577 76   2.312419 0.7218641 *
##       15) CD16_PC5< 1.424444 2590  96.784670 0.5707943  
##         30) CD13_PE< 1.849696 1211  39.681310 0.5306148  
##           60) CD16_PC5< 0.6143322 663  20.381500 0.4808327 *
##           61) CD16_PC5>=0.6143322 548  15.668830 0.5908438 *
##         31) CD13_PE>=1.849696 1379  53.431480 0.6060788  
##           62) CD45_ECD>=3.644531 94   6.251842 0.4383146 *
##           63) CD45_ECD< 3.644531 1285  44.340490 0.6183511 *
```

# Session Info

```
info <- sessionInfo()

info
```

```
## R version 4.3.1 (2023-06-16)
## Platform: x86_64-pc-linux-gnu (64-bit)
## Running under: Ubuntu 22.04.3 LTS
## 
## Matrix products: default
## BLAS:   /usr/lib/x86_64-linux-gnu/openblas-pthread/libblas.so.3 
## LAPACK: /usr/lib/x86_64-linux-gnu/openblas-pthread/libopenblasp-r0.3.20.so;  LAPACK version 3.10.0
## 
## locale:
##  [1] LC_CTYPE=en_US.UTF-8       LC_NUMERIC=C              
##  [3] LC_TIME=en_US.UTF-8        LC_COLLATE=en_US.UTF-8    
##  [5] LC_MONETARY=en_US.UTF-8    LC_MESSAGES=en_US.UTF-8   
##  [7] LC_PAPER=en_US.UTF-8       LC_NAME=C                 
##  [9] LC_ADDRESS=C               LC_TELEPHONE=C            
## [11] LC_MEASUREMENT=en_US.UTF-8 LC_IDENTIFICATION=C       
## 
## time zone: Etc/UTC
## tzcode source: system (glibc)
## 
## attached base packages:
## [1] stats     graphics  grDevices utils     datasets  methods   base     
## 
## other attached packages:
## [1] CytoDx_1.20.0  ggpubr_0.6.0   ggplot2_3.4.4  cyCONDOR_0.2.0
## 
## loaded via a namespace (and not attached):
##   [1] IRanges_2.34.1              Rmisc_1.5.1                
##   [3] urlchecker_1.0.1            nnet_7.3-19                
##   [5] CytoNorm_2.0.1              TH.data_1.1-2              
##   [7] vctrs_0.6.4                 digest_0.6.33              
##   [9] png_0.1-8                   shape_1.4.6                
##  [11] proxy_0.4-27                slingshot_2.8.0            
##  [13] ggrepel_0.9.4               parallelly_1.36.0          
##  [15] MASS_7.3-60                 reshape2_1.4.4             
##  [17] httpuv_1.6.12               foreach_1.5.2              
##  [19] BiocGenerics_0.46.0         withr_2.5.1                
##  [21] ggrastr_1.0.2               xfun_0.40                  
##  [23] ellipsis_0.3.2              survival_3.5-7             
##  [25] memoise_2.0.1               hexbin_1.28.3              
##  [27] ggbeeswarm_0.7.2            RProtoBufLib_2.12.1        
##  [29] princurve_2.1.6             profvis_0.3.8              
##  [31] ggsci_3.0.0                 zoo_1.8-12                 
##  [33] GlobalOptions_0.1.2         DEoptimR_1.1-3             
##  [35] Formula_1.2-5               prettyunits_1.2.0          
##  [37] promises_1.2.1              scatterplot3d_0.3-44       
##  [39] rstatix_0.7.2               globals_0.16.2             
##  [41] ps_1.7.5                    rstudioapi_0.15.0          
##  [43] miniUI_0.1.1.1              generics_0.1.3             
##  [45] ggcyto_1.28.1               base64enc_0.1-3            
##  [47] processx_3.8.2              curl_5.1.0                 
##  [49] S4Vectors_0.38.2            zlibbioc_1.46.0            
##  [51] flowWorkspace_4.12.2        polyclip_1.10-6            
##  [53] randomForest_4.7-1.1        GenomeInfoDbData_1.2.10    
##  [55] RBGL_1.76.0                 ncdfFlow_2.46.0            
##  [57] RcppEigen_0.3.3.9.4         xtable_1.8-4               
##  [59] stringr_1.5.0               doParallel_1.0.17          
##  [61] evaluate_0.22               S4Arrays_1.0.6             
##  [63] hms_1.1.3                   glmnet_4.1-8               
##  [65] GenomicRanges_1.52.1        irlba_2.3.5.1              
##  [67] colorspace_2.1-0            harmony_1.1.0              
##  [69] reticulate_1.34.0           readxl_1.4.3               
##  [71] magrittr_2.0.3              lmtest_0.9-40              
##  [73] readr_2.1.4                 Rgraphviz_2.44.0           
##  [75] later_1.3.1                 lattice_0.22-5             
##  [77] future.apply_1.11.0         robustbase_0.99-0          
##  [79] XML_3.99-0.15               cowplot_1.1.1              
##  [81] matrixStats_1.1.0           RcppAnnoy_0.0.21           
##  [83] xts_0.13.1                  class_7.3-22               
##  [85] Hmisc_5.1-1                 pillar_1.9.0               
##  [87] nlme_3.1-163                iterators_1.0.14           
##  [89] compiler_4.3.1              RSpectra_0.16-1            
##  [91] stringi_1.7.12              gower_1.0.1                
##  [93] minqa_1.2.6                 SummarizedExperiment_1.30.2
##  [95] lubridate_1.9.3             devtools_2.4.5             
##  [97] CytoML_2.12.0               plyr_1.8.9                 
##  [99] crayon_1.5.2                abind_1.4-5                
## [101] locfit_1.5-9.8              sp_2.1-1                   
## [103] sandwich_3.0-2              pcaMethods_1.92.0          
## [105] dplyr_1.1.3                 codetools_0.2-19           
## [107] multcomp_1.4-25             recipes_1.0.8              
## [109] openssl_2.1.1               Rphenograph_0.99.1         
## [111] TTR_0.24.3                  bslib_0.5.1                
## [113] e1071_1.7-13                destiny_3.14.0             
## [115] GetoptLong_1.0.5            ggplot.multistats_1.0.0    
## [117] mime_0.12                   splines_4.3.1              
## [119] circlize_0.4.15             Rcpp_1.0.11                
## [121] sparseMatrixStats_1.12.2    cellranger_1.1.0           
## [123] knitr_1.44                  utf8_1.2.4                 
## [125] clue_0.3-65                 lme4_1.1-35.1              
## [127] fs_1.6.3                    listenv_0.9.0              
## [129] checkmate_2.3.0             DelayedMatrixStats_1.22.6  
## [131] pkgbuild_1.4.2              ggsignif_0.6.4             
## [133] tibble_3.2.1                Matrix_1.6-1.1             
## [135] rpart.plot_3.1.1            callr_3.7.3                
## [137] tzdb_0.4.0                  tweenr_2.0.2               
## [139] pkgconfig_2.0.3             pheatmap_1.0.12            
## [141] tools_4.3.1                 cachem_1.0.8               
## [143] smoother_1.1                fastmap_1.1.1              
## [145] rmarkdown_2.25              scales_1.2.1               
## [147] grid_4.3.1                  usethis_2.2.2              
## [149] broom_1.0.5                 sass_0.4.7                 
## [151] graph_1.78.0                carData_3.0-5              
## [153] RANN_2.6.1                  rpart_4.1.21               
## [155] farver_2.1.1                yaml_2.3.7                 
## [157] MatrixGenerics_1.12.3       foreign_0.8-85             
## [159] ggthemes_4.2.4              cli_3.6.1                  
## [161] purrr_1.0.2                 stats4_4.3.1               
## [163] lifecycle_1.0.3             uwot_0.1.16                
## [165] askpass_1.2.0               caret_6.0-94               
## [167] Biobase_2.60.0              mvtnorm_1.2-3              
## [169] lava_1.7.3                  sessioninfo_1.2.2          
## [171] backports_1.4.1             cytolib_2.12.1             
## [173] timechange_0.2.0            gtable_0.3.4               
## [175] rjson_0.2.21                umap_0.2.10.0              
## [177] ggridges_0.5.4              parallel_4.3.1             
## [179] pROC_1.18.5                 limma_3.56.2               
## [181] jsonlite_1.8.7              edgeR_3.42.4               
## [183] RcppHNSW_0.5.0              bitops_1.0-7               
## [185] Rtsne_0.16                  FlowSOM_2.8.0              
## [187] ranger_0.16.0               flowCore_2.12.2            
## [189] jquerylib_0.1.4             timeDate_4022.108          
## [191] shiny_1.7.5.1               ConsensusClusterPlus_1.64.0
## [193] htmltools_0.5.6.1           diffcyt_1.20.0             
## [195] glue_1.6.2                  XVector_0.40.0             
## [197] VIM_6.2.2                   RCurl_1.98-1.13            
## [199] gridExtra_2.3               boot_1.3-28.1              
## [201] igraph_1.5.1                TrajectoryUtils_1.8.0      
## [203] R6_2.5.1                    tidyr_1.3.0                
## [205] SingleCellExperiment_1.22.0 labeling_0.4.3             
## [207] vcd_1.4-11                  cluster_2.1.4              
## [209] pkgload_1.3.3               GenomeInfoDb_1.36.4        
## [211] ipred_0.9-14                nloptr_2.0.3               
## [213] DelayedArray_0.26.7         tidyselect_1.2.0           
## [215] vipor_0.4.5                 htmlTable_2.4.2            
## [217] ggforce_0.4.1               car_3.1-2                  
## [219] future_1.33.0               ModelMetrics_1.2.2.2       
## [221] munsell_0.5.0               laeken_0.5.2               
## [223] data.table_1.14.8           htmlwidgets_1.6.2          
## [225] ComplexHeatmap_2.16.0       RColorBrewer_1.1-3         
## [227] rlang_1.1.1                 remotes_2.4.2.1            
## [229] colorRamps_2.3.1            Cairo_1.6-1                
## [231] ggnewscale_0.4.9            fansi_1.0.5                
## [233] hardhat_1.3.0               beeswarm_0.4.0             
## [235] prodlim_2023.08.28
```
