# Supplementary material for: Unveiling the power of high-dimensional cytometry data with cyCONDOR
Source: Nat Commun. 2024 Dec 19;15:10702. doi: 10.1038/s41467-024-55179-w (PMC11659560; doi:10.1038/s41467-024-55179-w)
Supplement: Supplementary file 22 — Supplementary Data 20 [file 41467_2024_55179_MOESM22_ESM.html]

Supplementary Data 20: reproducibility data for Figure 7 - FlowCapII perfomances


# Supplementary Data 20: reproducibility data for Figure 7 - FlowCapII perfomances

The permutation was calculated with cyCONDOR v0.1.5. Nevertheless the
code was updated to work with cyCONDOR v0.2.0 and the results of the
first few permutation was evaluated for consistency with the old
version. The metrics show here are explain in details in this nice wiki
page https://en.wikipedia.org/wiki/Precision\_and\_recall.

```
library(cyCONDOR, quietly = TRUE)
```

# Loading the metadata from the FlowCAP II dataset

```
# FCII_meta <- read.csv("../Datasets/FC_FlowCapII_FR-FCM-ZZYA/.attachments/AML.csv")
# 
# FCII_meta$FCS.file <- stringr::str_replace(string = FCII_meta$FCS.file, pattern = ".FCS", replacement = ".fcs")
```

# Running the loop for the predicion performances

## Preparing the collectors

```
# # First collector for the prediction values
# collector <- list()
# 
# # Second collector for the performance values
# table_collector <- matrix(ncol = 9, nrow = 0)
# 
# colnames(table_collector) <- c("P", "TP", "FP", "N", "TN", "FN", "SENS", "SPEC", "ACC")
```

## Running the loop

```
# # Now running the loops with a seed going from 1 to 100
# for (i in seq(1,100)) {
# 
#   print(i)
# 
#   # Set the seed
#   set.seed(i)
# 
#   # Select the sample for the train dataset (80%)
#   train_sample <- sample(unique(FCII_meta$Individual),
#                          size = round(length(unique(FCII_meta$Individual))*0.8),
#                          replace = FALSE)
#   # Select sample for the test dataset (remaining 20%)
#   test_sample <- unique(FCII_meta$Individual)[!unique(FCII_meta$Individual) %in% train_sample]
# 
#   # Exctract sample name and save the annotation file
#   FCII_list <- list()
# 
#   for (pannel in unique(FCII_meta$Tube.number)) {
# 
#     df <- FCII_meta[FCII_meta$Tube.number == pannel,]
# 
#     df_train <- df[df$Individual %in% train_sample,]
# 
#     # Check number of aml in train data
#     n_aml <- sum(df_train$Condition == "aml")
#     n_normal <- sum(df_train$Condition == "normal")
# 
#     # Balance the train dataset
#     diff <- n_normal - n_aml
# 
#     df_train <- df_train[!df_train$FCS.file %in% sample(df_train[df_train$Condition == "normal",]$FCS.file, size = diff, replace = FALSE), ]
# 
#     df_test <- df[df$Individual %in% test_sample,]
# 
#     FCII_list[[paste("pannel_", pannel, sep = "")]][["train"]] <- df_train
# 
#     FCII_list[[paste("pannel_", pannel, sep = "")]][["test"]] <- df_test
# 
#   }
# 
#   for (pannel in names(FCII_list)) {
# 
#     tmp <- FCII_list[[pannel]]
# 
#     for (df in names(tmp)) {
# 
#       write.csv(file = paste("./data_and_envs/FlowCapII/", pannel, "_", df, ".csv", sep = ""), x = tmp[[df]], row.names = FALSE)
# 
#     }
# 
#   }
# 
#   # Prepare CONDOR object on the train dataset
#   condor <- prep_fcd(data_path = "./data_and_envs/Pannel_4/",
#                      max_cell = 3000,
#                      useCSV = FALSE,
#                      transformation = "auto_logi",
#                      remove_param = c("Time", "InFile"),
#                      anno_table = "./data_and_envs/FlowCapII/pannel_4_train.csv",
#                      filename_col = "FCS.file",
#                      seed = 91)
# 
#   # Re order the variable to have the prediction for AML
#   condor$anno$cell_anno$Condition <- factor(condor$anno$cell_anno$Condition,
#                                             levels = c("normal", "aml"), labels = c("1_normal", "2_aml"))
# 
# 
#   # Train classifier on the training data
#   condor <- train_classifier_model(fcd = condor,
#                                    input_type = "expr",
#                                    data_slot = "orig",
#                                    sample_names = "expfcs_filename",
#                                    classification_variable = condor$anno$cell_anno$Condition,
#                                    family = "binomial",
#                                    type1 = "response",
#                                    parallelCore = 1,
#                                    reg = FALSE,
#                                    seed = 91)
# 
#   # Load test data in a condor object
#   condor_test <- prep_fcd(data_path = "./data_and_envs/Pannel_4/",
#                           max_cell = 3000,
#                           useCSV = FALSE,
#                           transformation = "auto_logi",
#                           remove_param = c("Time", "InFile"),
#                           anno_table = "./data_and_envs/FlowCapII/pannel_4_test.csv",
#                           filename_col = "FCS.file",
#                           seed = 91)
# 
#   condor_test$anno$cell_anno$Condition <- factor(condor_test$anno$cell_anno$Condition,
#                                                  levels = c("normal", "aml"), labels = c("1_normal", "2_aml"))
# 
#   # Predict the label of the test dataset
#   condor_test <- predict_classifier(fcd = condor_test,
#                                     input_type = "expr",
#                                     data_slot = "orig",
#                                     sample_names = "expfcs_filename",
#                                     model_object = condor$extras$classifier_model,
#                                     seed = 91)
# 
#   # Merge result with original annotation
#   anno <- read.csv("./data_and_envs/FlowCapII/pannel_4_test.csv")
# 
#   tmp <- merge(x = condor_test$extras$classifier_prediction$xNew.Pred.sample, y = anno, by.x = "sample", by.y = "FCS.file")
# 
#   # Include a column with the predicted labels (according to a probability of 50%)
#   tmp$Pred <- ifelse(tmp$y.Pred.s0 >= 0.5, "pred_aml", "pred_normal")
# 
#   tmp <- tmp[, c("sample", "Condition", "Pred", "y.Pred.s0")]
# 
#   # Store the results in a list
#   collector[[paste0("Pred_seed_", i)]] <- tmp
# 
#   table <- table(tmp$Condition, tmp$Pred)
# 
#   # Calculate performance parameters
#   P <- sum(table[1,1] + table[1,2]) # Total positive samples
#   TP <- table[1,1] # True positive prediction
#   FP <- table[2,1] # False positive prediction
# 
#   N <- sum(table[2,1] + table[2,2]) # Total negative samples
#   TN <- table[2,2] # True negative prediction
#   FN <- table[1,2] # False negative prediction
# 
#   SENS <- TP/P # Sensitivity
#   SPEC <- ifelse(N > 0, TN/N, 0) # Specificity
#   ACC <- (TP+TN)/(TP+FP+FN+TN) # Accuracy
# 
#   # Store the perfromacne data in a table
#   table_collector <- rbind(table_collector, c(P, TP, FP, N, TN, FN, SENS, SPEC, ACC))
# 
#   # Clean
#   rm(anno, condor, condor_test, df_test, df_train, FCII_list, tmp, ACC, df, FN, FP, i, N, P, pannel,
#      SENS, SPEC, table, test_sample, TN, TP, train_sample)
# 
#   # Do a garbage collection to avoid overload of systems memory
#   gc()
# 
# }
```

## Save output

```
# saveRDS(collector, file = "./data_and_envs/loop_output/loop_output_probability_balanced.R")
# 
# saveRDS(table_collector, file = "./data_and_envs/loop_output/loop_stats_balanced.R")
```

## Load output

```
collector <- readRDS("./data_and_envs/loop_output/loop_output_probability_balanced.R")

table_collector <- readRDS("./data_and_envs/loop_output/loop_stats_balanced.R")
```

## Stats visualization

```
library(reshape2)

df <- melt(as.data.frame(table_collector[, c(7,8,9)]))
```

```
## No id variables; using all as measure variables
```

```
df$variable <- factor(df$variable, levels = c("ACC", "SPEC", "SENS"))
```

```
library(ggplot2)
library(ggsci)

ggplot(df, aes(x = variable, y = value, fill = variable)) +
  geom_boxplot(outlier.shape = NA) + scale_y_continuous(limits = c(0.5, 1.01)) +
  geom_jitter(width = 0.2, alpha = 0.3, size = 2) + 
  scale_fill_aaas() +
  theme_bw() + 
  theme(aspect.ratio = 2) + 
  ggtitle("Figure 7e - FlowCapII performance")
```

# Session Info

```
info <- sessionInfo()

info
```

```
## R version 4.3.1 (2023-06-16)
## Platform: x86_64-pc-linux-gnu (64-bit)
## Running under: Ubuntu 22.04.3 LTS
## 
## Matrix products: default
## BLAS:   /usr/lib/x86_64-linux-gnu/openblas-pthread/libblas.so.3 
## LAPACK: /usr/lib/x86_64-linux-gnu/openblas-pthread/libopenblasp-r0.3.20.so;  LAPACK version 3.10.0
## 
## locale:
##  [1] LC_CTYPE=en_US.UTF-8       LC_NUMERIC=C              
##  [3] LC_TIME=en_US.UTF-8        LC_COLLATE=en_US.UTF-8    
##  [5] LC_MONETARY=en_US.UTF-8    LC_MESSAGES=en_US.UTF-8   
##  [7] LC_PAPER=en_US.UTF-8       LC_NAME=C                 
##  [9] LC_ADDRESS=C               LC_TELEPHONE=C            
## [11] LC_MEASUREMENT=en_US.UTF-8 LC_IDENTIFICATION=C       
## 
## time zone: Etc/UTC
## tzcode source: system (glibc)
## 
## attached base packages:
## [1] stats     graphics  grDevices utils     datasets  methods   base     
## 
## other attached packages:
## [1] ggsci_3.0.0    ggplot2_3.4.4  reshape2_1.4.4 cyCONDOR_0.2.0
## 
## loaded via a namespace (and not attached):
##   [1] IRanges_2.34.1              Rmisc_1.5.1                
##   [3] urlchecker_1.0.1            nnet_7.3-19                
##   [5] CytoNorm_2.0.1              TH.data_1.1-2              
##   [7] vctrs_0.6.4                 digest_0.6.33              
##   [9] png_0.1-8                   shape_1.4.6                
##  [11] proxy_0.4-27                slingshot_2.8.0            
##  [13] ggrepel_0.9.4               parallelly_1.36.0          
##  [15] MASS_7.3-60                 httpuv_1.6.12              
##  [17] foreach_1.5.2               BiocGenerics_0.46.0        
##  [19] withr_2.5.1                 ggrastr_1.0.2              
##  [21] xfun_0.40                   ggpubr_0.6.0               
##  [23] ellipsis_0.3.2              survival_3.5-7             
##  [25] memoise_2.0.1               hexbin_1.28.3              
##  [27] ggbeeswarm_0.7.2            RProtoBufLib_2.12.1        
##  [29] princurve_2.1.6             profvis_0.3.8              
##  [31] zoo_1.8-12                  GlobalOptions_0.1.2        
##  [33] DEoptimR_1.1-3              Formula_1.2-5              
##  [35] prettyunits_1.2.0           promises_1.2.1             
##  [37] scatterplot3d_0.3-44        rstatix_0.7.2              
##  [39] globals_0.16.2              ps_1.7.5                   
##  [41] rstudioapi_0.15.0           miniUI_0.1.1.1             
##  [43] generics_0.1.3              ggcyto_1.28.1              
##  [45] base64enc_0.1-3             processx_3.8.2             
##  [47] curl_5.1.0                  S4Vectors_0.38.2           
##  [49] zlibbioc_1.46.0             flowWorkspace_4.12.2       
##  [51] polyclip_1.10-6             randomForest_4.7-1.1       
##  [53] GenomeInfoDbData_1.2.10     RBGL_1.76.0                
##  [55] ncdfFlow_2.46.0             RcppEigen_0.3.3.9.4        
##  [57] xtable_1.8-4                stringr_1.5.0              
##  [59] doParallel_1.0.17           evaluate_0.22              
##  [61] S4Arrays_1.0.6              hms_1.1.3                  
##  [63] glmnet_4.1-8                GenomicRanges_1.52.1       
##  [65] irlba_2.3.5.1               colorspace_2.1-0           
##  [67] harmony_1.1.0               reticulate_1.34.0          
##  [69] readxl_1.4.3                magrittr_2.0.3             
##  [71] lmtest_0.9-40               readr_2.1.4                
##  [73] Rgraphviz_2.44.0            later_1.3.1                
##  [75] lattice_0.22-5              future.apply_1.11.0        
##  [77] robustbase_0.99-0           XML_3.99-0.15              
##  [79] cowplot_1.1.1               matrixStats_1.1.0          
##  [81] xts_0.13.1                  class_7.3-22               
##  [83] Hmisc_5.1-1                 pillar_1.9.0               
##  [85] nlme_3.1-163                iterators_1.0.14           
##  [87] compiler_4.3.1              RSpectra_0.16-1            
##  [89] stringi_1.7.12              gower_1.0.1                
##  [91] minqa_1.2.6                 SummarizedExperiment_1.30.2
##  [93] lubridate_1.9.3             devtools_2.4.5             
##  [95] CytoML_2.12.0               plyr_1.8.9                 
##  [97] crayon_1.5.2                abind_1.4-5                
##  [99] locfit_1.5-9.8              sp_2.1-1                   
## [101] sandwich_3.0-2              pcaMethods_1.92.0          
## [103] dplyr_1.1.3                 codetools_0.2-19           
## [105] multcomp_1.4-25             recipes_1.0.8              
## [107] openssl_2.1.1               Rphenograph_0.99.1         
## [109] TTR_0.24.3                  bslib_0.5.1                
## [111] e1071_1.7-13                destiny_3.14.0             
## [113] GetoptLong_1.0.5            ggplot.multistats_1.0.0    
## [115] mime_0.12                   splines_4.3.1              
## [117] circlize_0.4.15             Rcpp_1.0.11                
## [119] sparseMatrixStats_1.12.2    cellranger_1.1.0           
## [121] knitr_1.44                  utf8_1.2.4                 
## [123] clue_0.3-65                 lme4_1.1-35.1              
## [125] fs_1.6.3                    listenv_0.9.0              
## [127] checkmate_2.3.0             DelayedMatrixStats_1.22.6  
## [129] pkgbuild_1.4.2              ggsignif_0.6.4             
## [131] tibble_3.2.1                Matrix_1.6-1.1             
## [133] rpart.plot_3.1.1            callr_3.7.3                
## [135] tzdb_0.4.0                  tweenr_2.0.2               
## [137] pkgconfig_2.0.3             pheatmap_1.0.12            
## [139] tools_4.3.1                 cachem_1.0.8               
## [141] smoother_1.1                fastmap_1.1.1              
## [143] rmarkdown_2.25              scales_1.2.1               
## [145] grid_4.3.1                  usethis_2.2.2              
## [147] broom_1.0.5                 sass_0.4.7                 
## [149] graph_1.78.0                carData_3.0-5              
## [151] RANN_2.6.1                  rpart_4.1.21               
## [153] farver_2.1.1                yaml_2.3.7                 
## [155] MatrixGenerics_1.12.3       foreign_0.8-85             
## [157] ggthemes_4.2.4              cli_3.6.1                  
## [159] purrr_1.0.2                 stats4_4.3.1               
## [161] lifecycle_1.0.3             uwot_0.1.16                
## [163] askpass_1.2.0               caret_6.0-94               
## [165] Biobase_2.60.0              mvtnorm_1.2-3              
## [167] lava_1.7.3                  sessioninfo_1.2.2          
## [169] backports_1.4.1             cytolib_2.12.1             
## [171] timechange_0.2.0            gtable_0.3.4               
## [173] rjson_0.2.21                umap_0.2.10.0              
## [175] ggridges_0.5.4              parallel_4.3.1             
## [177] pROC_1.18.5                 limma_3.56.2               
## [179] jsonlite_1.8.7              edgeR_3.42.4               
## [181] RcppHNSW_0.5.0              bitops_1.0-7               
## [183] Rtsne_0.16                  FlowSOM_2.8.0              
## [185] ranger_0.16.0               flowCore_2.12.2            
## [187] jquerylib_0.1.4             timeDate_4022.108          
## [189] shiny_1.7.5.1               ConsensusClusterPlus_1.64.0
## [191] htmltools_0.5.6.1           diffcyt_1.20.0             
## [193] glue_1.6.2                  XVector_0.40.0             
## [195] VIM_6.2.2                   RCurl_1.98-1.13            
## [197] gridExtra_2.3               boot_1.3-28.1              
## [199] igraph_1.5.1                TrajectoryUtils_1.8.0      
## [201] R6_2.5.1                    tidyr_1.3.0                
## [203] SingleCellExperiment_1.22.0 labeling_0.4.3             
## [205] vcd_1.4-11                  cluster_2.1.4              
## [207] pkgload_1.3.3               GenomeInfoDb_1.36.4        
## [209] ipred_0.9-14                nloptr_2.0.3               
## [211] DelayedArray_0.26.7         tidyselect_1.2.0           
## [213] vipor_0.4.5                 htmlTable_2.4.2            
## [215] ggforce_0.4.1               CytoDx_1.20.0              
## [217] car_3.1-2                   future_1.33.0              
## [219] ModelMetrics_1.2.2.2        munsell_0.5.0              
## [221] laeken_0.5.2                data.table_1.14.8          
## [223] htmlwidgets_1.6.2           ComplexHeatmap_2.16.0      
## [225] RColorBrewer_1.1-3          rlang_1.1.1                
## [227] remotes_2.4.2.1             colorRamps_2.3.1           
## [229] ggnewscale_0.4.9            fansi_1.0.5                
## [231] hardhat_1.3.0               beeswarm_0.4.0             
## [233] prodlim_2023.08.28
```
