# Supplementary material for: Unveiling the power of high-dimensional cytometry data with cyCONDOR
Source: Nat Commun. 2024 Dec 19;15:10702. doi: 10.1038/s41467-024-55179-w (PMC11659560; doi:10.1038/s41467-024-55179-w)
Supplement: Supplementary file 23 — Reporting Summary [file 41467_2024_55179_MOESM23_ESM.pdf]

nature portfolio

Corresponding author(s): Lorenzo Bonaguro

Last updated by author(s): Sep 16, 2024

# Reporting Summary

Nature Portfolio wishes to improve the reproducibility of the work that we publish. This form provides structure for consistency and transparency in reporting. For further information on Nature Portfolio policies, see our Editorial Policies and the Editorial Policy Checklist.

## Statistics

For all statistical analyses, confirm that the following items are present in the figure legend, table legend, main text, or Methods section.

| n/a | Confirmed | |
|---|---|---|
| ☐ | ☒ | The exact sample size (*n*) for each experimental group/condition, given as a discrete number and unit of measurement |
| ☒ | ☐ | A statement on whether measurements were taken from distinct samples or whether the same sample was measured repeatedly |
| ☐ | ☒ | The statistical test(s) used AND whether they are one- or two-sided<br>*Only common tests should be described solely by name; describe more complex techniques in the Methods section.* |
| ☒ | ☐ | A description of all covariates tested |
| ☐ | ☒ | A description of any assumptions or corrections, such as tests of normality and adjustment for multiple comparisons |
| ☐ | ☒ | A full description of the statistical parameters including central tendency (e.g. means) or other basic estimates (e.g. regression coefficient) AND variation (e.g. standard deviation) or associated estimates of uncertainty (e.g. confidence intervals) |
| ☒ | ☐ | For null hypothesis testing, the test statistic (e.g. *F*, *t*, *r*) with confidence intervals, effect sizes, degrees of freedom and *P* value noted<br>*Give P values as exact values whenever suitable.* |
| ☒ | ☐ | For Bayesian analysis, information on the choice of priors and Markov chain Monte Carlo settings |
| ☒ | ☐ | For hierarchical and complex designs, identification of the appropriate level for tests and full reporting of outcomes |
| ☒ | ☐ | Estimates of effect sizes (e.g. Cohen's *d*, Pearson's *r*), indicating how they were calculated |

*Our web collection on statistics for biologists contains articles on many of the points above.*

## Software and code

Policy information about availability of computer code

| | |
|---|---|
| Data collection | Source of the data used for showcasing the functions and performances of cyCONDOR are available in the methods section. Data from DELCODE healthy control generated for this work fas collected by the DELCODE study group. Raw data are available on FigShare |
| Data analysis | All data were analyzed with cyCONDOR (v.0.2.0) For the comparison with other tools CATALYST v.1.24.0 and SPECTRE v.1.1.0.. Source code of the R package and code for reproducing the figures of the manuscript is available on GitHub (see "Code availability" statement). Script and data necessary to reproduce the analysis are available on FigShare.<br>Github: https://github.com/lorenzobonaguro/cyCONDOR<br>FigShare: 10.6084/m9.figshare.25351981 |

For manuscripts utilizing custom algorithms or software that are central to the research but not yet described in published literature, software must be made available to editors and reviewers. We strongly encourage code deposition in a community repository (e.g. GitHub). See the Nature Portfolio guidelines for submitting code & software for further information.

# Data

Policy information about availability of data

All manuscripts must include a data availability statement. This statement should provide the following information, where applicable:

- Accession codes, unique identifiers, or web links for publicly available datasets
- A description of any restrictions on data availability
- For clinical datasets or third party data, please ensure that the statement adheres to our policy

All data used in this manuscript are publicly available as described in the individual figure, data from DELCODE health donors was generated for this study, raw data are provided on FigShare (https://doi.org/10.6084/m9.figshare.25351981). R environment and data necessary to reproduce the analysis shown in this manuscript are available on FigShare (https://doi.org/10.6084/m9.figshare.25351981 ). We also include for a supplementary file (Supplementary Data 6 to 15) from the compiled script for each figure to provide the user with easy reference to the code used to produce the figure.

# Research involving human participants, their data, or biological material

Policy information about studies with human participants or human data. See also policy information about sex, gender (identity/presentation), and sexual orientation and race, ethnicity and racism.

| | |
|---|---|
| Reporting on sex and gender | Does not apply. All generated data were used only to test out tool and not to make biological assumptions. |
| Reporting on race, ethnicity, or other socially relevant groupings | Does not apply. All generated data were used only to test out tool and not to make biological assumptions. |
| Population characteristics | Does not apply. All generated data were used only to test out tool and not to make biological assumptions. |
| Recruitment | Does not apply. All generated data were used only to test out tool and not to make biological assumptions. |
| Ethics oversight | Analysis of samples from DELCODE study complied with all relevant ethical regulations and was approved by the University of Bonn (Lfd, Nr. 227/19). |

Note that full information on the approval of the study protocol must also be provided in the manuscript.

# Field-specific reporting

Please select the one below that is the best fit for your research. If you are not sure, read the appropriate sections before making your selection.

[x] Life sciences  [ ] Behavioural & social sciences  [ ] Ecological, evolutionary & environmental sciences

For a reference copy of the document with all sections, see nature.com/documents/nr-reporting-summary-flat.pdf

# Life sciences study design

All studies must disclose on these points even when the disclosure is negative.

| | |
|---|---|
| Sample size | Does not apply. All generated data were used only to test out tool and not to make biological assumptions. |
| Data exclusions | Does not apply. All generated data were used only to test out tool and not to make biological assumptions. |
| Replication | Does not apply. All generated data were used only to test out tool and not to make biological assumptions. |
| Randomization | Does not apply. All generated data were used only to test out tool and not to make biological assumptions. |
| Blinding | Does not apply. All generated data were used only to test out tool and not to make biological assumptions. |

# Reporting for specific materials, systems and methods

We require information from authors about some types of materials, experimental systems and methods used in many studies. Here, indicate whether each material, system or method listed is relevant to your study. If you are not sure if a list item applies to your research, read the appropriate section before selecting a response.

## Materials & experimental systems

| n/a | Involved in the study |
| --- | --- |
| ☒ | ☐ Antibodies |
| ☒ | ☐ Eukaryotic cell lines |
| ☒ | ☐ Palaeontology and archaeology |
| ☒ | ☐ Animals and other organisms |
| ☒ | ☐ Clinical data |
| ☒ | ☐ Dual use research of concern |
| ☒ | ☐ Plants |

## Methods

| n/a | Involved in the study |
| --- | --- |
| ☒ | ☐ ChIP-seq |
| ☐ | ☒ Flow cytometry |
| ☒ | ☐ MRI-based neuroimaging |

# Plants

Seed stocks

*Report on the source of all seed stocks or other plant material used. If applicable, state the seed stock centre and catalogue number. If plant specimens were collected from the field, describe the collection location, date and sampling procedures.*

Novel plant genotypes

*Describe the methods by which all novel plant genotypes were produced. This includes those generated by transgenic approaches, gene editing, chemical/radiation-based mutagenesis and hybridization. For transgenic lines, describe the transformation method, the number of independent lines analyzed and the generation upon which experiments were performed. For gene-edited lines, describe the editor used, the endogenous sequence targeted for editing, the targeting guide RNA sequence (if applicable) and how the editor was applied.*

Authentication

*Describe any authentication procedures for each seed stock used or novel genotype generated. Describe any experiments used to assess the effect of a mutation and, where applicable, how potential secondary effects (e.g. second site T-DNA insertions, mosiacism, off-target gene editing) were examined.*

# Flow Cytometry

## Plots

Confirm that:

☐ The axis labels state the marker and fluorochrome used (e.g. CD4-FITC).

☐ The axis scales are clearly visible. Include numbers along axes only for bottom left plot of group (a 'group' is an analysis of identical markers).

☐ All plots are contour plots with outliers or pseudocolor plots.

☐ A numerical value for number of cells or percentage (with statistics) is provided.

## Methodology

| | |
| --- | --- |
| Sample preparation | Cell suspension was stained on ice for 30 minutes with antibody mix before two washed. |
| Instrument | FACS BD S6 |
| Software | Data were recorded with Diva software. All data were analyzed with cyCONDOR 0.2.0 |
| Cell population abundance | We did not quantify as the data were only used for testing of the cyCONDOR tool. |
| Gating strategy | We did not perform conventional gating on the data. Data analysis script can be found of FigShare for reproducibility of the analysis. |

☐ Tick this box to confirm that a figure exemplifying the gating strategy is provided in the Supplementary Information.
